# Ultrasensitive colorimetric detection of fluoride and arsenate in water and mammalian cells using recyclable metal oxacalixarene probe: a lateral flow assay

**DOI:** 10.1038/s41598-022-21407-w

**Published:** 2022-10-12

**Authors:** Shuvankar Dey, Anshu Kumar, Pradip Kumar Mondal, Deepak Chopra, Rupam Roy, Sana Jindani, Bishwajit Ganguly, Chaithra Mayya, Dhiraj Bhatia, Vinod K. Jain

**Affiliations:** 1 Department of Chemistry, School of Sciences, Gujarat University, Ahmedabad, 380009 India; 2 Department of Chemistry, University of Delhi, New Delhi, 110007 India; 3 Elettra-Sincrotrone Trieste, S.S. 14 Km 163.5 in Area Science Park, 34149 Basovizza Trieste, Italy; 4 Department of Chemistry, Indian Institute of Science Education and Research Bhopal, Bhauri, Bhopal, 462066 India; 5 Computation and Simulation Unit (Analytical and Environmental Science Discipline and Centralized Instrument Facility), CSIR-Central Salt and Marine Chemicals Research Institute, Bhavnagar, Gujarat 364002 India; 6 Biological Engineering Discipline, Indian Institute of Technology, Gandhinagar, Gujarat 382355 India; 7 Present Address: Department of Biological Sciences and Bioengineering, Indian Institute of Technology Kanpur, Kanpur, Uttar Pradesh 208016 India; 8 Present Address: Centre for Nano and Material Sciences, Jain University, Jain Global Campus, Bangalore, 562112 India

**Keywords:** Sensors, Supramolecular chemistry

## Abstract

Globally 3 billion people are consuming water with moderately high concentrations of fluoride and arsenic. The development of a simple point of care (PoC) device or home device for the detection of fluoride/arsenic ensures safety before consuming water. Till date, lateral flow assay (LFA) based PoC devices can detect nucleic acids, viruses and diseases. An aluminium complex of rhodamine B functionalized oxacalix[4]arene (**L**) was designed to execute the LFA-based PoC device. Initially, Al^3+^ and Fe^3+^ ions were involved in complexation with the rhodamine B functionalized oxacalix[4]arene (**L**), resulting **C**_**1**_ (L-Al^3+^) and **C**_**2**_ (L-Fe^3+^) complexes respectively. The receptor **L**, as well as the probes (**C**_**1**_**, C**_**2**_), were characterized thoroughly using mass spectroscopy, FTIR, NMR, and EA. **C**_**1**_ and **C**_**2**_ were further utilized as recyclable probes for the detection of aqueous fluoride (21 ppb) and arsenate (1.92 ppb) respectively. The computational calculation indicates that upon complexation, the spirolactam ring opening at the rhodamine B site leads to optoelectronic changes. The consistency of LFA-based portable sensing device has been tested with water samples, synthetic fluoride standards and dental care products like toothpaste and mouthwash with concentrations ≥ 3 ppm. Moreover, fixed cell imaging experiments were performed to ascertain the in-vitro sensing phenomena.

## Introduction

Contamination of drinking water with fluoride and arsenic ions poses a threat to global health. According to the World Health Organization (WHO) and Environmental Protection Agency (USEPA), 140 million people have been exposed worldwide to arsenic contamination of > 10 ppb in drinking water and 2.8 million people may have been exposed to drinking water with fluoride contamination of more than 2 ppm^1–3^. Fluoride and both species of arsenic (As^3+^ and As^5+^) were found in environmental samples as inorganic salts^4^. Arsenic (As) is considered to be one of the most carcinogenic and toxic elements that exist in earth’s crust and groundwater^5^. Long-term exposure to fluoride concentrations above 2 ppm can cause osteoporosis, urolithiasis, fluorosis, neurological, metabolic dysfunctions, and even cancer^6^. Fluoride is also biologically and medically important for its essential roles in proper growth and maintenance of teeth, hair, nails, and bones and treatment of osteoporosis^7^. Countries of South-East Asia, including Bangladesh, China, and India, are severely affected by these two ions. In India, people residing in the middle and lower Gangetic planes and some Central and South-India areas characterized by hard rock terrain are worse affected by arsenic and fluoride contamination^8,9^. Contamination of groundwater with arsenic and fluoride mainly occurs due to the dissolution of rocks and soils in water. Continuous increase in the concentration of these anions creates a major problem in the safe drinking water supply^10^. Outcome of surveys that have been undertaken to assess the groundwater quality indicates that a large proportion of people all over the world regularly intake fluoride and arsenic-contaminated water above maximum concentration limit due to lack of awareness and unavailability of simple detection methods^11^. Therefore, there is an urgent need to develop a sensing platform that is easy-to-use and collects fast and reliable data on water quality.

The development of ultrasensitive assays based on colorimetric and fluorescence probes has got immense attention during the last couple of decades due to their large optical field enhancement properties that result in the strong scattering and absorption of light^12,13^. Additionally, the field of biochemical sensors has witnessed an explosion because of its unique optical as well as fluorometric properties^14,15^. Most recently, a few fluorophore-based assay^16–18^ and colorimetric sensors^19,20^ have been reported for fluoride and arsenate detection down to 2 ppm and 10 ppb respectively, which is an order of magnitude lower than WHO guidelines^21,22^. On similar lines, oxacalix[4]arene has emerged as a versatile probe due to its tunable cavity, ease of derivatization and excellent selectivity over a wide range of competitive analytes^23,24^. Very few articles have been reported for the detection of both fluoride and arsenic ions in aqueous medium based on supramolecular probes^25^.

**Figure 1:**
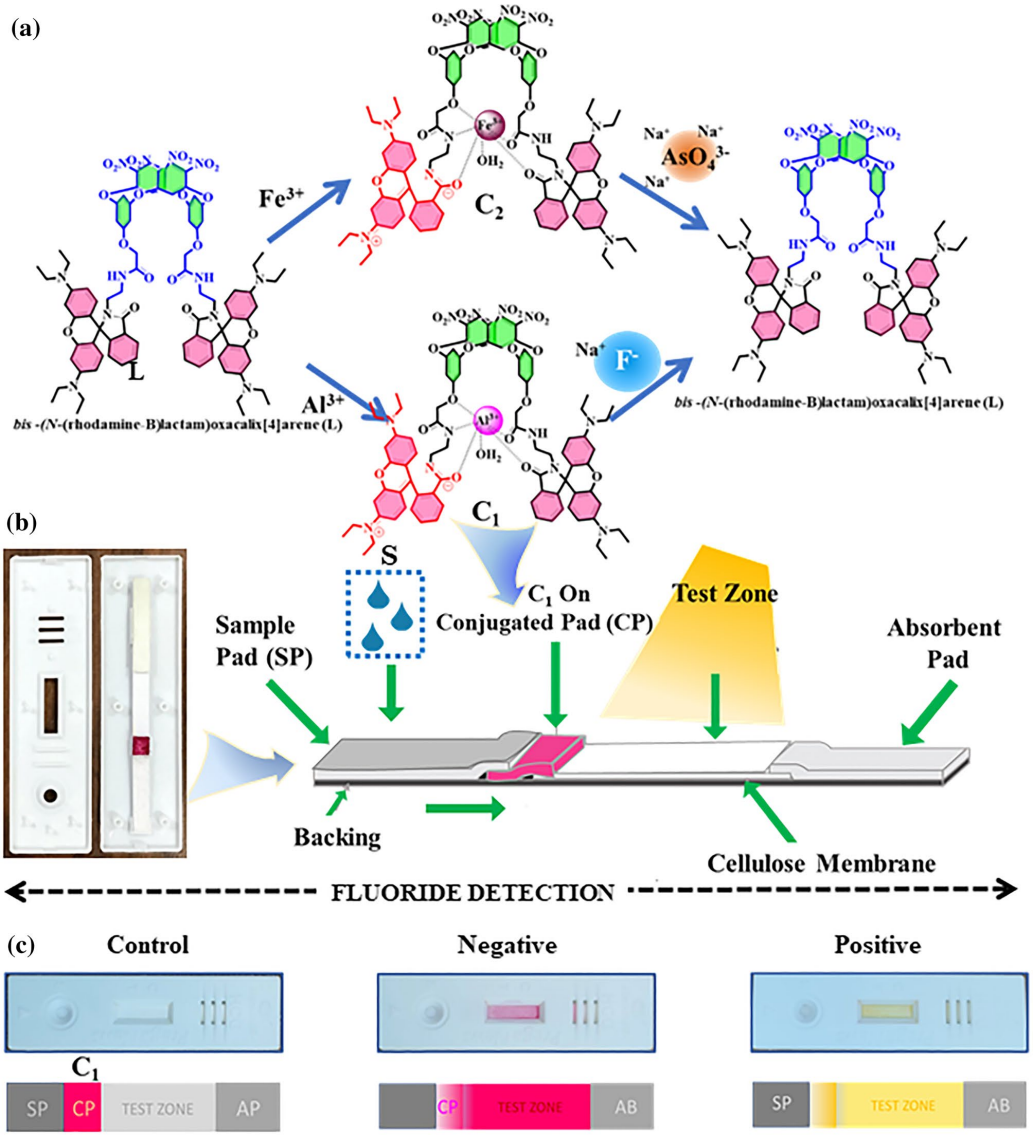
(**a**) Al^3+^/Fe^3+^ induced sensing of fluoride and arsenate respectively; (**b**) Schematic illustration of LFA-based PoC device prototype; (**c**) Representation of positive and negative results in LFA toward the detection of fluoride.

Herein, we report the design and synthesis of rhodamine B functionalized novel oxacalix[4]arene architecture (**L**). Our primary objective in this study is to develop a probe for the ultrasensitive detection and quantification of fluoride and arsenic ions in aqueous medium. To achieve this target, we have initially prepared complexes of trivalent metal ions (Al^3+^ and Fe^3+^). This leads to the spirolactam ring opening, resulting in an enhancement in emission maxima with a significant change in fluorescent color from non-fluorescent to bright orange. The probes, **C**_**1**_ (L-Al^3+^) and **C**_**2**_ (L-Fe^3+^) were utilized for the selective detection of sodium fluoride and sodium arsenate (Fig. 1a), in water and mammalian cells, which facilitated the breakdown of L-Al^3+^ and L-Fe^3+^ complexes, that prompted PET “ON” response. We have successfully demonstrated the recycling of receptor **L** up to 5 cycles. The probe **C**_**1**_ was utilized to fabricate LFA based point of care device for the real-time detection of fluoride ions in environmental water samples (Fig. 1b,c). Moreover, **C**_**1**_ and **C**_**2**_ probes were employed for fixed cell imaging and the determination of unknown concentrations of fluoride and arsenate in tap water, groundwater and seawater.

## Materials

The starting materials of the receptor including 1,5-difluoro-2,4-dinitrobenzene; phloroglucinol; ethylenediamine, rhodamine B and all the chemicals of analytical grade like metal salts, anions were purchased from Sigma-Aldrich. The solvents used to carry out the synthesis, purification and analytical experiments were purchased from Finar Chemicals and were used without further purification. Milli-Q water was used to prepare the solutions for performing the analytical experiments. During the synthesis of receptor and its precursors, the progress of the reactions was monitored by thin-layer chromatography (TLC) using E-Merck silica gel 60 F_254_ pre-coated plates, which were visualized with UV-light (254 nm).

## Methods

### Synthesis of receptor L

**Scheme 1:**
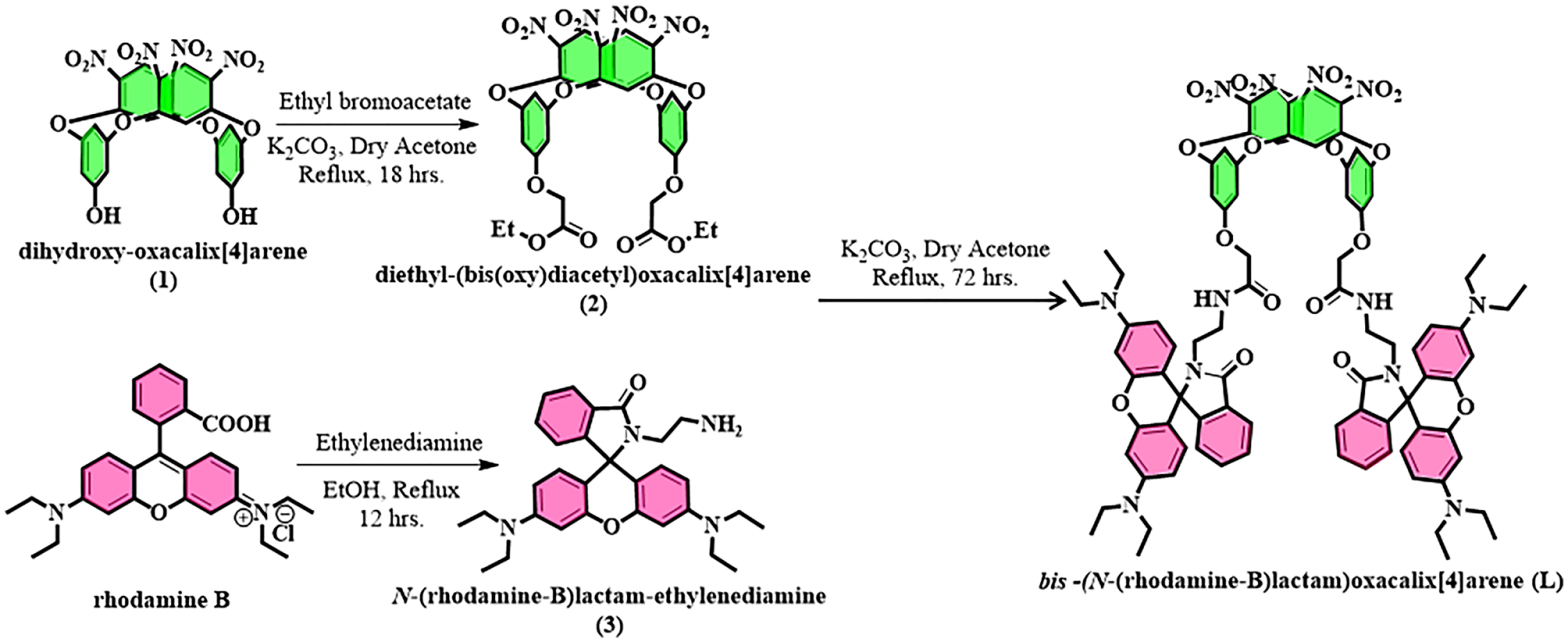
Synthetic scheme of the preparation of receptor L.

The receptor **L** was synthesized followed by several consecutive steps starting from parent oxacalix[4]arene (**1**), as shown in Scheme 1. In detail, synthesis procedures have been discussed in the ESI (Section S2–S4). Initially, the parent oxacalix[4]arene (compound **1**) was reacted with ethyl bromoacetate to introduce ester groups at the terminal (compound **2**). Parallelly, a mixture of rhodamine B and ethylenediamine was refluxed for 12 h to obtain *N*-(rhodamine-B)lactam-ethylenediamine (compound **3**). Finally, to a solution of compound **2** (1 g, 1.3 mmol) in dry acetone, K_2_CO_3_ (0.37 g, 2.6 mmol) and compound **3** (1.3 g, 2.6 mmol) were added. Then the reaction mixture was allowed to reflux for 72 h. After 3 days of refluxing, the reaction mixture was cooled to room temperature, neutralize, and washed with water. The organic layer was extracted in ethyl acetate, dried over anhydrous Na_2_SO_4_ and the solvents were removed in vacuum. The crude was then purified with column chromatography (10% EtOAc in hexane) to afford an orange solid of receptor *bis*-(*N-*(rhodamine-B)lactam)oxacalix[4]arene (**L)**.

### Preparation of fluoride and arsenate sensing probes (C_1_ and C_2_)

In a 25 ml round bottom flask, ethanolic solutions of aluminium (III) nitrate (1.0 mmol) and iron (III) nitrate (1.0 mmol) were separately added to the ethanolic suspension (5 ml) of ligand **L** (1.0 mmol). The reaction mixtures were stirred for 6 h at room temperature. The completion of the reactions was confirmed by the color change of the solutions from pale yellow to pinkish red. The reaction mixtures were then dried in vacuum to obtain the pinkish red-colored complexes of L-Al^3+^ (**C**_**1**_) and L-Fe^3+^ (**C**_**2**_).

### Crystallization and data collection

15 mg of compound **1** was dissolved in a small amount of MeOH (HPLC grade). An equal volume of hexane (HPLC grade) was gently added to it. Then the beaker was placed in a refrigerator maintaining 4–5 °C temperature without any perturbation. After 12 days, plate-like crystals were observed. A suitable crystal was then mounted on the tip of glass fibre. Single crystal X-ray diffraction measurements for compound **1** were carried out using a Bruker APEX II Kappa CCD single crystal diffractometer equipped with a graphite monochromator using Mo Ka radiation (λ = 0.71073 Å) at 140(3) K. Unit cell measurements, data collection, integration, scaling, and absorption corrections were performed using the Bruker APEX II software^26^. Multiscan absorption corrections were applied using SADABS^27^. The crystal structure was solved by direct methods using SHELXS^28^ and the crystal structure was finally refined through OLEX2^29^. All non-hydrogen atoms were refined anisotropically, and all hydrogen atoms bound to carbon and oxygen were placed in the calculated positions.

### Absorption and fluorescence measurements of the receptor L

A stock solution of the receptor L (1 mM) was prepared in acetonitrile and diluted to 50 μM for absorption and fluorescence studies. All the stock solution including metal ions (Fe^3+^, Al^3+^, Fe^2+^, Mn^2+^, Co^2+^, Ni^2+^, Cu^2+^, Zn^2+^, Cd^2+^, Hg^2+^, Cr^3+^, Sr^2+^, Ba^2+^, Pb^2+^) and anions (F^−^, Cl^−^, Br^−^, I^−^, ClO_4_^−^, PO_4_^3−^, AsO_4_^3−^, NO_3_^−^, S_2_O_3_^2−^, HSO_4_^−^, HPO_4_^2−^) were prepared in acetonitrile (1 mM) and diluted to 100 μL for photochemical measurements. The absorption spectra were recorded within a range of 200–800 nm after incubation for 3 min at room temperature. On similar lines, under optimized conditions, the solution was excited at 313 nm and the emission measurements were carried out within a range of 350–700 nm keeping the excitation and emission slit width of 5 nm. In order to obtain a better visual range of detection, 50 µM methylene blue (MB) dye has been added. Figure S17 further demonstrates that no significant change in absorption maxima was observed upon addition of an optimized amount of MB dye.

### Absorption and fluorescence measurements of L-M^3+^ probes

1 mM stock solution of the probe **C**_**1**_ and **C**_**2**_ were prepared in acetonitrile and diluted to 50 μM for absorption and emission studies. The stock solution of anions (F^−^, Cl^−^, Br^−^, I^−^, ClO_4_^−^, PO_4_^3−^, AsO_4_^3−^, NO_3_^−^, S_2_O_3_^2−^, HSO_4_^−^, HPO_4_^2−^) were prepared in Milli Q water (1 mM) and diluted to 50 μL for photochemical measurements. The absorption spectra were recorded within a range of 200–800 nm after incubation for 2 min at room temperature (Figure S18). On similar lines, under optimized conditions, the solution was excited at 313 nm and the emission measurements were carried out within a range of 350–700 nm keeping the excitation and emission slit width of 5 nm.

### Computational methods

Geometrical optimization was carried out for the ground state (S0) and the singlet excited state (S1) of L and L-Al^3+^ systems using HF/3-21G*^30^ method using the Gaussian 09 program^31^. Harmonic vibrational frequency calculations were found to be positive that confirming all the stationary points as minima. The time-dependent density functional theory (TD-DFT)^32,33^ approach was employed to investigate UV–Vis absorption and emission transition properties for optimized ground and excited state geometries. Frontier molecular orbital analysis was performed to examine the transitions in UV–Vis spectral results.

### Time-resolved fluorescence measurements

The fluorescence lifetime measurement of all compounds was carried out in spectroscopic grade ACN using the same 1 cm path-length cuvette and the decay was monitored on the emission maxima of the compounds. Excited-state lifetime value was determined using time-correlated single photon counting (TCSPC) experimental setup from Hamamatsu MCP photomultiplier (R-3809U-50). Photon count was fixed up to 10,000 and 468 nm pico-second laser was applied to excite the compounds in solution. Before measurement of decay, instrument response function (IRF) was measured using a dilute solution of Ludox, a suspension of colloidal silica. During measurement, excitation and emission polarizer was fixed at vertically and magic angle (54.7°) respectively. The tri-exponential decay was obtained using deconvolution method in EZ time software. All fittings were done by keeping the χ value within the range 0.95–1.4. The excited state average fluorescence lifetime (*τ*_avg_) was calculated using Eq. (1).

1 $${\tau }_{avg}=\sum {a}_{i}{\tau }_{i}^{2}/\sum {a}_{i}{\tau }_{i}$$

where *a*_*i*_ denotes the contribution of ith component and *τ*_*i*_ indicates lifetime value of ith component.

### Fixed cell imaging

A triple-negative breast cancer cell line (SUM159) was selected for fixed cell imaging experiments. The SUM159 cells were cultured in Hams F12 media supplemented with 5% Fetal Bovine Serum (Gibco), 1 μg/ml Hydrocortisone, 5 μg/ml Insulin and 10 mM Hepes buffer (Sigma). For fixed cell imaging, the cells were grown on the coverslips for 24–36 h at 37 °C, 5% humidity before the experiment. The cells were then treated with the ligand (L) for 30 min at 10 μM concentration at cell culture conditions. iron (III) nitrate (Fe(NO_3_)_3_·9H_2_O) and aluminium perchlorate (Al(ClO_4_)_3_·9H_2_O) solutions at 10 μM concentrations, were added separately to the cells after the treatment of ligand and additional 15 min of incubation was carried out at cell culture conditions. These iron and aluminium treated cells were further treated with 20 μM sodium arsenate (Na_2_HAsO_4_·7H_2_O) and 30 μM sodium fluoride (NaF) solutions respectively for 15 min at cell culture conditions. The cells were then washed thoroughly and fixed using 4% Paraformaldehyde (Merck). The imaging of the cells was carried out in Leica laser confocal microscope (model: TCS SP8).

### Lateral flow assay (LFA)

LFA strips consist of four components: the sample, absorbent pads, cellulose (CSE) membrane and conjugate pad (CP). Firstly, both the sample and absorbent pads were saturated with tris buffer. The conjugate pad was saturated with pre-treatment buffer and then dried for 2 h at room temperature. The probe **C**_**1**_ was resuspended in acetonitrile and loaded onto the saturated conjugate pad. Finally, the pads were assembled in an overlapping sequence, using the adhesive tape at both ends of the CSE membrane, and were cut to a width of 4 mm. Prepared LFA strips were stored with a desiccant at 4 °C until use. 100 μL aqueous solution of sodium fluoride (1–10 ppm) was dispensed onto the LFA sample pad at room temperature. As a control, we used only the acetonitrile (blank) and aqueous solution. The appearance of the test zone was monitored for 3 min. The **C**_**1**_-based LFA was also evaluated in fluoride-containing real samples as well as in fluoride mixtures prepared by mixing fluoride and other anions at various ratios in the dilution in the same way as described above.

## Result and discussion

### Crystallographic study of parent oxacalix[4]arene (Compound 1)

**Figure 2:**
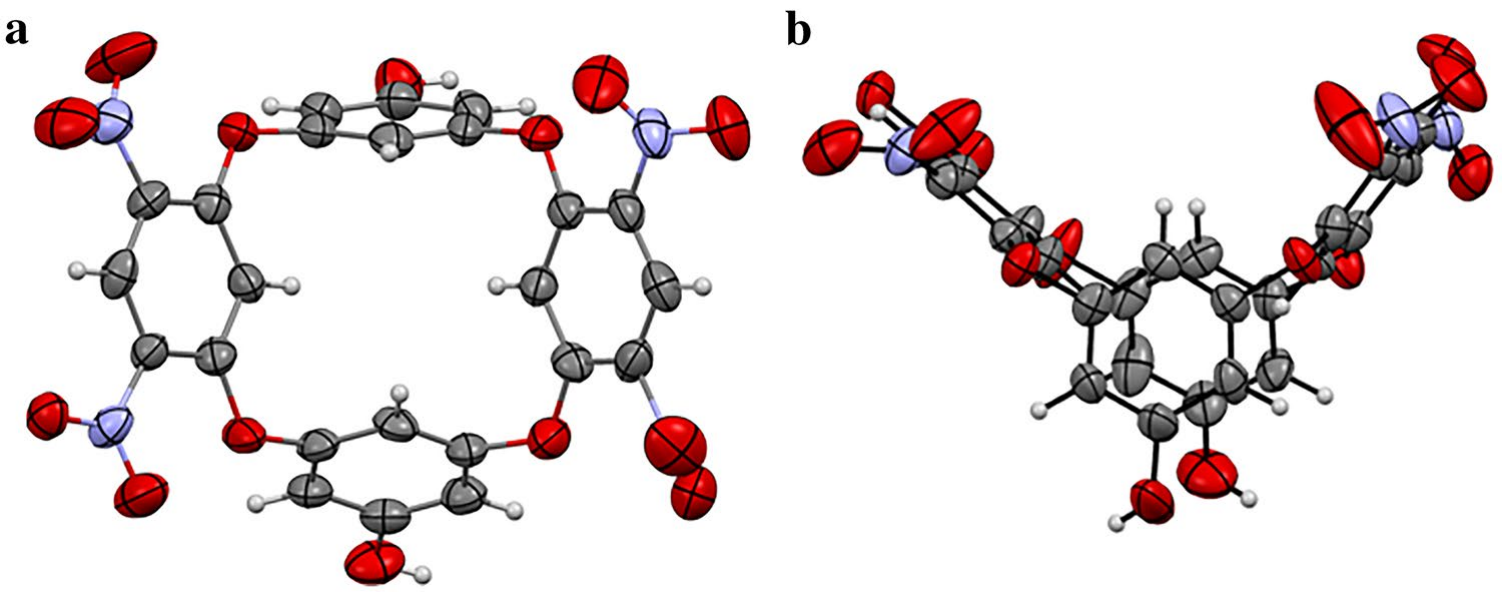
*ORTEP* of compound 1 with 50% ellipsoid probability (**a**) top view and (**b**) front view.

X-ray quality crystals of compound **1** were obtained by liquid diffusion of hexane into methanol. Single crystal analysis reveals that compound 1 crystallizes in a triclinic system with a = 14.3488(9), b = 15.0188(9), c = 15.2848(10) and α = 97.162(5), β = 92.241(4), γ = 101.857(4). The space group was found to be *P*$\overline{1 }$. The asymmetric unit consists of two molecules of compound **1** (represented as blue and green) and two molecules of solvent MeOH (represented as red and yellow) (Fig. S19). The green molecule of compound 1 present in the asymmetric unit interacted with red and yellow methanol via motif II and motif I respectively. The crystal data and structure refinement parameters are given in Table S1. The *ORTEP* of compound 1 is shown in Fig. 2 and the list of intermolecular interactions are shown in Table S2. Alike other oxacalix[4]arenes derived from alternative electrophile, compound **1** adopts a distorted 1,3-alternate conformation in its solid state^34,35^. The electrophile aromatic ring planes are nearly perpendicular to each other, (angle between the ring planes: green—89.96°, blue—86.77°), while the angle between nucleophilic ring planes was calculated to be ~ 45° (green—43.82°, blue—44.41°) with the hydroxy groups pointing opposite to each other. Analogues to previously reported basic oxacalix[4]arene, the average bond distance from the bridging O-atom to the electrophile-derived aromatic ring was found to be significantly shorter (1.35 Å) in comparison to the equivalent bonds of the nucleophile-derived ring (1.40 Å)^36,37^. The intermolecular H-bonding formed between the green set of compound 1 and red methanol molecules influences the formation of a 16 member ring (motif II and IV) and a 24 member ring (motif II, III) as depicted in Fig. S20, Table S2. Similarly, the blue set molecules in compound 1 are connected via the formation of 10- (motif VII) and 20-membered ring (motif VIII) through weak van der Waals interactions (Fig. S21).

### Design of chemosensor

It has been already mentioned that excessive intake of fluoride and arsenate can result in fluorosis, heart disease, urolithiasis or even cancer. Therefore, on spot recognition of these anions in aqueous medium has received considerable attention in recent times. Chromogenic changes during sensing of guest analytes play an important role in sensing applications. In this regard, a rhodamine B functionalized novel oxacalix[4]arene receptor **L** has been designed as a dual responsive “*ON–OFF*” sensor for selective intercellular sensing of fluoride and arsenate via **C**_**1**_ and **C**_**2**_ probes respectively. This work mainly focuses on the trivalent metal ion (Al^3+^ /Fe^3+^) induced ring-opening of rhodamine unit. Herein, the intense overlap between ligand emission and ring-opened rhodamine B spectra reinforces the energy transfer process (Fig. S22). Therefore, rhodamine B has been functionalized over the calixarene intermediate viz. *di(ethoxycarbonyl)oxacalix[4]arene* to investigate the anion binding properties.

### Response toward metal ions

**Figure 3:**
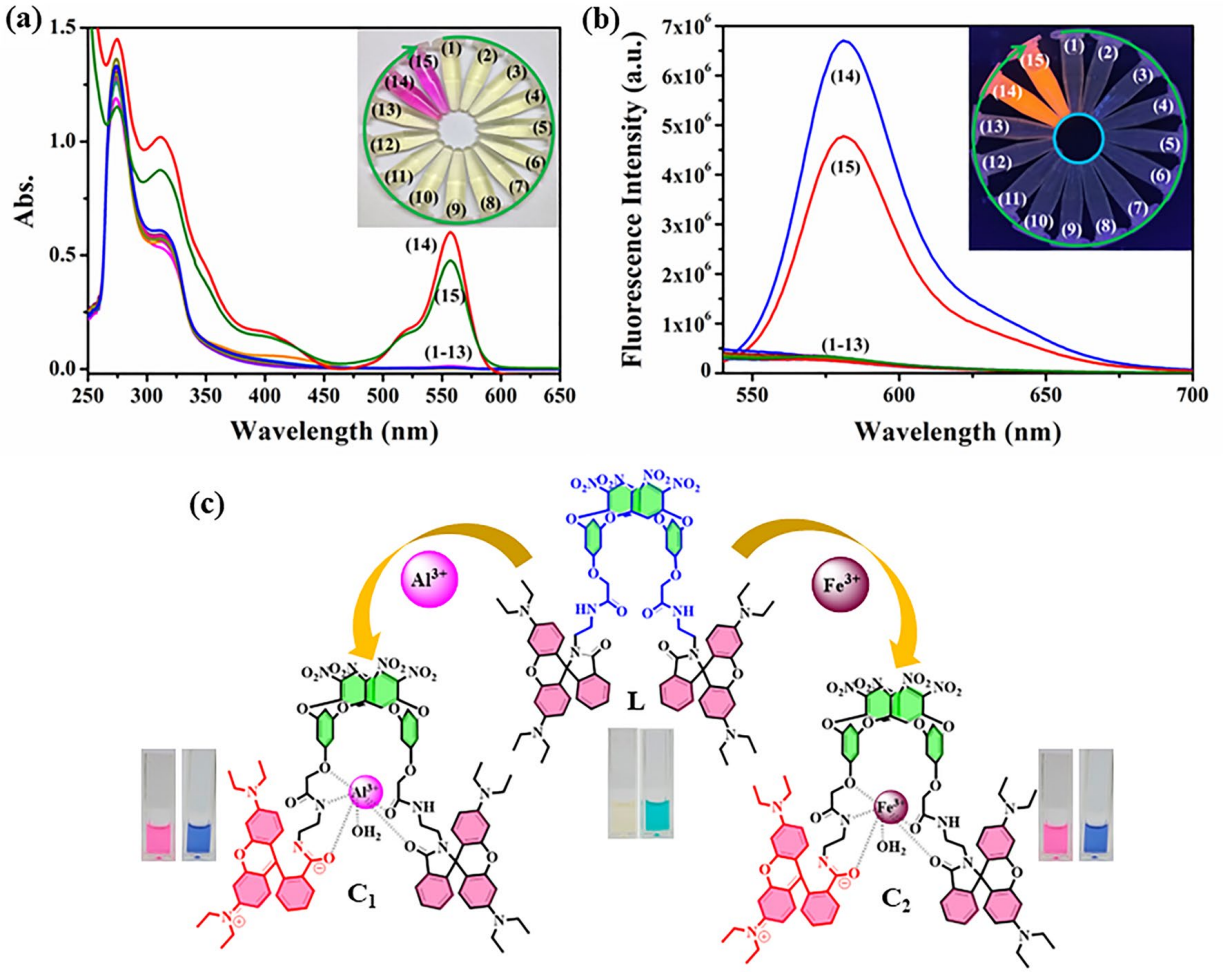
(**a**) Change in absorption maxima of the receptor L toward various metal ions, Inset: colorimetric changes; (**b**) Fluorescence responses of L with different metal ions (1–13) Fe^3+^ (14) and Al^3+^ (15), Inset: color changes under UV light; (**c**) Schematic representation for the binding of Al^3+^ and Fe^3+^.

Among a series of 14 common metal ions used to explore the binding interactions with receptor **L**, only Fe^3+^ and Al^3+^ display a distinct color change from pale yellow to pinkish red. Moreover, to investigate the sensing behavior in detail, absorption and emission studies were conducted to observe the photophysical changes during interaction. The receptor **L** exhibits maximum absorption at 274 nm with a hump at 313 nm. Under optimized condition (25 °C, 3 min., Fig. S23), the change in absorption maxima were recorded upon interaction with the aforementioned ions. A new absorption band appears at 559 nm only in the presence of Fe^3+^ and Al^3+^ (Fig. 3a). This may be due to the spirolactam ring opening at the rhodamine B centre, which enables suitable binding towards hard metal ions. Moreover, the receptor **L** exhibits a weak fluorescence at 498 nm (λ_ex_ = 313 nm, Fig. S24). Significant enhancement in fluorescent intensity (λ_max_ = 582 nm) only in the presence of Fe^3+^ and Al^3+^ indicates stable complexation with these metal ions (Fig. 3b). A schematic representation of the binding of metal ions is depicted in Fig. 3c. Interestingly, no substantial change in photophysical properties of the receptor L was observed with a series of common anions. Competitive experiments pertaining to the coexisting ions indicate that only sodium fluoride and sodium arsenate influence **C**_**1**_ (L-Al^3+^) and **C**_**2**_ (L-Fe^3+^) systems respectively. A detailed discussion about this behaviour has been discussed in the next section.

**Figure 4:**
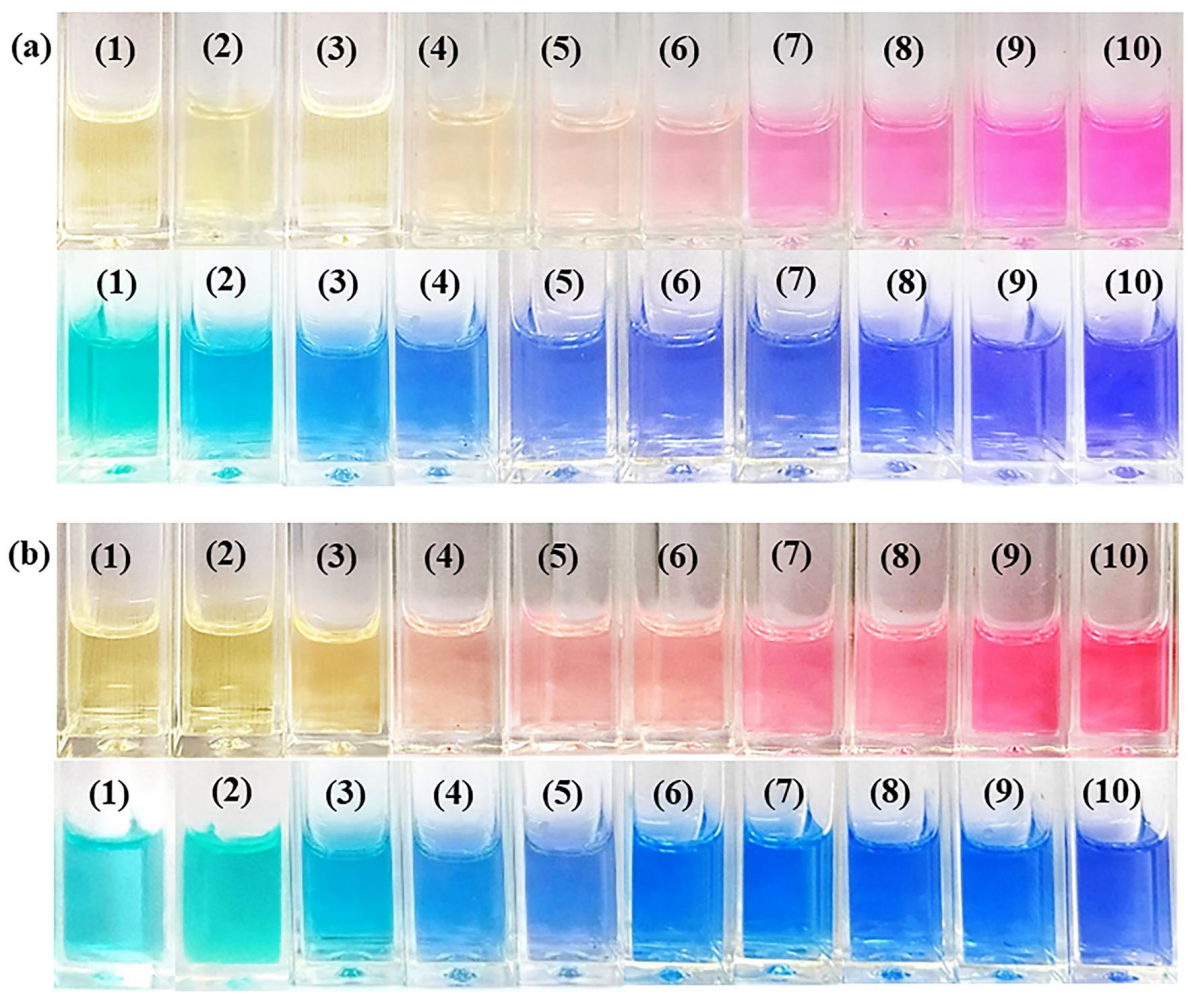
Change in color of the receptor L upon increasing concentration of Al^3+^ (**a**, Inset: concentrations 1 → 0 nM, 2 → 100 nM, 3 → 200 nM, 4 → 300 nM, 5 → 400 nM, 6 → 500 nM, 7 → 600 nM, 8 → 700 nM, 9 → 800 nM, 10 → 900 nM) and Fe^3+^ (**b**, Inset: concentrations 1 → 0 nM, 2 → 25 nM, 3 → 50 nM, 4 → 75 nM, 5 → 100 nM, 6 → 150 nM, 7 → 200 nM, 8 → 300 nM, 9 → 400 nM, 10 → 500 nM) in the top panel. Addition of equal proportion of methylene blue dye onto each test solution (bottom panel).

In order to gain in-depth insight into the binding assay, stoichiometry and sensing behavior of these trivalent metal ions, absorption and emission titration experiments were carried out under optimized conditions. Upon sequential addition of Al^3+^ (200–1200 nM) and Fe^3+^( 0–450 nM) to the receptor **L**, results a significant enhancement in emission intensity at 582 nm for both the complexes (Fig. S25a,b). The Stern–Volmer plot reveals that these fluorescent probes significantly recognize Al^3+^ and Fe^3+^ over a wide concentration range, as the linear range of detection was determined to be 300–900 nM (R^2^ = 0.9958) and 50–250 nM (R^2^ = 0.9930) respectively (Fig. S26). However, the visual colour change for Al^3+^ and Fe^3+^ can only be observed from 300 and 75 nM respectively (Fig. 4). Interestingly, the use of a preoptimized amount of methylene blue dye helps to lower the visual detection up to 200 nM and 50 nM for Al^3+^ and Fe^3+^ respectively. It is noteworthy to mention that the addition of preoptimized methylene blue doesn’t influence the receptor or ions (Fig. S27), it is only taken as chromogenic purpose to improve the range of visual detection. The Job’s plot analysis further confirms 1:1 complexation with both the analytes (Fig. S28).

### Detection of NaF and Na_3_AsO_4_ in aqueous medium

**Figure 5:**
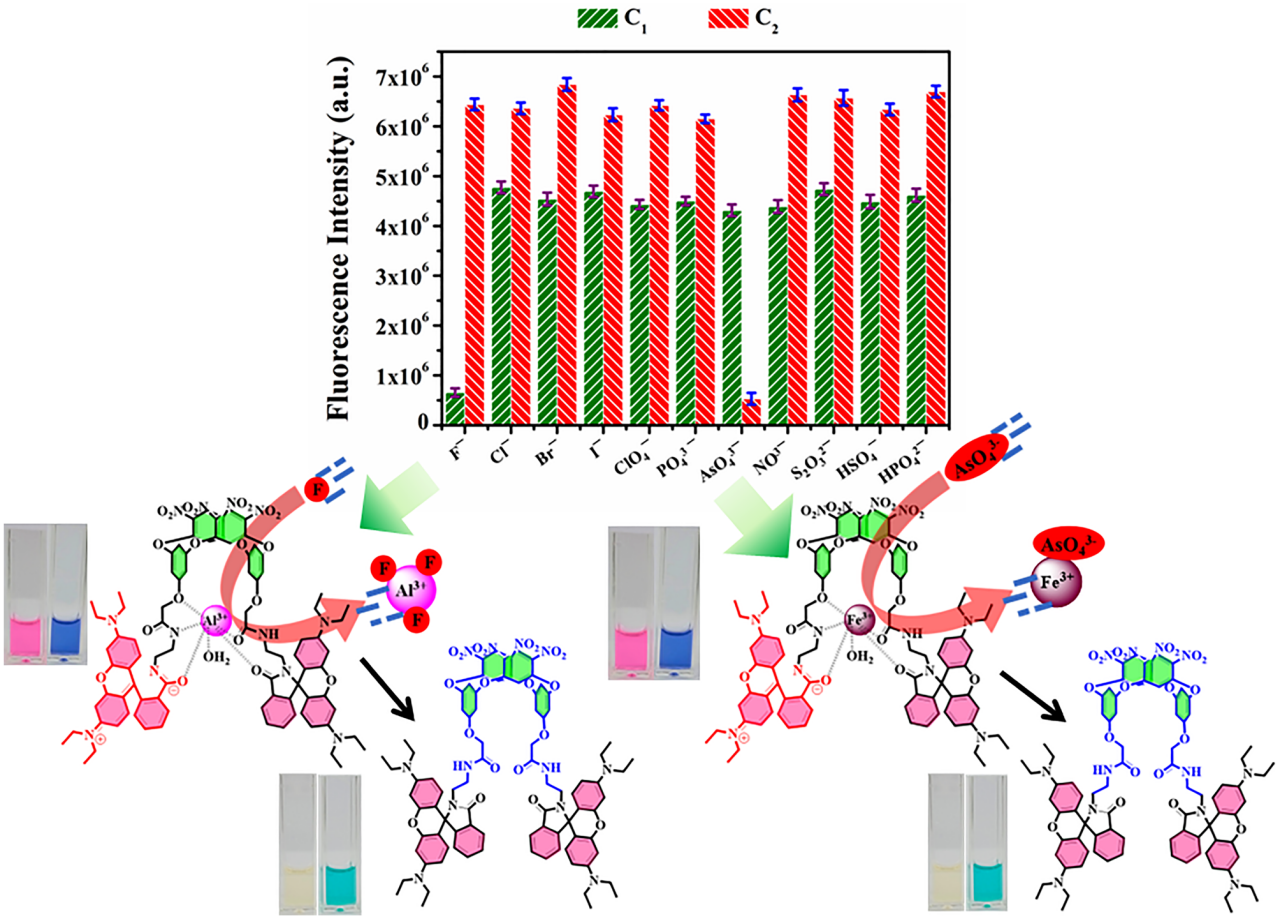
Variation in emission responses of C_1_ and C_2_ against a series of common anions in aqueous medium (top); a pictorial representation of fluoride and arsenate anion interacts C_1_ and C_2_ respectively.

The probes **C**_**1**_ and **C**_**2**_ were employed to study the sensing responses toward anions in aqueous medium. The initial observation indicates that **C**_**1**_ exhibits the potential to substantially interact with fluoride (F^−^), whereas **C**_**2**_ successively binds with arsenate (AsO_4_^3−^) followed by the naked eye color change from pinkish red to pale yellow among 11 most common anions (Fig. 5). To investigate the photochemical responses in detail, absorption and emission responses of **C**_**1**_ and **C**_**2**_ were recorded under optimized conditions (25 °C, pH 7.4, 3 min) with a series of water-soluble common anions. The consequences of absorption study indicate a gradual suppression in absorption band of **C**_**1**_ and **C**_**2**_ at 559 nm only in presence of fluoride and arsenate respectively. Likewise, a quenching in emission maxima of **C**_**1**_ and **C**_**2**_ was observed only with the fluoride and arsenate respectively, while no such quenching in fluorescence intensity was noticed with the other coexisting anions (Fig. S30). These experiments were repeated 3 times and the outcome demonstrates the extreme selectivity of **C**_**1**_ and **C**_**2**_ towards fluoride and arsenate respectively over a series of 11 common anions. This phenomenon may be attributed to the formation of aluminium fluoride and ferric arsenate either by the complete breakdown of the respective complexes or by weakly attached to it, ultimately leading to a less conjugated system due to the reformation of spirolactam ring. Moreover, no significant influences of competitive anions were observed while **C**_**1**_ and **C**_**2**_ were employed for the sensing of fluoride and arsenate respectively. This experiment further validates the suitability of the systems toward the sensing of arsenate and fluoride.

### Sensitivity toward fluoride and arsenate

**Figure 6:**
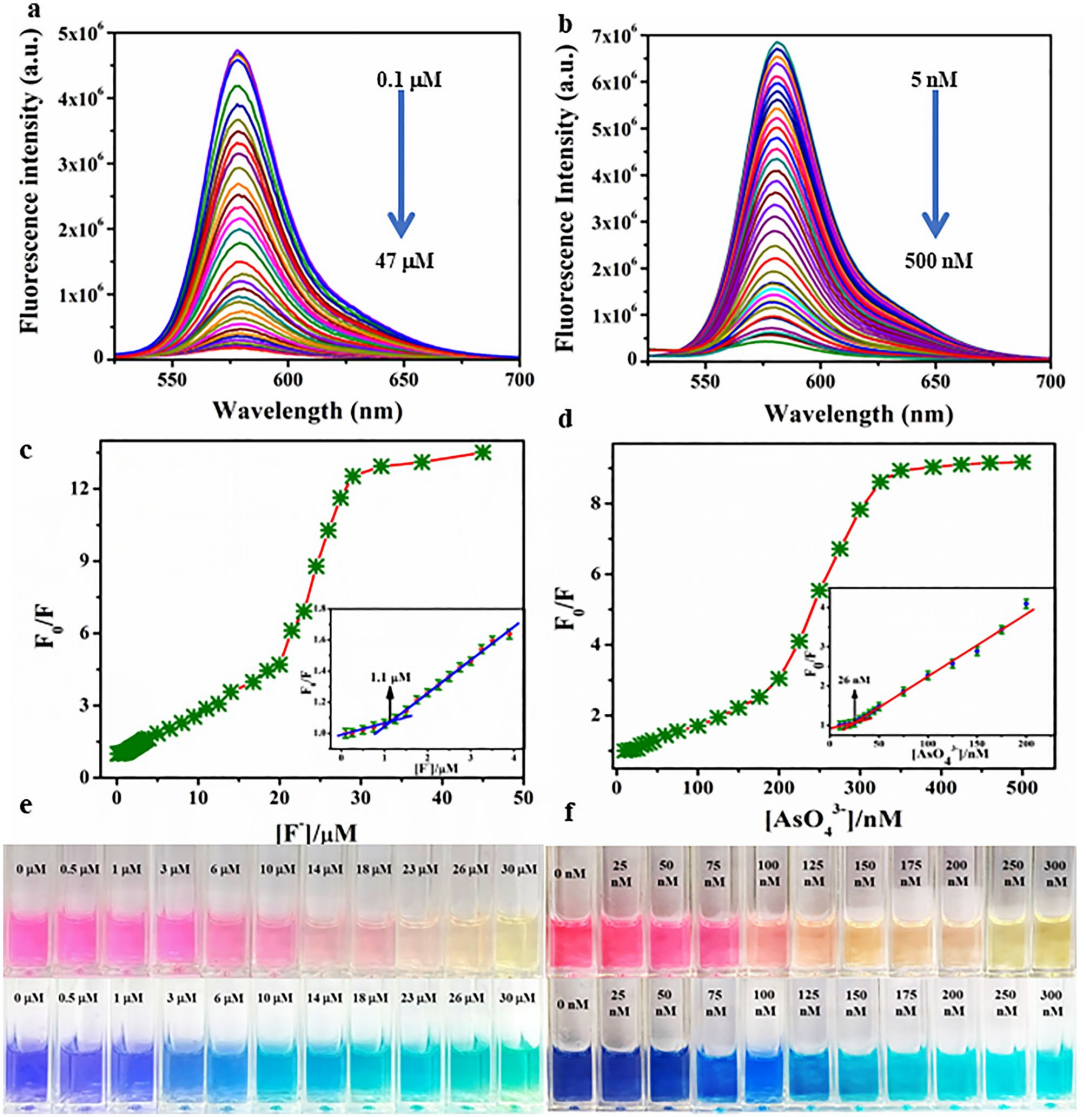
Fluorescence titration (**a**) Change in emission intensity of C_1_ with the addition of an increasing amount of fluoride (0.1–47 μM), (**b**) Change in emission intensity of C_2_ with the addition of an increasing amount of arsenate (5–500 nM), (**c**) Stern–Volmer plot for fluoride, Inset: LOD, (**d**) Stern–Volmer plot for arsenate, Inset: LOD; Change in color of C_1_ (**e**) and C_2_ (**f**) upon incremental addition of fluoride and arsenate respectively (top), the addition of an equal proportion of methylene blue dye onto each solution (bottom).

Further to assess the sensitivity of probes **C**_**1**_ and **C**_**2**_, towards fluoride and arsenate, emission titration experiments were carried out. Upon sequential addition of F^-^ (0.1–47 μM) and AsO_4_^3−^ (5–500 nM) to the probe **C**_**1**_ and **C**_**2**_ respectively, results in a significant quenching in emission intensity at 582 nm for both the complexes (Fig. 6a,b). A plot of F_0_/F versus anion concentration has been depicted in Fig. 6c,d. The binding constants of **C**_**1**_ and **C**_**2**_ probes toward respective ions were determined using the Stern Volmer equation (Eq. 2) in the order of 10^2^ and considered to be moderate binding for both systems.

2 $$\mathrm{log}\frac{{\mathrm{F}}_{0}-\mathrm{F}}{\mathrm{F}}=\mathrm{log }{\mathrm{K}}_{\mathrm{b}}+\mathrm{nlog}[\mathrm{Q}]$$

Here, F_0_ represents the emission intensity of the probes **C**_**1**_ and **C**_**2**_, F stands for the emission intensity in presence of analytes. Q is denoted as the concentration of analytes in mole, K_b_ signifies binding constant and n represents the number of binding sites. The LOD for fluoride and arsenic was determined to be 1.1 µM and 26 nM respectively (based on the 3σ method). The Stern–Volmer plot reveals that these fluorescent probes significantly detect fluoride and arsenate over a wide concentration range, as the linear range of detection was determined to be 2–18.5 μM (R^2^ = 0.99286) and 30–175 nM (R^2^ = 0.99124) respectively (Figs. S31a, S32a). It is noteworthy to mention that the visual colour changes of **C**_**1**_ and **C**_**2**_ can be identified from 6 µM and 100 nM of fluoride and arsenate respectively (Fig. 6e,f). However, the use of methylene blue dye helps to improve the range for naked-eye detection of fluoride and arsenate with **C**_**1**_ and **C**_**2**_ probes. The addition of a preoptimized amount of methylene blue with the probe can detect up to 3 µM fluoride and 75 nM arsenate. The double logarithmic plot for both systems draws a straight line with slope (n) and log K_b_ as intercept as shown in Figs. S31b, 32b, depicting substantial binding for both the systems. Moreover, The **C**_**1**_ and **C**_**2**_ probes were utilized for the determination of unknown concentrations of fluoride and arsenate in tap water, lake water and seawater (Fig. S33, Table S5). A comparison table for fluoride and arsenic detection with earlier reported literature is given in Table 1. We have compared various parameters including detection method, solvent, LOD, recyclability and recovery of anions. To the best of our knowledge, oxacalixarene-based dual readout sensors have been reported for the first time for the detection of fluoride and arsenic in aqueous medium. Moreover, from the table, it is very clear that the sensors developed in this work possess excellent sensitivity, recyclability and anion recovery compared to previously reported sensors for fluoride and arsenic.

**Table 1:** Comparison between some recently reported probes and their sensitivity towards fluoride and arsenate.

Probe	Method	Solvent	Anions detected		LOD	Detection time	Recyclability	Recovery of anions (%)	References
			F^-^	AsO_4_^3−^					
Cobalt oxyhydroxide nanozyme	Colorimetric, electrochemical	Water	No	Yes	3.72 ppb	–	–	95–104	^38^
EDOT-functionalized Calix[4]pyrrole	Electrochemical	Water	Yes	No	0.19 ppm	–	–	–	^39^
ZnFe_2_O_4_ microspheres ionic liquid	Electrochemical	Water	No	Yes	0.00092 ppb	–	–	–	^40^
Luminescent lanthanide MOF	Luminescence	Water	Yes	No	0.1 ppm	5 min	Yes	–	^41^
MOF	Fluorescence	Water	No	Yes	15.7 ppb	10 min	Yes	–	^42^
Supramolecular lanthanide dimers	Fluorescence	Water	Yes	No	0.74 ppb	–	–	–	^43^
NiCo_2_O_4_-x-NH_2_ modified electrode	Low-pulse-energy LIBS	Water	No	Yes	8.69 ppb	10 min	–	96–104	^44^
Fe-GQDs	Fluorescence	Water	No	Yes	5.1 ppb	–	–	–	^45^
Zwitterionic spirocyclic metallogel	Fluorescence	Water	Yes	No	156 ppb	–	–	–	^46^
ArsenoFluor1	Fluorescence	THF	No	Yes	10 ppb	–	Yes	–	^47^
Oxacalix[4]arene	Fluorescence	Water	Yes	No	21 ppb	2 min	Yes	98–101	This work
			No	Yes	1.92 ppb	2 min	Yes	99–101	

### Density functional theory (DFT) studies

**Figure 7:**
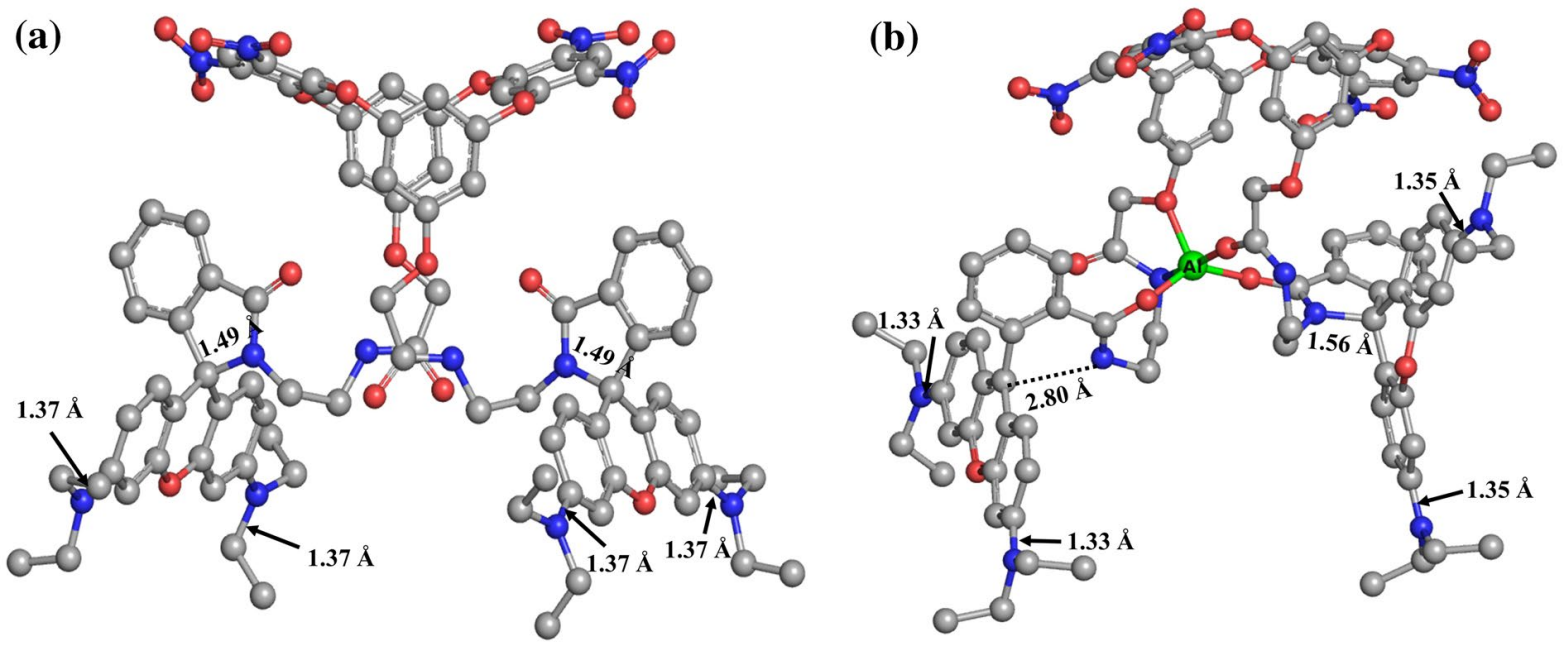
Ground state optimized geometries of (**a**) L and (**b**) L-Al^3+^ complex (Hydrogens are eliminated for better visualization).

The receptor L and its Al^3+^ complex were optimized at HF/3-21G* as shown in Fig. 7, and the corresponding geometrical parameters are summarized in Table S3. The computed result revealed Al^3+^ is pentacoordinated to **L** with O and N donor atoms where Al–N distance is 2.08 Å and Al–O distances are in the range of 1.72–1.88 Å. Some of the important bond distances are shown in Fig. 7. C–N bond distance of amino group decreased upon complexation from 1.37 to 1.33 Å, indicating a very minor contribution of charge transfer from amino group to xanthene. C–N bond of amide group increased from 1.49 to 2.80 Å indication ring-opening after complexation. The binding sites for both the trivalent metal ions Al^3+^ and Fe^3+^ are expected to be the same^48^. Therefore, we have performed the calculations for L-Al^3+^ as a representative complex.

**Figure 8:**
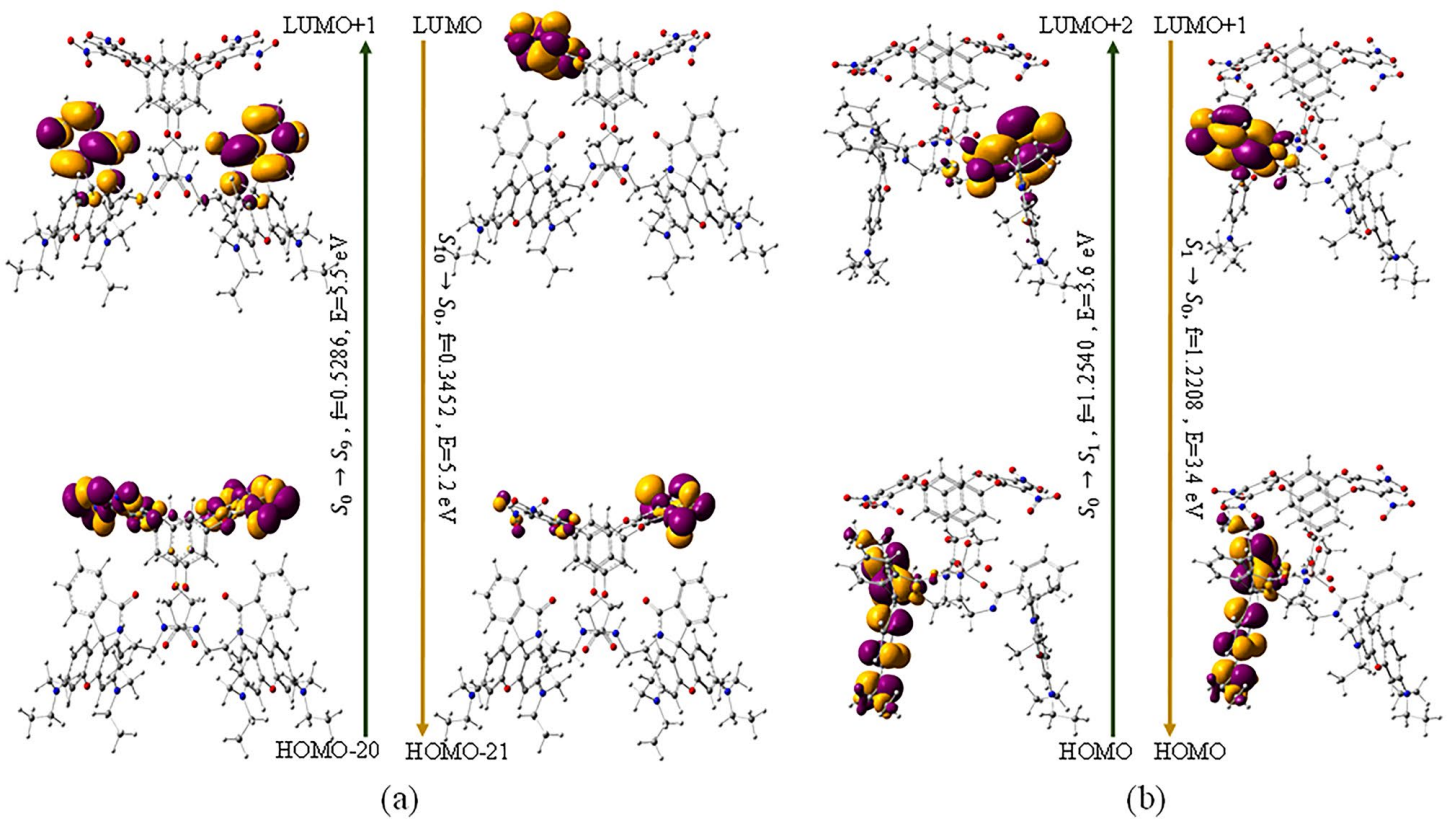
Electron density maps of the Frontier Molecular Orbitals (FMO) of L-Al^3+^ complex representing the electronic transitions of absorption and emission band on the optimized geometries of (**a**) L and (**b**) L-Al^3+^ at HF/3-21G*.

TD- DFT calculations at HF/3-21G* were performed for the lowest 10 singlet–singlet transitions. The computed absorption and emission energies, oscillatory strengths and corresponding wavelengths are summarized in Table S4 and the corresponding energy levels of FMOs associated with their HOMO and LUMO energy gaps are displayed in Fig. 8. The UV spectra generated by TD-DFT absorption method of L having oscillatory strength (a population density parameter f), 0.5285 related with the most important low-lying electronic transitions are HOMO–20 to LUMO + 1, HOMO–21 to LUMO, and HOMO–17 to LUMO. Emission for the same corresponding to LUMO to HOMO-21 with oscillatory strength 0.3452 has HOMO–LUMO gap (E_fund_) 2.48 eV. FMO shows there is no significant involvement of rhodamine B part in the fluorescence before ring opening. The L-Al^3+^ complex showed more intense absorption peak indicated with oscillatory strength 1.2540 associated with HOMO to LUMO + 2 transition (Fig. 8b). Emission spectra were also investigated with the same method on the optimized first excited states (S_1_) of L-Al^3+^. The calculated emission energies with their corresponding contributions are listed in Table S4. Emission of L-Al^3+^ complex with emission energy 3.4 eV corresponds to LUMO + 1 to HOMO electronic transitions with f = 1.2208 indicating the enhancement of the fluorescence intensity. This suggests the major contribution of π to π* transition in the xanthene is responsible for the enhanced fluorescence after the spiro-lactam ring-opening of the rhodamine B. The decreased HOMO–LUMO gap (E_fund_), 1.84 eV, in L-Al^3+^ suggests favourable complexation directing stable co-ordination. There are some reports where these excitation/emission energies vary when methods change from HF to B3LYP. It is seen that HF gives higher energy (lower wavelength) than the experimental results but the trend more or less remains the same^49^.

A molecular electrostatic potential (MESP) map of the receptor L has been plotted where the red region suggests the higher electron density whereas the blue region suggests the depletion of electron density as shown in Fig. S34. There are two main lower potential regions, one is around nitro groups on the calix ring and another is near the rhodamine B part. All nitro groups are involved in resonance with calix moiety, the only possible region where the metal can bind has shown with the red region near to rhodamine B part. This surface potential mapping gave us the idea of a plausible metal ion binding site.

### Fluorescent lifetime decay

Further, to inspect the change in fluorescence, we have conducted time-resolved decay measurements by following time-correlated single photon counting method. As mentioned earlier, the steady state fluorescent spectrum of ligand **L** exhibits a peak at 498 nm (λ_ex_ = 313). Upon addition of one equivalent of trivalent metal ions (Al^3+^ or Fe^3+^), an enhancement in emission intensity at 582 nm was observed with a simultaneous decrease in primary emission at 498 nm (Fig. S24). The fluorescence lifetime measurements for the ligand (**L**) and complexes (**C**_**1**_, **C**_**2**_) were conducted using 468 nm laser source and the variation in average fluorescence lifetime decay was recorded at both 498 and 582 nm. The average fluorescence lifetime values (τ_avg_) of the ligand (**L**) and corresponding complexes (**C**_**1**_ and **C**_**2**_) at 498 nm were determined to be 6.0, 5.6, and 7.8 ns respectively (Fig. S35, Table S6); whereas the same for 582 nm was calculated as 4.3, 2.5 and 2.7 ns (Fig. S36, Table S7). Additionally, the relative fluorescent quantum yield (Φ) for all the components including ligand (**L**) and complexes (**C**_**1**_ and **C**_**2**_) was determined by Eq. (3).

3 $${\Phi }_{\mathrm{S}} = {\Phi }_{\mathrm{ref}}\times \frac{1- {10}^{-O.D.(ref)}}{1- {10}^{-O.D.(S)}}\times \frac{A (S)}{A (ref)}\times \frac{{\eta }_{S}^{2}}{{\eta }_{ref}^{2}}.$$

Where the subscripts ref and S indicate reference and test samples respectively, Φ is the fluorescence quantum yield, A denotes the integrated fluorescence intensity, O.D. indicates the absorbance at the exciting wavelength for both molecules, $\eta$ is the refractive index of the solvents used. The relative fluorescence quantum yield of the compounds was measured by using Quinine Sulphate in 0.5 M H_2_SO_4_ (Φ = 0.546) as a reference dye. The relative fluorescent quantum yield (Φ in %) for L and C_1_ and C_2_ are estimated to be 0.017, 0.92, and 1.15, respectively.

### Sensing mechanism

**Figure 9:**
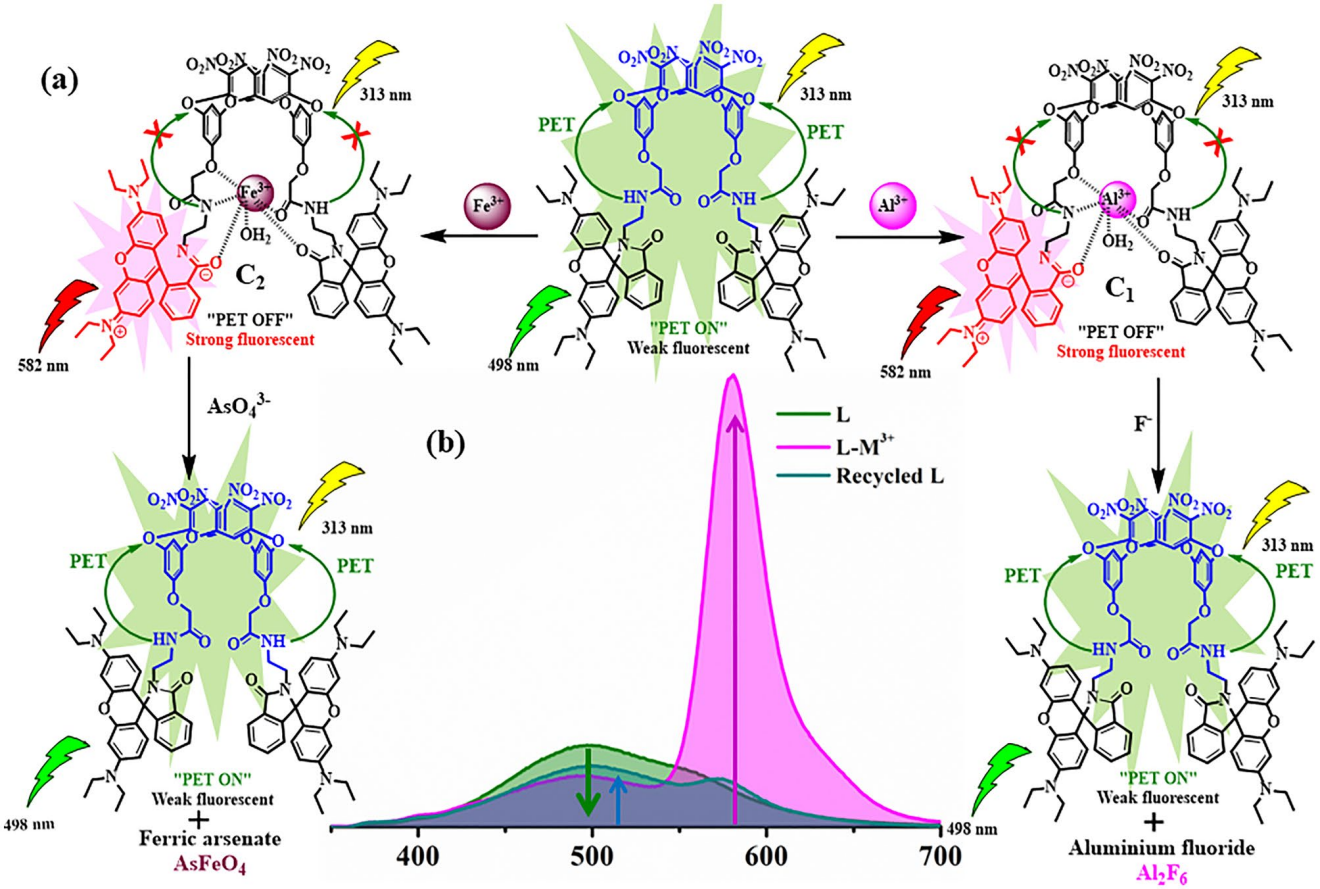
Plausible sensing mechanism of L toward Al^3+^**/**Fe^3+^, C_1_ toward Fluoride and C_2_ toward AsO_4_^3−^.

Herein*, bis*-(*N-*(rhodamine-B)lactam)oxacalix[4]arene (**L**) is established as a fluorescence “OFF–ON” system upon interaction with Al^3+^ and Fe^3+^. Both Al^3+^ and Fe^3+^ metals are efficiently involved in the ring-opening of spirolactam unit (Fig. 9). The receptor L exhibits a weak fluorescence at 498 may be due to the PET process from the N-donor site to the oxacalix[4]arene moiety^50,51^. Introduction of trivalent metal ions (M^3+^** = **Al^3+^/Fe^3+^) to the receptor **L**, viz. **C**_**1**_ (L-Al^3+^) and **C**_**2**_ (L-Fe^3+^), lead to inhibition of PET. As a result, the emission maxima (λ_max_ = 498 nm) of the receptor **L**, (characteristic emission due to PET) quenched and a new emission band at 582 nm appears may be due to spirolactam ring-opening (Fig. S24). This was further supported by DFT calculation and FTIR spectroscopy. DFT calculations provide structural evidence towards switching-ON the probe upon the addition of Al^3+^. The geometry optimized structure of 1:1 **L-Al**^**3+**^ complex displays that Al^3+^ mainly coordinates with the ‘N’ and ‘O’ site of the receptor followed by the partial ring-opening of spirolactam unit. The comparison between the FTIR spectra of **L** and **L + M**^**3+**^ indicates that the characteristic signal of the carbonyl group at 1696 cm^−1^, shifted towards lower wavenumber (1679 cm^−1^) upon interaction with trivalent metal ions (**M**^**3+**^), which recommends higher polarization of amide bond and simultaneous cleavage of spirolactam ring upon complexation (Fig. S37).

Later on, these **C**_**1**_ and **C**_**2**_ probes has been represented as “ON–OFF” systems upon interacting with fluoride and arsenate respectively (Fig. 9). In the presence of fluoride and arsenate, the emission maxima of **C**_**1**_ and **C**_**2**_ at 582 nm drastically quenches. Moreover, the pink color of the probes **C**_**1**_ and **C**_**2**_ vanished upon interaction with F^−^ and AsO_4_^3−^ respectively. This phenomenon may be attributed to the formation of aluminium fluoride^52,53^ and ferric arsenate^54,55^, which eventually leads to the regeneration of spirolactam rings followed by the abstraction of trivalent metal ions from the receptor (**L)** binding sites. The PXRD patterns of C_1_-F^−^ and C_2_-AsO_4_^3−^ further confirm the presence of aluminium fluoride and ferric arsenate respectively (Fig. S38). The suppression of emission intensity at 582 nm eventually concludes the restoration of PET “ON” process due to fluorescence ‘Turn-Off’.

### Recyclability

**Figure 10:**
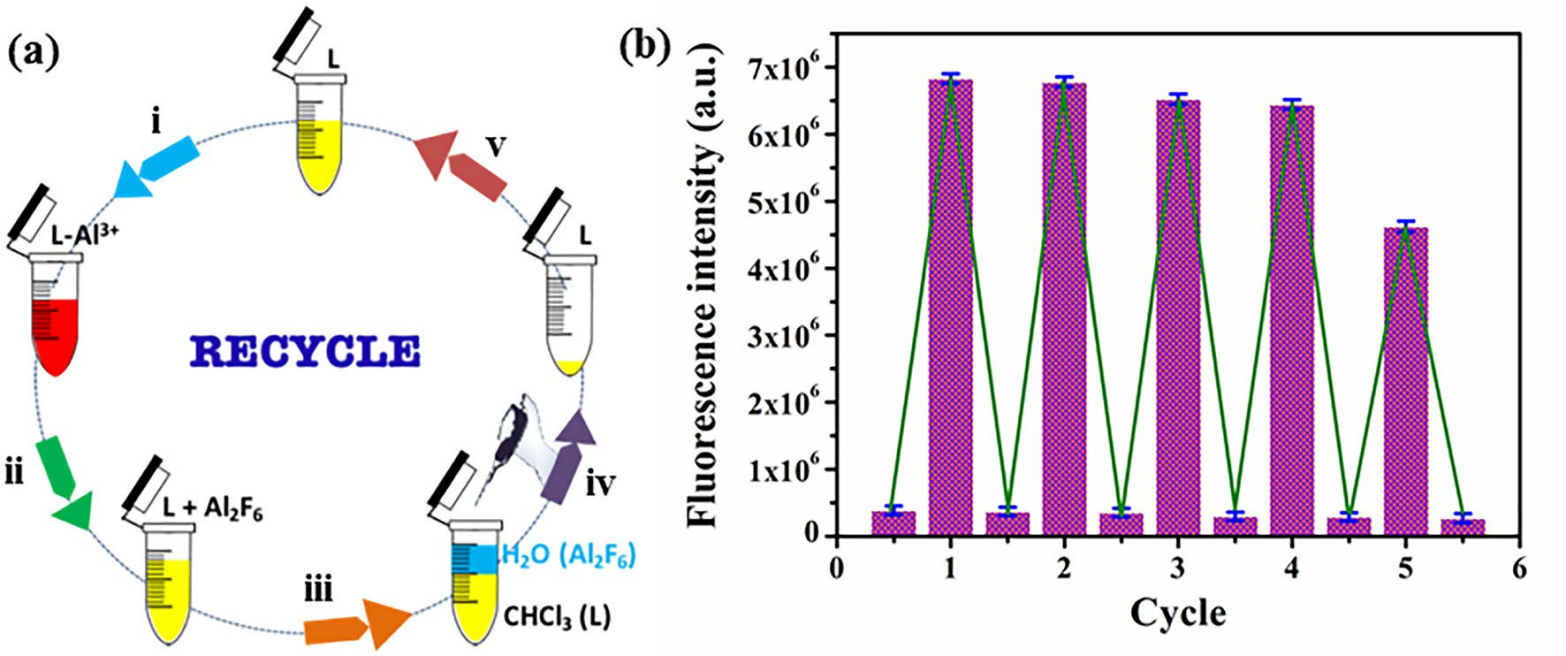
(**a**) Schematic representation of the recycle experiment (i) addition of Al^3+^ to the receptor L, (ii) addition of fluoride, (iii) extraction of organic layer with chloroform, (iv) removal of aqueous layer and drying of organic layer, (v) dissolve the crude in acetonitrile; (**b**) Recyclability of the probe L toward fluoride.

The recovery of a sensor is an important concern for its practical applications. The probe **C**_**1**_ exclusively interacts with fluoride anion in aqueous medium with excellent non-interference ability towards co-existent ions. Such high selectivity was attributed to the more nucleophilic nature of fluoride than other anions, which facilitated the breakdown of probe **C**_**1**_. Such disruption of the probe **C**_**1**_ into **L** and aluminium fluoride salts can easily be separated by organic solvents using layer separation methods, enabling the recycling process as shown in Fig. 10a. The receptor **L** was then recovered using chloroform mediated layer separation method. The top layer (aqueous) containing Na_3_AlF_6_ salts was discarded and the bottom layer containing yellow liquid of receptor **L** was collected for reprocessing. Surprisingly, no change in the color, as well as the photochemical properties of the recovered receptor was observed. The sensing ability of **L** barely deteriorated after 4 cycles of regeneration, indicating its potency of repeat usage in the application as shown in Fig. 10b. Similarly, C_2_ can also be utilized as a recyclable (up to 4 cycles) probe for arsenate.

### Fluoride detection using C_1_-based LFA

**Figure 11:**
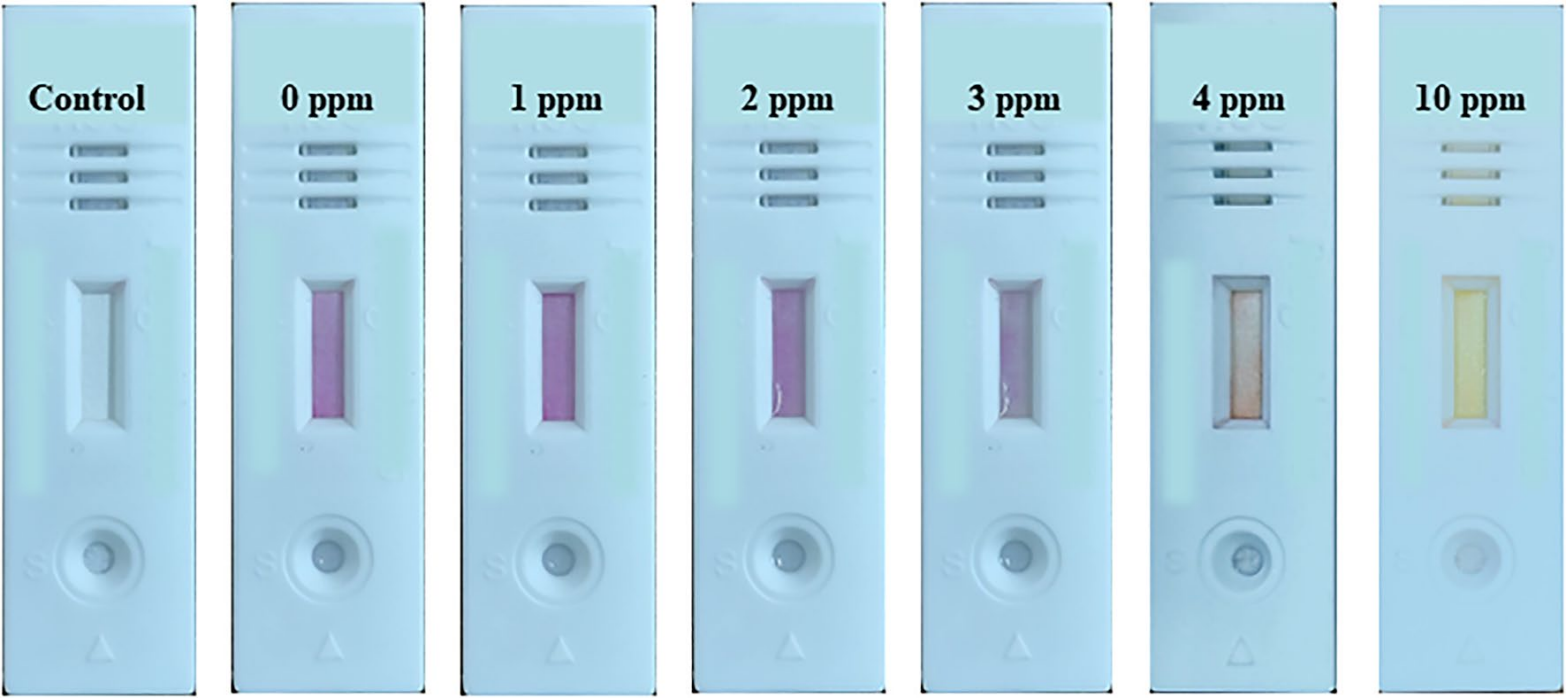
C_1_-based LFA for detection of fluoride in aqueous media.

A lateral flow assay-based paper analytical device (PAD) was fabricated for the on-spot detection of fluoride ions in aqueous medium. This PAD enables visual detection of the presence or absence of fluoride ions using a rapid and simple detection device. A schematic representation of the LFA-based PAD has been portrayed in Fig. 1b. When fluoride contaminated solution was added dropwise to the sample pad, the fluoride ions flowed toward the test zone and bound to the Al^3+^ site onto the **C**_**1**_ probe, allowing the formation of aluminium fluoride salts and free receptor (**L**) on the test zone, and the detection can be made based on color change. As the probe **C**_**1**_ doesn’t interact with the competitive anions; therefore, negative test results were observed in the test zone for the rest of the anions. More importantly, the addition of ≤ 3 ppm fluoride can’t be able to change the color of the test zone (Fig. 11). The non-captured/excess **C**_**1**_ continued to migrate toward the absorption pad (AP), where they interacted by slandered fluoride ions. Therefore, we observed a yellow colour test zone when the fluoride ions were detected, whereas a pinkish-red colour test zone was observed in the absence or ≤ 3 ppm of the fluoride.

### Detection of fluoride and arsenate in mammalian cells

**Figure 12:**
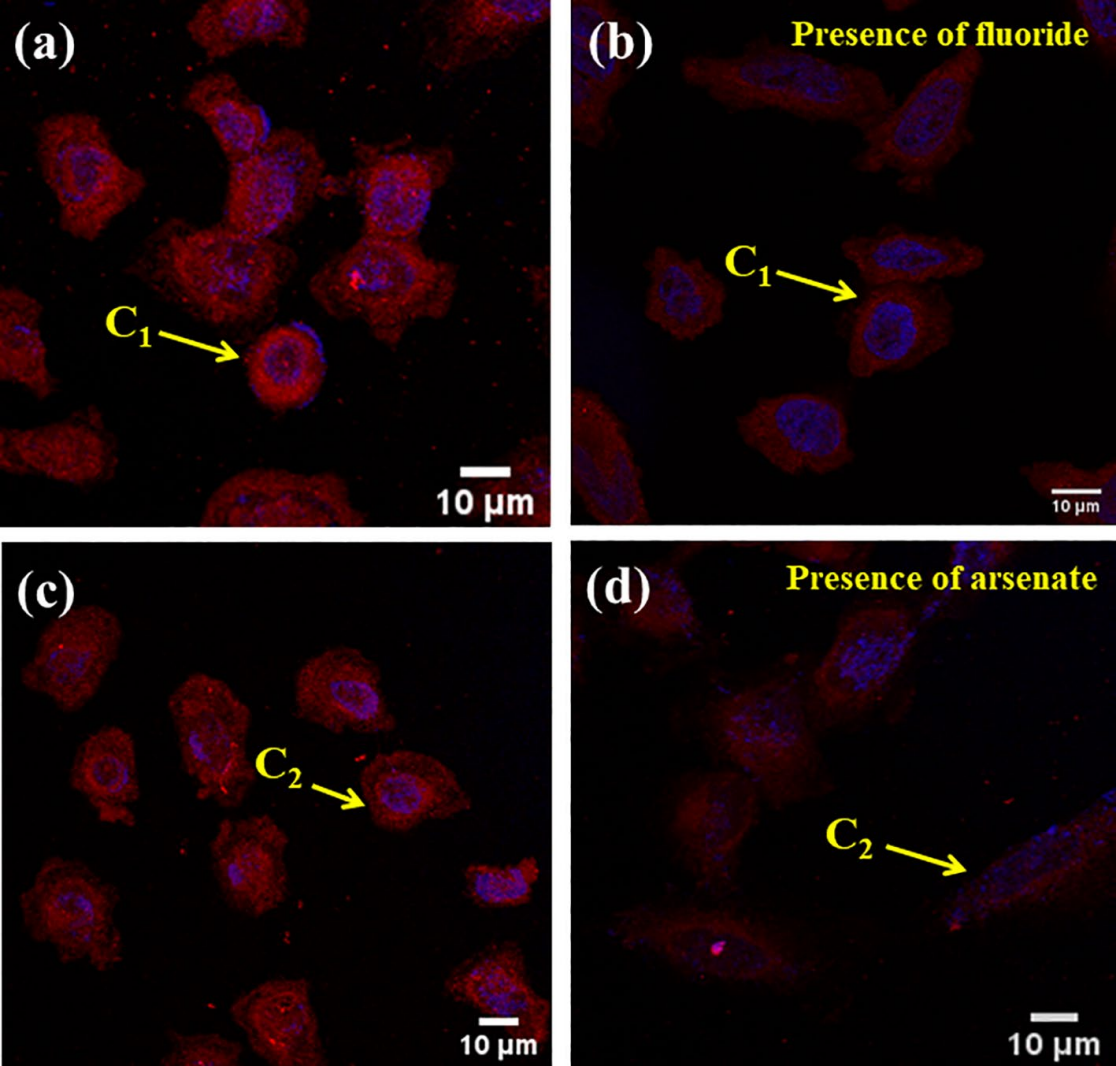
LSCM fluorescence imaging of SUM159 cell line with (**a**) C_1_, (**b**) C_1_ + F^−^, (**c**) C_2_, (**d**) C_2_-AsO_4_^3−^.

In order to investigate the efficiency of the receptor **L, C**_**1**_ and** C**_**2**_ in a living system, fixed cell imaging experiments were carried out using SUM159 triple-negative breast cancer cell line. For this purpose initially, the SUM159 cells were incubated with the receptor L with a concentration of 10 μM. Figure S39a shows that after incubation of 15 min, the cells were found to be weakly fluorescent, implying that the receptor can pass through cell membranes and then arrive at cells. After adding 10 μM of Fe^3+^/Al^3+^ solution, an apparent enhancement in fluorescence intensity was observed for both cases (Fig. S39b,c). Further, 10 μM **C**_**1**_ (L + Al^3+^) and 10 μM **C**_**2**_ (L + AsO_4_^3−^) systems were employed for the detection of fluoride and arsenate in SUM159 cell. As shown in Fig. 12a–d; a significant quenching in fluorescence signal was observed upon successive addition of 10 μM fluoride and 10 μM arsenate respectively. The variation in fluorescence intensities has been plotted as shown in Fig. S40. The outcomes further confirm that the probes **C**_**1**_ and **C**_**2**_ act as fluorescent sensors for the detection of arsenate and fluoride in living cells and aqueous media.

## Conclusions

We present a rhodamine B functionalized oxacalix[4]arene architecture as an ‘On–Off’ fluorescent probe for the sequential recognition of fluoride and arsenate in aqueous medium. The receptor **L** was initially involved in complexation with Al^3+^ and Fe^3+^ and the sensing of fluoride and arsenate was further achieved by the displacement of metals from the receptor binding sites. Both the anions display distinct color changes from pinkish red to pale yellow with significant quenching in emission maxima. Detailed emission titration studies indicate that the oxacalix[4]arene based receptor can efficiently detect fluoride and arsenate up to 21 ppb and 1.92 ppb, respectively, which is much lower than the corresponding permissible limits in drinking water by EPA. Moreover, the sensing of anions in aqueous solution via metal displacement process offers an alternate route for those systems, where one-to-one detection of anions will not be possible due to greater hydration energies of the counter system. To ensure the consumption of safe drinking water, we have used **C**_**1**_ embedded LFA to develop PoC device for the detection of aqueous fluoride with distinct colour changes from pink to yellow at the test zone of LFA. To the best of our knowledge, these probes (C_1_ and C_2_) are the first oxacalixarene-based chromogenic probes that were utilized as dual responsive, low-cost, recyclable, environment friendly, and convenient onsite detection of fluoride and arsenate. Additionally, this **C**_**1**_ based simple LFA kit provides a reliable assessment of fluoride ions in complex environmental samples. Our future target is to develop LFA based device with multi detection channel for the detection of environmental contaminants below standard levels to ensure safe drinking water. Therefore, the development of this oxacalixarene-based dual readout sensor will certainly create a significant impact in the area of supramolecular chemistry and anion recognition as well.

## Supplementary Information

Supplementary Video 1.

Supplementary Information 1.

Supplementary Information 2.

Supplementary Information 3.

Supplementary Video 2.

## Data Availability

The authors confirm that the data supporting the findings of this study are available in its supplementary material. CCDC number: 1985477.
