# Supplementary material for: Ultrasensitive colorimetric detection of fluoride and arsenate in water and mammalian cells using recyclable metal oxacalixarene probe: a lateral flow assay
Source: Sci Rep. 2022 Oct 12;12:17119. doi: 10.1038/s41598-022-21407-w (PMC9556598; doi:10.1038/s41598-022-21407-w)

checkCIF/PLATON report

Structure factors have been supplied for datablock(s) Compound1

THIS REPORT IS FOR GUIDANCE ONLY. IF USED AS PART OF A REVIEW PROCEDURE FOR PUBLICATION, IT SHOULD NOT REPLACE THE EXPERTISE OF AN EXPERIENCED CRYSTALLOGRAPHIC REFEREE.

No syntax errors found. CIF dictionary Interpreting this report

Datablock: Compound1

```
Bond precision:  C-C = 0.0083 A          Wavelength=0.71073

Cell:          a=14.3488(9)      b=15.0188(9)     c=15.2848(10)
               alpha=97.162(5)   beta=92.241(4)   gamma=101.857(4)
Temperature:   140 K
```

	Calculated	Reported
Volume	3191.4(4)	3191.4(4)
Space group	P -1	P -1
Hall group	-P 1	-P 1
Moiety formula	C24 H12 N4 O14, C H4 O [+ solvent]	2(C24 H12 N4 O14), 2(C H4 O)
Sum formula	C25 H16 N4 O15 [+ solvent]	C50 H32 N8 O30
Mr	612.42	1224.83
Dx,g cm-3	1.275	1.275
Z	4	2
Mu (mm-1)	0.108	0.108
F000	1256.0	1256.0
F000’	1256.86	
h,k,lmax	17,17,18	17,17,18
Nref	11231	11054
Tmin,Tmax	0.974,0.992	0.643,0.745
Tmin’	0.968	

```
Correction method= # Reported T Limits: Tmin=0.643 Tmax=0.745
AbsCorr = MULTI-SCAN

Data completeness= 0.984          Theta(max)= 25.000

R(reflections)= 0.1041( 6294)     wR2(reflections)= 0.3288( 11054)

S = 1.036                  Npar= 799
```

The following ALERTS were generated. Each ALERT has the format
test-name_ALERT_alert-type_alert-level.
Click on the hyperlinks for more details of the test.

Alert level B

```
PLAT230_ALERT_2_B Hirshfeld Test Diff for    O27      --N8       .        9.8 s.u.
PLAT420_ALERT_2_B D-H Bond Without Acceptor  O19      --H19      .     Please Check
PLAT420_ALERT_2_B D-H Bond Without Acceptor  O24      --H24A     .     Please Check
PLAT420_ALERT_2_B D-H Bond Without Acceptor  O33      --H33A     .     Please Check
```

Alert level C

```
DIFMX02_ALERT_1_C  The maximum difference density is > 0.1*ZMAX*0.75
            The relevant atom site should be identified.
PLAT084_ALERT_3_C High wR2 Value (i.e. > 0.25) ...................       0.33 Report
PLAT097_ALERT_2_C Large Reported Max.  (Positive) Residual Density       0.70 eA-3
PLAT220_ALERT_2_C NonSolvent   Resd 2  O   Ueq(max)/Ueq(min) Range        3.3 Ratio
PLAT230_ALERT_2_C Hirshfeld Test Diff for    O28      --N8       .        5.8 s.u.
PLAT241_ALERT_2_C High   'MainMol' Ueq as Compared to Neighbors of         O2 Check
PLAT241_ALERT_2_C High   'MainMol' Ueq as Compared to Neighbors of        O18 Check
PLAT242_ALERT_2_C Low    'MainMol' Ueq as Compared to Neighbors of         N1 Check
PLAT242_ALERT_2_C Low    'MainMol' Ueq as Compared to Neighbors of         N4 Check
PLAT242_ALERT_2_C Low    'MainMol' Ueq as Compared to Neighbors of         N5 Check
PLAT242_ALERT_2_C Low    'MainMol' Ueq as Compared to Neighbors of         N8 Check
PLAT260_ALERT_2_C Large Average Ueq of Residue Including      O32      0.102 Check
PLAT334_ALERT_2_C Small Aver. Benzene C-C Dist C25      -C30            1.37 Ang.
PLAT340_ALERT_3_C Low Bond Precision on  C-C Bonds ...............   0.00829 Ang.
PLAT415_ALERT_2_C Short Inter D-H..H-X       H19      ..H26      .       2.10 Ang.
                                                 -x,1-y,2-z  =      2_567 Check
PLAT790_ALERT_4_C Centre of Gravity not Within Unit Cell: Resd.  #          1 Note
              C24 H12 N4 O14
PLAT906_ALERT_3_C Large K Value in the Analysis of Variance ......      4.477 Check
PLAT906_ALERT_3_C Large K Value in the Analysis of Variance ......      2.332 Check
PLAT911_ALERT_3_C Missing FCF Refl Between Thmin & STh/L=    0.595        174 Report
```

Alert level G

```
PLAT007_ALERT_5_G Number of Unrefined Donor-H Atoms ..............          6 Report
PLAT042_ALERT_1_G Calc. and Reported Moiety Formula Strings Differ     Please Check
PLAT045_ALERT_1_G Calculated and Reported Z Differ by a Factor ...       2.00 Check
PLAT072_ALERT_2_G SHELXL First  Parameter in WGHT  Unusually Large       0.19 Report
PLAT432_ALERT_2_G Short Inter X...Y Contact  C21      ..C26              3.17 Ang.
                                                 -x,1-y,1-z  =      2_566 Check
PLAT606_ALERT_4_G Solvent Accessible VOID(S) in Structure ........          ! Info
PLAT720_ALERT_4_G Number of Unusual/Non-Standard Labels ..........          1 Note
PLAT790_ALERT_4_G Centre of Gravity not Within Unit Cell: Resd.  #          3 Note
              C H4 O
PLAT910_ALERT_3_G Missing # of FCF Reflection(s) Below Theta(Min).          3 Note
PLAT933_ALERT_2_G Number of OMIT Records in Embedded .res File ...         23 Note
PLAT941_ALERT_3_G Average HKL Measurement Multiplicity ...........        2.7 Low
PLAT978_ALERT_2_G Number C-C Bonds with Positive Residual Density.          0 Info
PLAT992_ALERT_5_G Repd & Actual _reflns_number_gt Values Differ by          2 Check
```

0 **ALERT level A** = Most likely a serious problem - resolve or explain
4 **ALERT level B** = A potentially serious problem, consider carefully
19 **ALERT level C** = Check. Ensure it is not caused by an omission or oversight
13 **ALERT level G** = General information/check it is not something unexpected

3 ALERT type 1 CIF construction/syntax error, inconsistent or missing data

```
20 ALERT type 2 Indicator that the structure model may be wrong or deficient
 7 ALERT type 3 Indicator that the structure quality may be low
 4 ALERT type 4 Improvement, methodology, query or suggestion
 2 ALERT type 5 Informative message, check
```

It is advisable to attempt to resolve as many as possible of the alerts in all categories. Often the minor alerts point to easily fixed oversights, errors and omissions in your CIF or refinement strategy, so attention to these fine details can be worthwhile. In order to resolve some of the more serious problems it may be necessary to carry out additional measurements or structure refinements. However, the purpose of your study may justify the reported deviations and the more serious of these should normally be commented upon in the discussion or experimental section of a paper or in the "special_details" fields of the CIF. checkCIF was carefully designed to identify outliers and unusual parameters, but every test has its limitations and alerts that are not important in a particular case may appear. Conversely, the absence of alerts does not guarantee there are no aspects of the results needing attention. It is up to the individual to critically assess their own results and, if necessary, seek expert advice.

Publication of your CIF in IUCr journals

A basic structural check has been run on your CIF. These basic checks will be run on all CIFs submitted for publication in IUCr journals (*Acta Crystallographica*, *Journal of Applied Crystallography*, *Journal of Synchrotron Radiation*); however, if you intend to submit to *Acta Crystallographica Section C* or *E* or *IUCrData*, you should make sure that full publication checks are run on the final version of your CIF prior to submission.

Publication of your CIF in other journals

Please refer to the *Notes for Authors* of the relevant journal for any special instructions relating to CIF submission.

PLATON version of 03/06/2021; check.def file version of 02/06/2021

Datablock Compound1 - ellipsoid plot

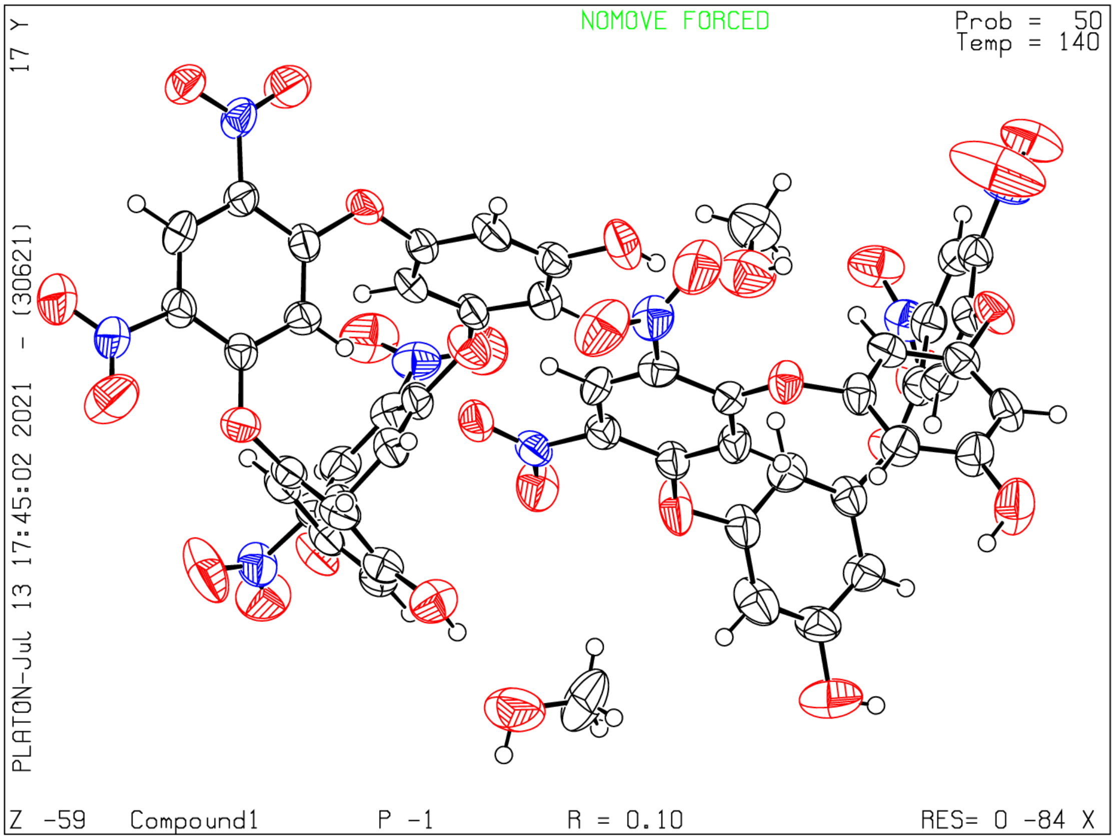

Supplement: Supplementary file 2 — Supplementary Information 1. [file 41598_2022_21407_MOESM2_ESM.pdf]
