# Supplementary material for: Ultrasensitive colorimetric detection of fluoride and arsenate in water and mammalian cells using recyclable metal oxacalixarene probe: a lateral flow assay
Source: Sci Rep. 2022 Oct 12;12:17119. doi: 10.1038/s41598-022-21407-w (PMC9556598; doi:10.1038/s41598-022-21407-w)
Supplement: Supplementary file 4 — Supplementary Information 3. [file 41598_2022_21407_MOESM4_ESM.docx]

*Electronic Supplementary Information for*

**Ultrasensitive colorimetric detection of fluoride and arsenate in water and mammalian cells using recyclable metal oxacalixarene probe: A lateral flow assay**

Shuvankar Dey^1,⊥^, Anshu Kumar^1,2,⊥^, Pradip Kumar Mondal^3,4^, Deepak Chopra^4^, Rupam Roy^4^, Sana Jindani^5^, Bishwajit Ganguly^5^, Chaithra Mayya^6^, Dhiraj Bhatia^6^, Vinod K. Jain^1^*

^1^ Department of Chemistry, School of Sciences, Gujarat University, Ahmedabad-380009, India

^2^ Department of Chemistry, University of Delhi, New Delhi 110007, India

^3^ Elettra - Sincrotrone Trieste, S.S. 14 Km 163.5 in Area Science Park, 34149 Basovizza Trieste, Italy

^4^ Department of Chemistry, Indian Institute of Science Education and Research Bhopal, Bhauri, Bhopal 462066, India

^5^ Computation and Simulation Unit (Analytical and Environmental Science Discipline and Centralized Instrument Facility), CSIR–Central Salt and Marine Chemicals Research Institute, Bhavnagar, Gujarat 364002, India

^6^ Biological Engineering Discipline, Indian Institute of Technology, Gandhinagar 382355, Gujarat, India

^⊥^ These authors contributed equally to this work

*Corresponding author: Vinod K. Jain; E-mail: [drvkjain@hotmail.com](mailto:drvkjain@hotmail.com), Tel.: +91-9327013263

**S1. Instruments**

Mass spectra were recorded on a Q-TOF MicroTM LC-MS instrument. FTIR analyses were performed with Bruker ALPHA – II FT-IR spectrometer. NMR experiments were conducted on a Bruker FT-NMR instrument, Avance II (600 MHz) at 298 K with TMS as the internal reference (Ettlingen, Germany). ^1^H NMR and ^13^C NMR were obtained at a base frequency of 600 & 150 MHz respectively. UV−vis absorption spectra were recorded on a Jasco V-750 spectrophotometer (Tokyo, Japan) with EHCS-760 ranging of 200–800 nm. The fluorescence spectra and relative fluorescence intensities were measured on a Horiba Fluoromax-4 (Germany). Inductively coupled plasma- optical emission spectroscopy ICP-OES (ICP, Perkin Elmer, Optima 2000) was used to standardize fluorescence calibration plot. The fixed cell imaging was carried out in Leica laser confocal microscope (model: TCS SP8).

**S2. Synthesis of Parent Oxacalix[4]arene (1)**

The parent oxacalix[4]arene was synthesized as per previously reported literature by our group.^1^ Typically, 1,5-difluoro-2,4-dinitrobenzene (0.98 mmol, 1 eqiv.), Phloroglucinol (0.98 mmol, 1 eqiv.) and finely ground K_2_CO_3_ (2.45 mmol, 2.5equiv) were allowed to stir at room temperature for 3 hours in 20 ml of DMSO. The progress of the reaction was monitored through TLC. After completion of the reaction, the aqueous layer was extracted with ethyl acetate (30 ml x 3). Then the organic layer was washed with brine solution, dried over anhydrous Na_2_SO_4_, filtered and concentrated in vacuo to get yellow solid of parent oxacalix[4]arene (Compound 1).

**MS (ESI):** m/z for C_24_H_12_N_4_O_14_ Calcd: 580.04 ([M] ^+^), Found 579.03 (Figure S1); **^1^H-NMR (500 MHz, DMSO *d_6_*)** δ = 10.53 (s 2H), 8.89 (s, 2H), 6.75 (s, 2H), 6.59 (s, 6H) (Figure S2). **^13^C NMR (125 MHz, DMSO *d_6_*)** δ = 160.50, 155.55, 154.60, 133.77, 124.75, 108.66, 105.21, 101.14 (Figure S3); **Elemental analysis:** Calcd for C_24_H_12_N_4_O_14:_ C 49.67, H 2.08, N 9.65; found C 49.71, H 2.11, N 9.59

**Scheme S1** Synthetic scheme of parent oxacalix[4]arene (1)

**S3. Synthesis of** **diethyl-(bis(oxy)diacetyl)oxacalix[4]arene (2)**

To a solution of compound 1 (1g, 1.7 mmol) in dry acetone (25 ml), K_2_CO_3_ (0.59 ml, 4.31 mmol) was added. Ethyl bromoacetate (0.48 ml, 4.31 mmol) was then added dropwise and the reaction mixture was allowed to reflux for 24 hours. The development of the product was monitored with the help of TLC. After completion of the reaction, the mixture was cooled to room temperature, neutralized and the aqueous layer was extracted with ethyl acetate (25 ml × 3). The organic layer was washed with brine solution, dried over anhydrous Na_2_SO_4_, filtered and concentrated in vacuo to get brown solid of the product. The crude was then recrystallized in methanol to obtain pure compound 2 with a yield of (1.05 g) 84.6%.

**MS (ESI):** m/z for C_32_H_24_N_4_O_18_ Calcd: 752.12 ([M] ^+^), Found 752.46; (Figure S4); **^1^H NMR (600 MHz, CDCl_3_)** δ = δ 8.79 (s 2H), 6.67 (s 4H), 6.38 (d 4H), 4.66 (t 4H), 4.27(s 4H), 1.31(t 6H), (Figure S5); **^13^C NMR (150 MHz, CDCl_3_)** δ = 167.63, 161.44, 155.74, 155.01, 133.20, 125.82, 106.53, 106.08, 105.00, 65.53, 61.86, 14.13 (Figure S6). **Elemental analysis:** Calcd. For C_32_H_24_N_4_O_18:_ C 51.07, H 3.21, N 7.45; found C 51.09, H 3.23, N 7.51

**Scheme S2** Synthetic scheme for the preparation of compound 2

**S4. Synthesis of** ***N*-(rhodamine-B)lactam-ethylenediamine (3)**

To a solution of rhodamine B (1 g, 2.08 mmol) in ethanol (50 ml), ethylenediamine (0.69 ml, 10.43 mmol) was added. The reaction mixture was then refluxed for 36 hours till the fluorescence of the solution disappeared. Then the mixture was cooled to room temperature, the orange precipitate obtained was collected and washed with cold ethanol. The crude product was then recrystallized with acetonitrile to purify the product with a yield of (0.895 g) 88.6 %. A schematic representation for the preparation of *N*-(rhodamine-B)lactam-ethylenediamine has been given as scheme S3.

**MS (ESI):** m/z for C_30_H_36_N_4_O_2_ Calcd: 484.28 ([M] ^+^), Found 484.0 (Figure S7); **^1^H NMR** **(600 MHz, CDCl_3_)** δ = 7.92 (q, 1H), 7.46 (q, 1H), 7.11, (q, 1H), 6.45 (s, 1H), 6.44 (s, 1H), 6.39 (d, 2H), 6.29 (dd, 2H), 3.34 (q, 8H), 3.20 (t, 2H), 2.42 (t, 2H), 1.61 (s, 1H), 1.18, (t, 12H) (Figure S8); **^13^C NMR** **(150 MHz, CDCl_3_)** δ = 168.66, 153.46, 148.80, 132.43, 131.20, 128.69, 128.06, 123.83, 122.76, 108.12, 105.59, 97.66, 64.94, 44.35, 43.81, 40.77, 12.59. (Figure S9); **Elemental analysis:** Calcd. For C_30_H_36_N_4_O_2:_ C 74.35, H 7.49, N 11.56; found C 74.39, H 7.42, N 11.61

**Scheme S3** Synthetic scheme for the preparation of compound 3

**S5. Characterization of *bis -*(*N*-(rhodamine-B)lactam)oxacalix[4]arene (L)**

**MS (ESI):** m/z for C_88_H_84_N_12_O_20_ Calcd: 1629.70 ([M] ^+^), Found 1630.61(Figure S10); **^1^H-NMR (600 MHz, CDCl_3_) δ (ppm):** 9.01 (s, 2H), 8.85 (s, 2H), 8.28 (s, 2H), 7.93 (s, 2H), 7.52-7.48 (d, 4H), 7.18 (d, 2H), 6.71-6.65 (dd, 4H), 6.48-6.21 (m, 14H), 4.76-4.51 (m, 4H), 4.32-4.21 (q, 4H), 3.43-3.22 (s, 16H), 3.11-2.97 (q, 4H), 1.25-1.07 (m, 24H) (Figure S11); **^13^C NMR (125 MHz, CDCl_3_):** δ 168.18, 167.58, 159.20, 157.91, 155.36, 153.56, 152.15, 147.95, 147.30, 132.26, 128.90, 128.03, 127.53, 127.49, 126.82, 125.01, 122.97, 121.88, 107.24, 103.15, 100.76, 99.39, 98.86, 97.84, 96.65, 64.52, 64.28, 60.46, 43.31, 43.24, 39.54, 37.30, 28.67, 13.12, 11.50, 11.48 (Figure S12); **Elemental analysis:** calcd. For C_88_H_84_N_12_O_20:_ C 64.86, H 5.20, N 10.31; found C 64.94, H 5.39, N 10.36

**S6. Characterization of L-Al^3+^ (C_1_) and L-Fe^3+^ (C_2_) complexes**

Complex **C_1_:** (L-Al^3+^) Yield: 55%, MS (ESI): m/z for C_88_H_85_AlN_12_O_21_ Calcd: 1672.5751, found 1672.5422 (Figure S13); FTIR: 3610 ν(-N-H) stretching, 3005-2939 νCH_2_ stretching, 2250 ν, 1635 ν(C=O), 1439, 1380, 1039, 920, 750 (Figure S14)

Complex **C_2_:** (L-Fe^3+^) Yield: 49%, MS (ESI): m/z for C_88_H_85_FeN_12_O_21_ Calcd: 1701.5291, found 1071.9611(Figure S15); FTIR: 3610 ν(-N-H) stretching, 3005-2939 νCH_2_ stretching, 2250 ν, 1635 ν(C=O), 1439, 1380, 1039, 920, 750 (Figure S16)

**S7. Optimization of reaction conditions**

The sensitivity of our designed receptor **L** can majorly be influenced by the factor incubation time. To ascertain its sensitivity and reproducibility, the absorption responses of **L** with different concentrations of Al^3+^ and Fe^3+^ were investigated at different time intervals. It was observed that 3 minutes of incubation time is enough with no noticeable change in ΔAbs_559_ afterwards (Figure S23). Therefore, the optimum conditions to carry out the experiments were set with 3 min incubation time. We have investigated the pH stability of the probes C_1_ and C_2_ within the range of 3.5 to 10.5. We observed a variation in fluorescence intensity in the lower pH values (pH<7). In higher pH, (pH>7) the emission intensity found to stable and no significant change in emission intensity was observed within the neutral to alkaline pH range (Figure S29). Therefore, we have decided to perform the experiments in neutral pH. All the experiments, including the biological studies were conducted in neutral pH (pH 7.4).

**S8. Determination of limit of detection (LOD):**

The LOD for fluoride and arsenic were determined by the equation LOD= 3.3σ/k, where σ represents the standard deviation of blank sample and k refers to the slope of calibration curve.


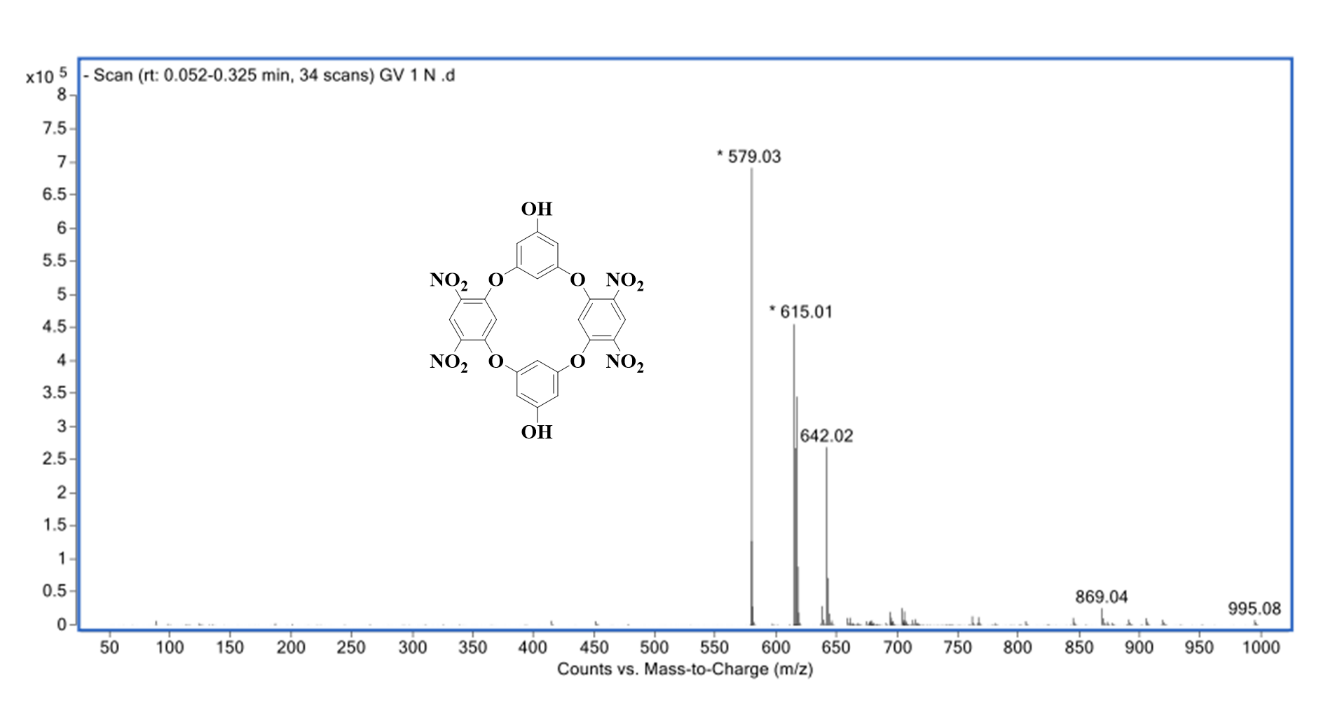


**Figure S1** ESI-MS spectrum of compound 1

**
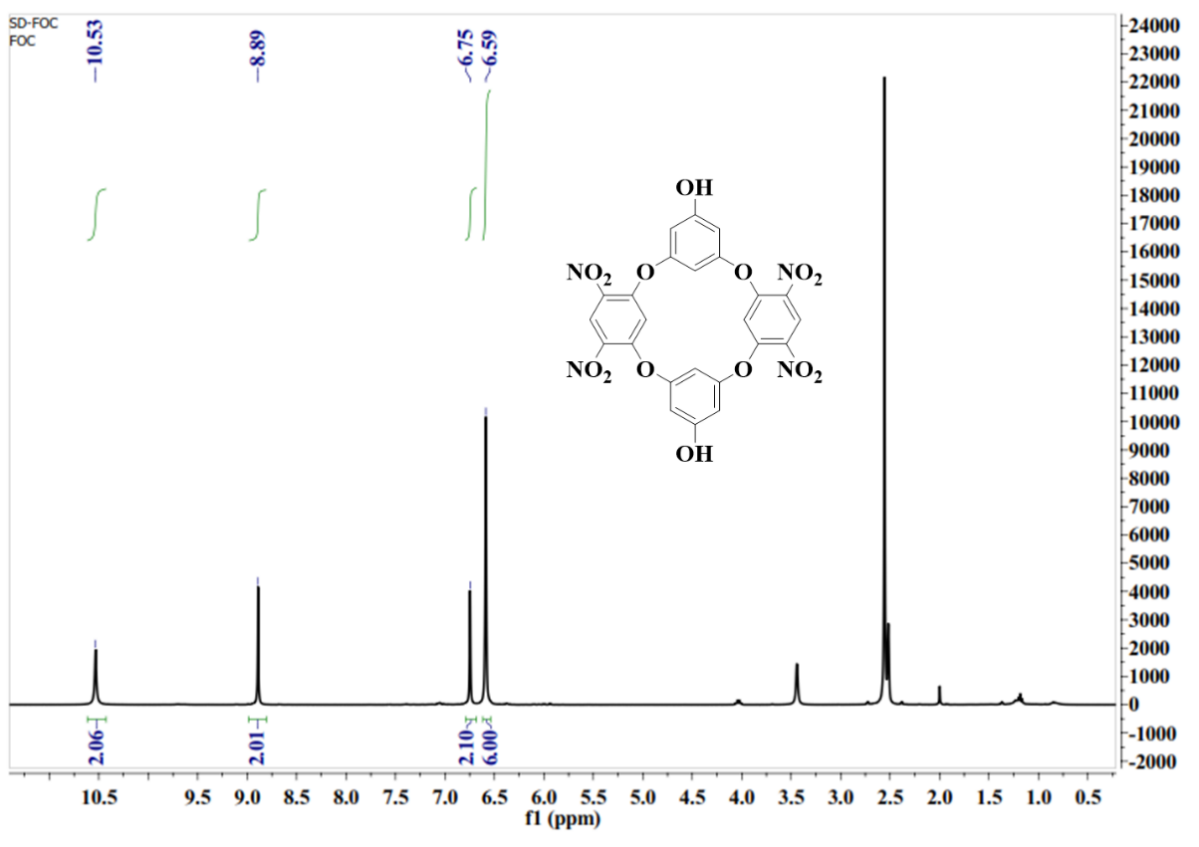
**

**Figure S2** ^1^H-NMR spectrum of compound 1


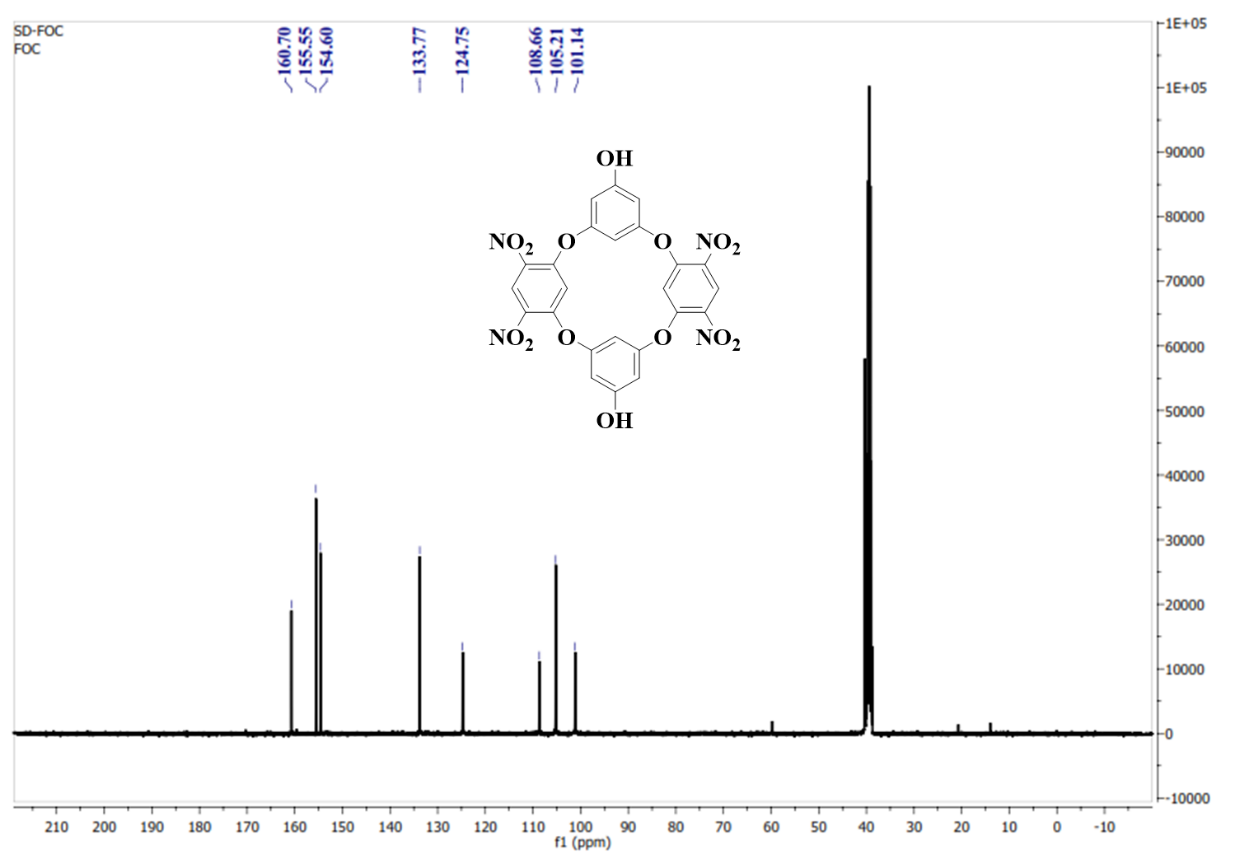


**Figure S3** ^13^C-NMR spectrum of compound 1





**Figure S4** ESI-MS spectra of compound 2


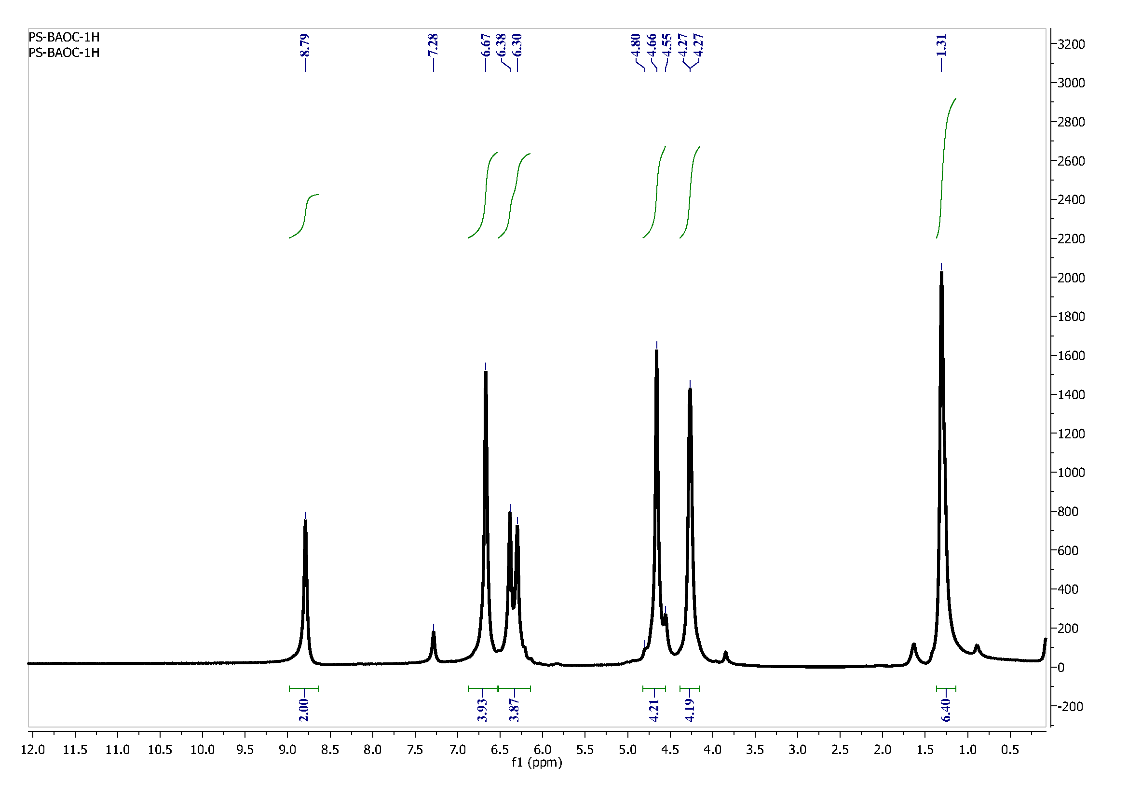


**Figure S5** 1H-NMR spectrum of compound 2


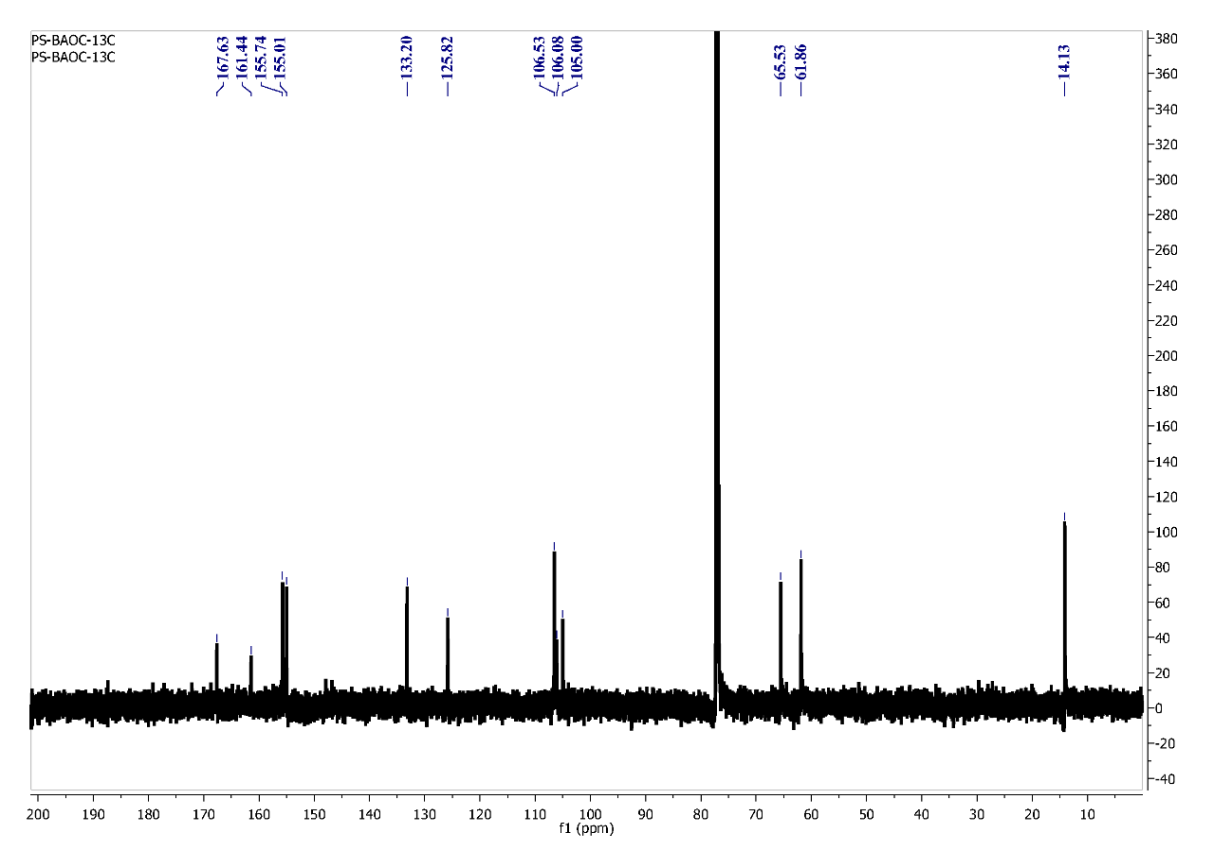


**Figure S6** ^13^C-NMR spectrum of compound 2


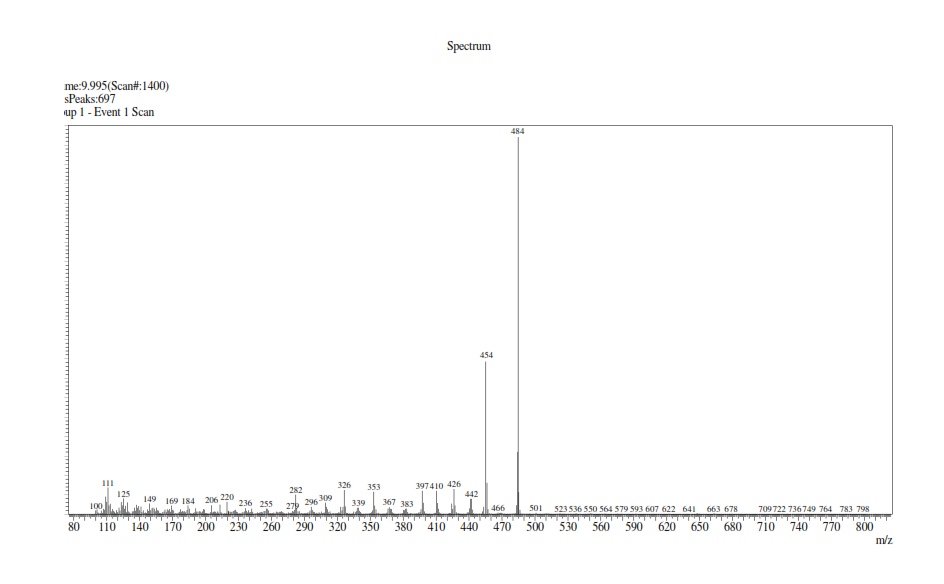


**Figure S7** ESI-MS spectrum of compound 3


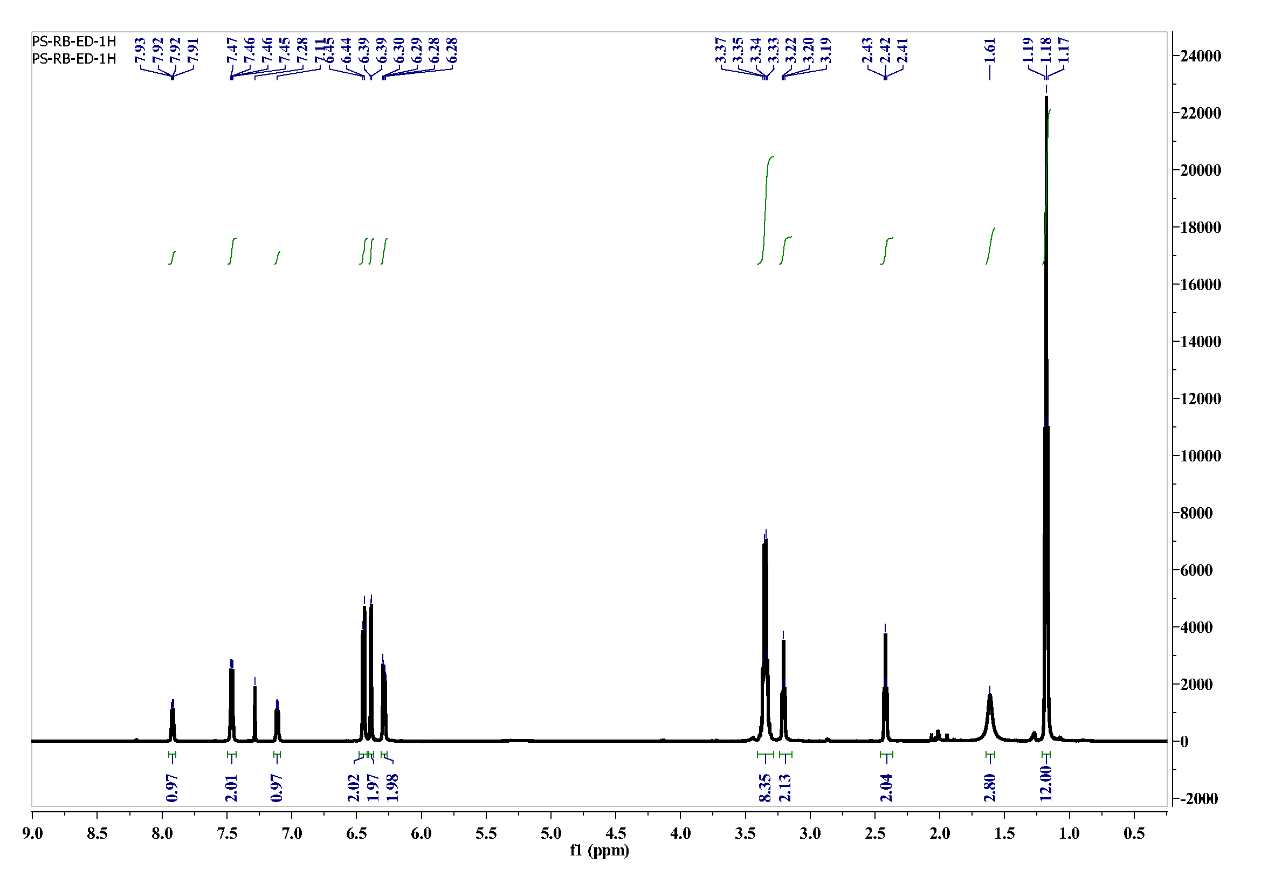


**Figure S8** ^1^H-NMR spectrum of compound 3


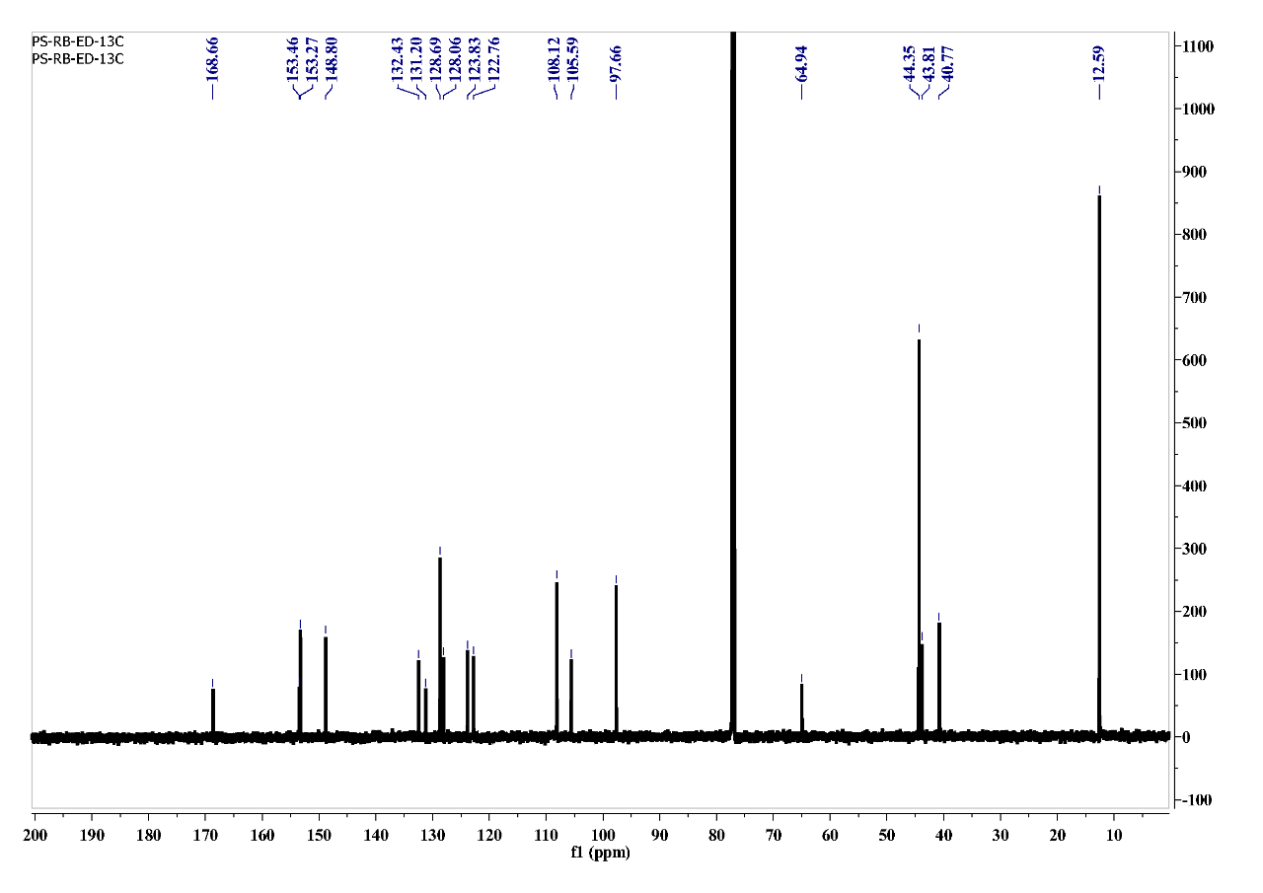


**Figure S9** ^13^C-NMR spectrum of compound 3


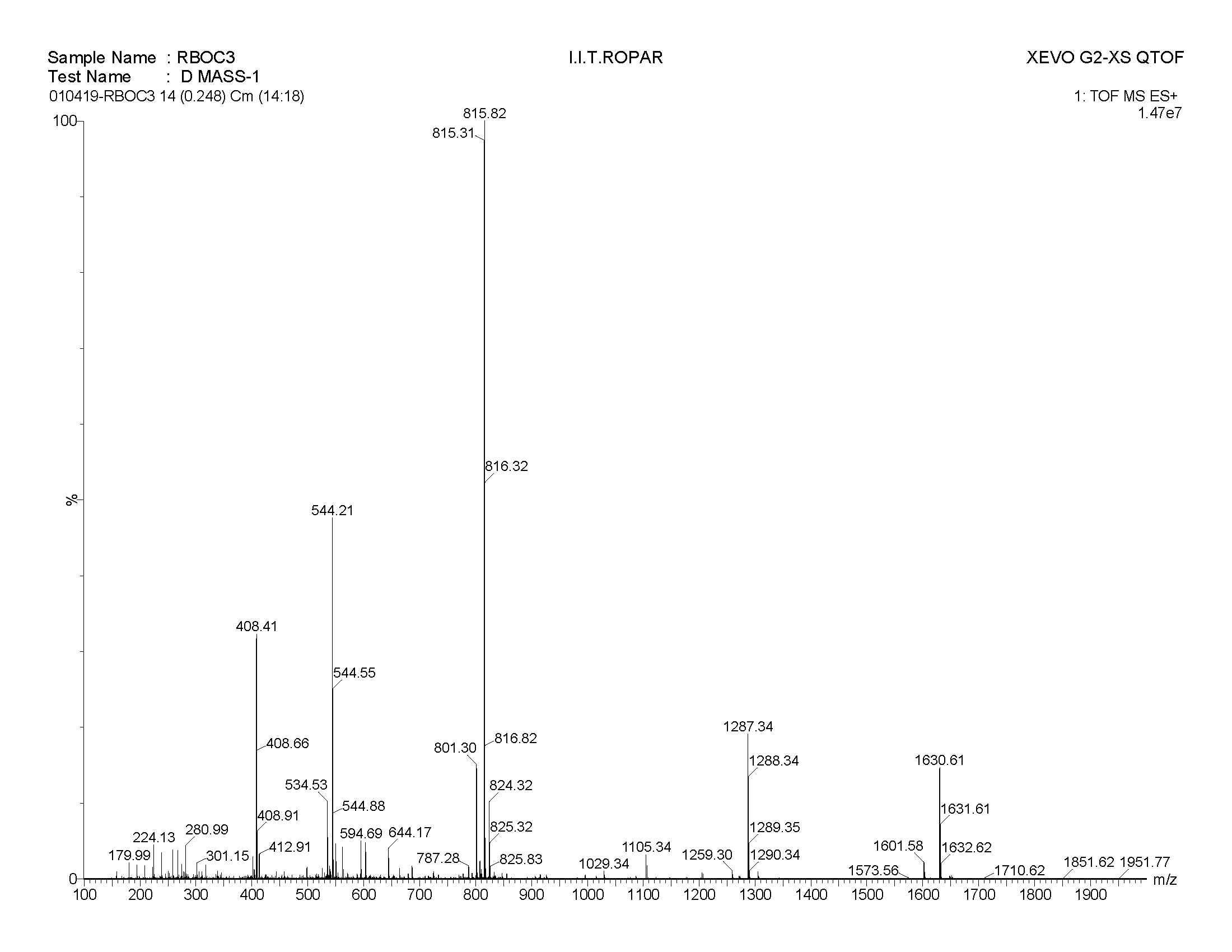


**Figure S10** ESI-MS spectrum of the receptor L.


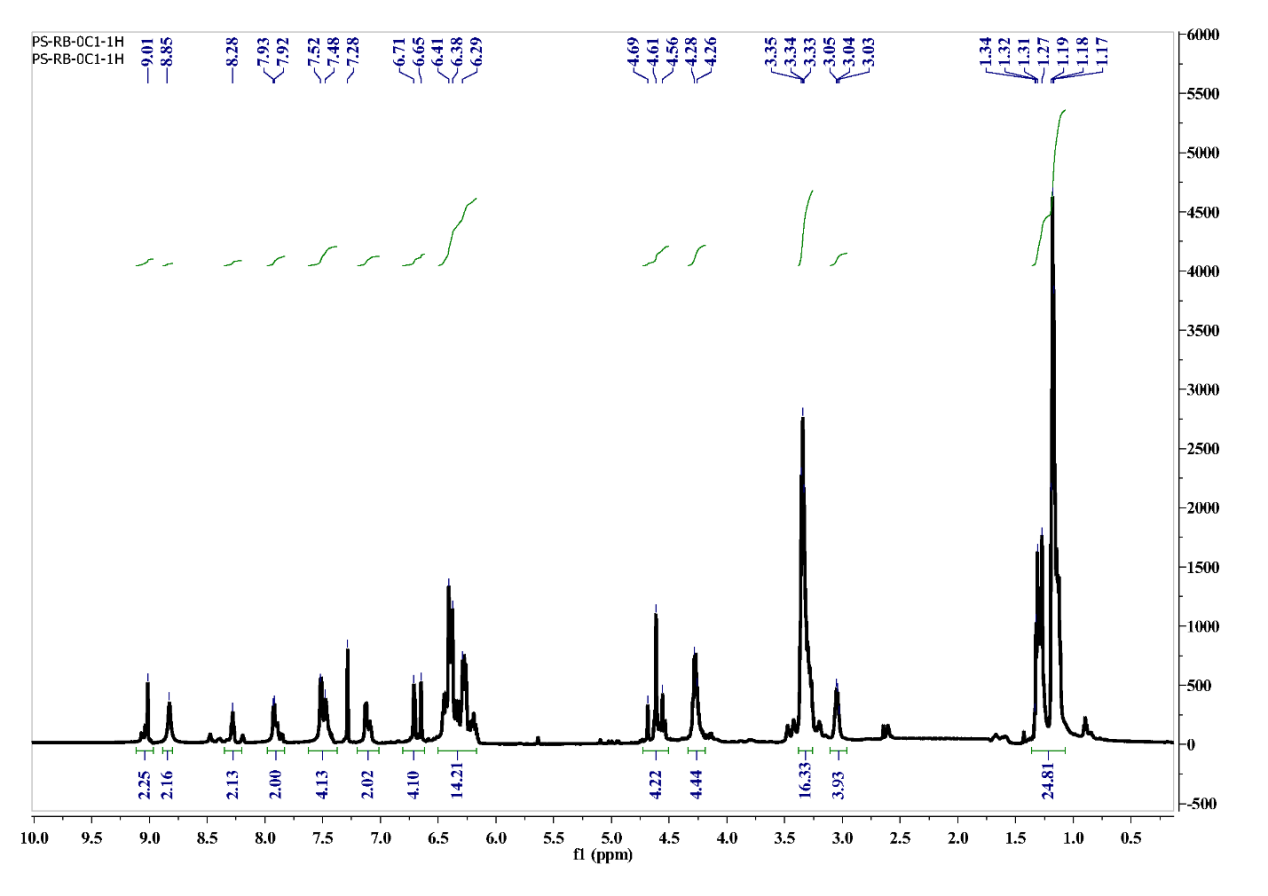


**Figure S11** ^1^H-NMR spectrum of receptor L.


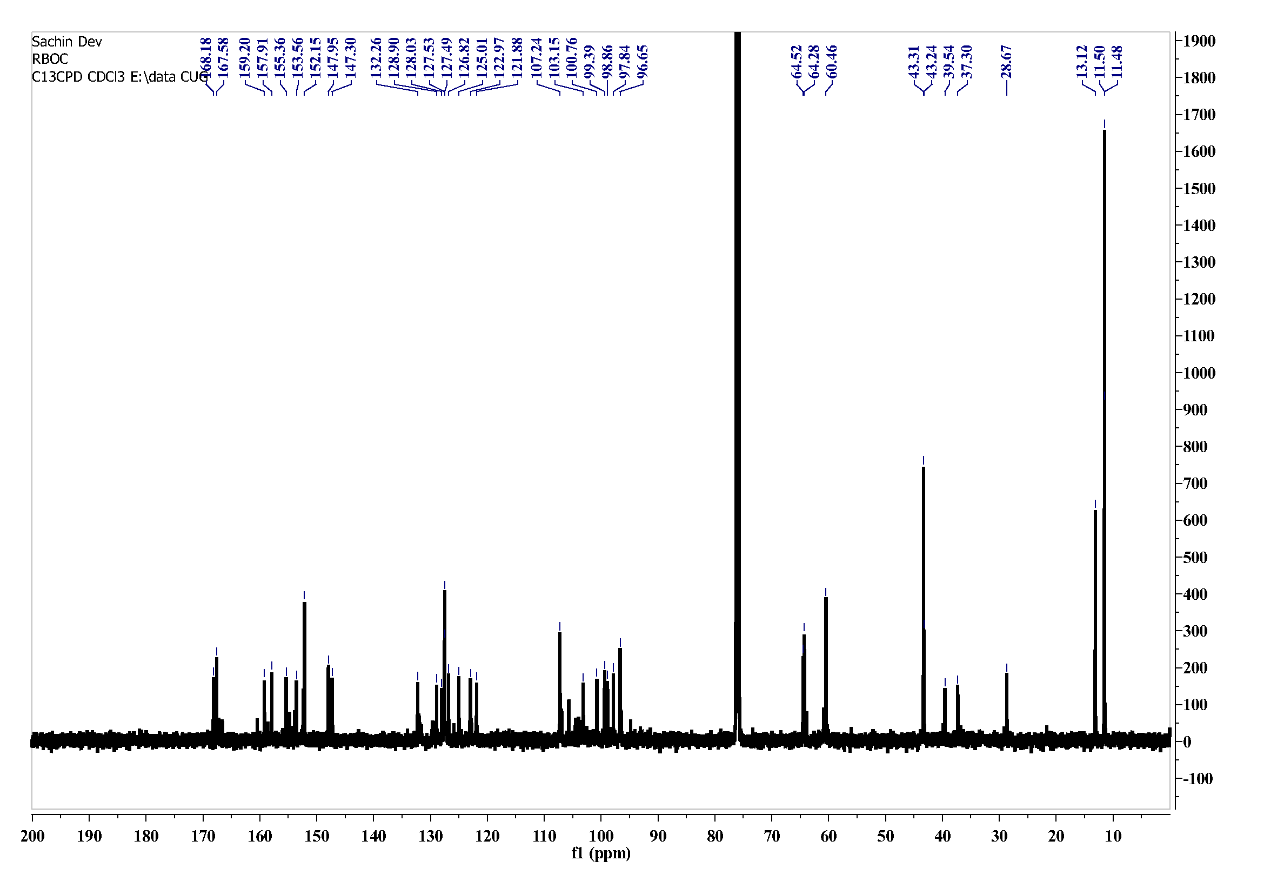


**Figure S12** ^13^C-NMR spectrum of the receptor L.


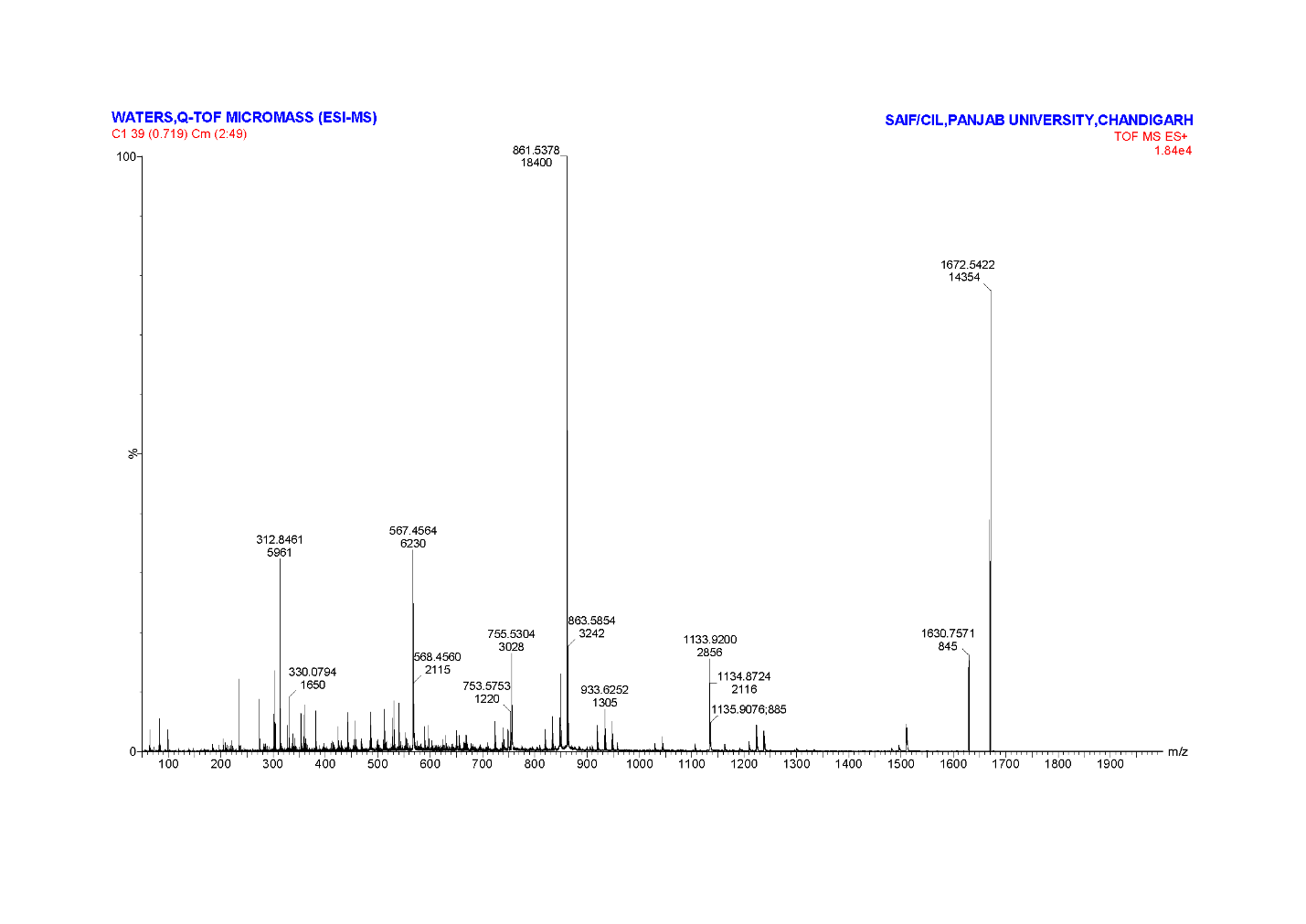


**Figure S13** ESI-MS spectrum of the complex C_1._





**Figure S14** Comparison FTIR spectrum of the receptor L (top) and C_1_ (bottom).


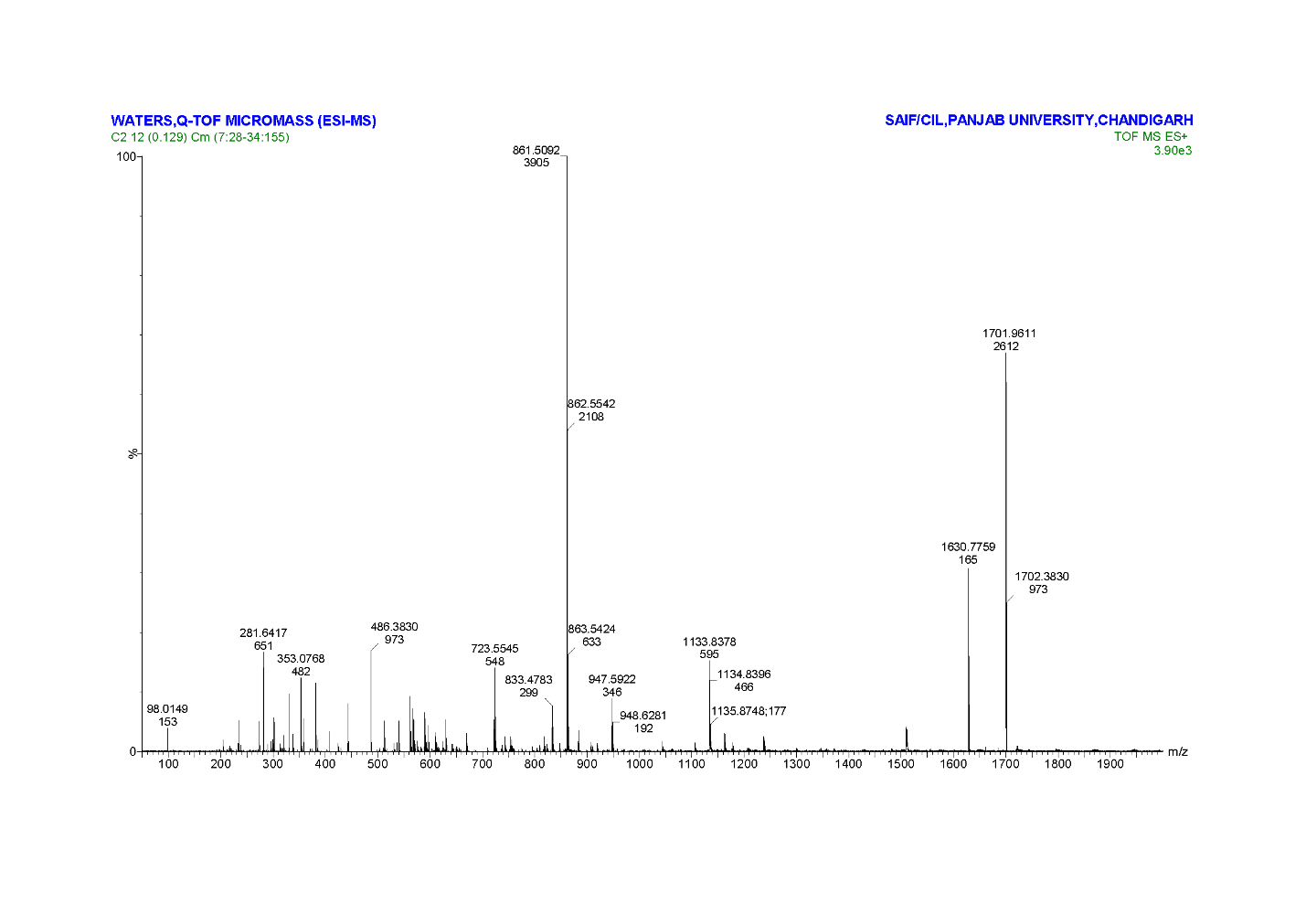


**Figure S15** ESI-MS spectrum of the complex C_2._





**Figure S16** Comparison FTIR spectrum of the receptor L (top) and C_2_ (bottom).


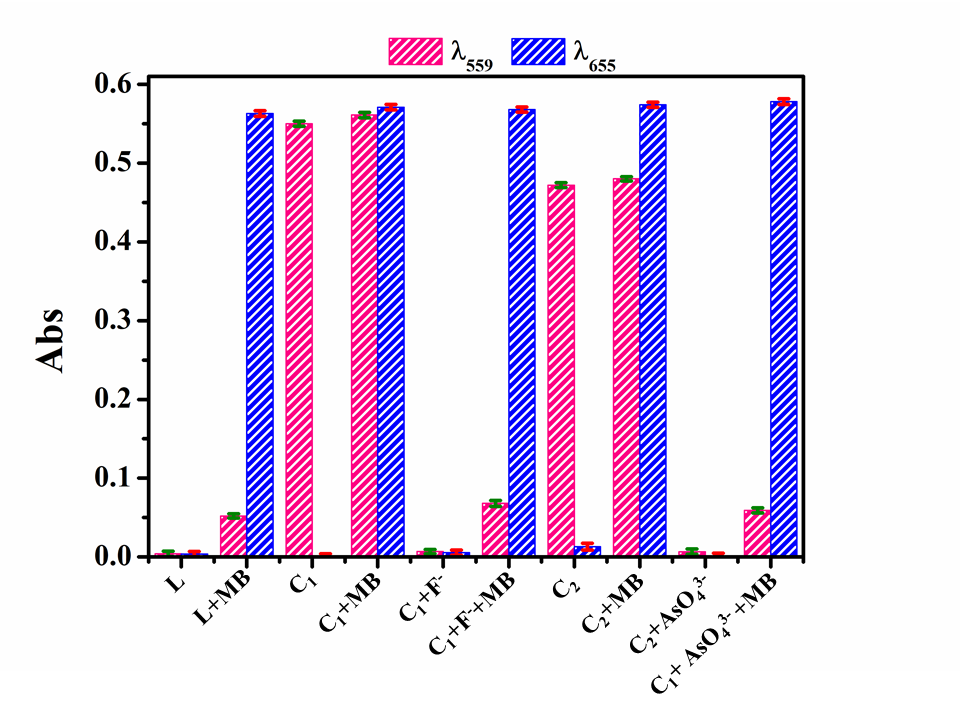


**Figure S17** Variation in absorption intensities of L, C_1_ and C_2_ upon addition of preoptimized methylene blue dye.


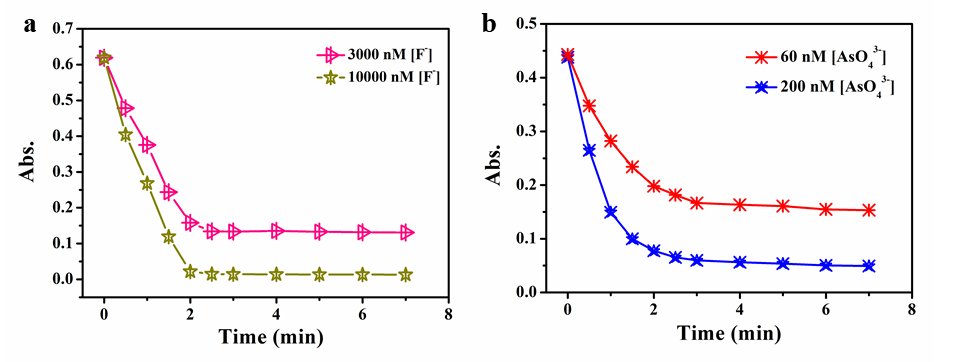


**Figure S18** Reaction kinetics between the probe C_1_–F^-^ and C_2_-AsO_4_^3-^ studied spectrophotometrically.


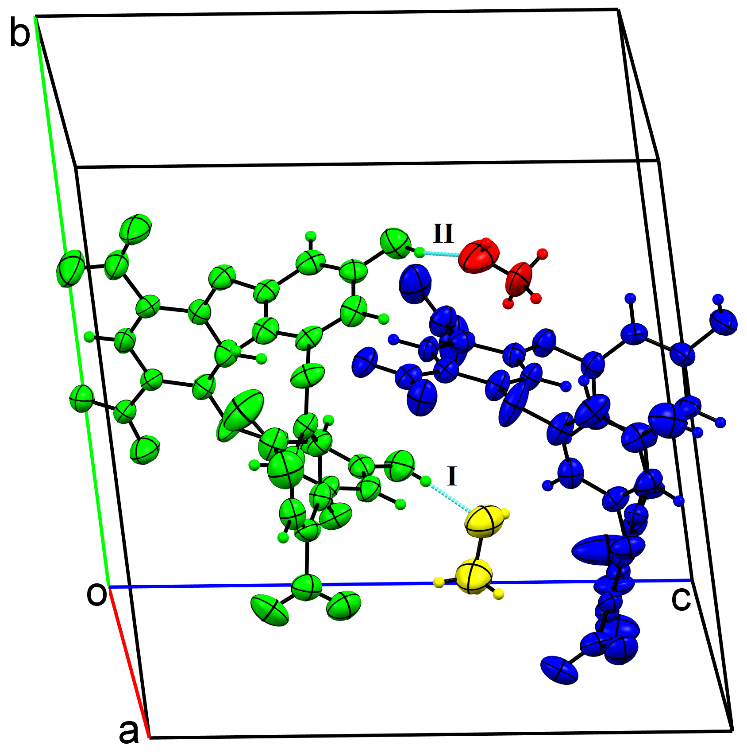


**Figure S19** ORTEP view of the asymmetric unit of compound **1** with different symmetry equivalence has been shown in different color codes (blue and green) and the MeOH molecule has been shown as red and yellow respectively. O-H…O H-bonded interaction involved within the asymmetric unit between compound 1 (green) and MeOH (yellow, red) has been represented as motif I and II respectively.


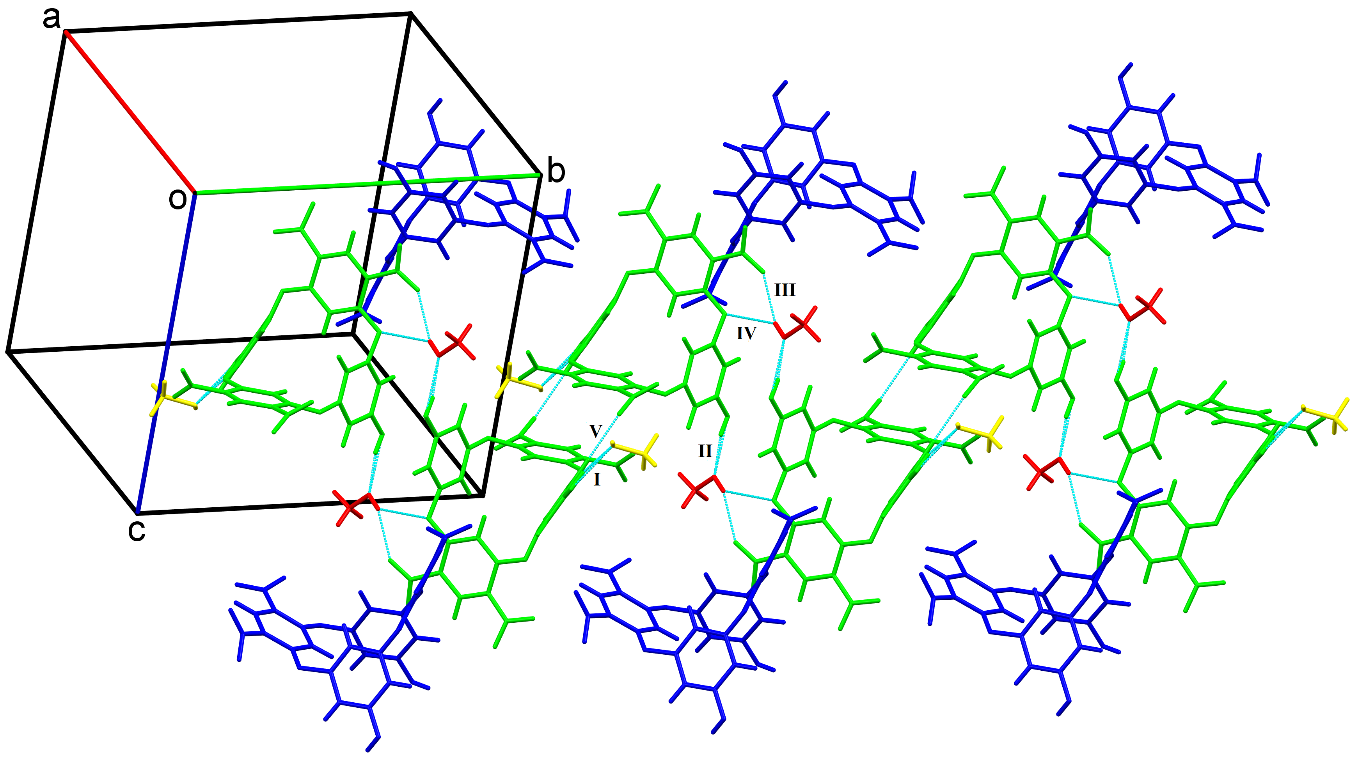


**Figure S20** Formation of chain like structure through the interconnection of compound **1** (green color) and MeOH (red color) molecules via motif I, II, III, IV and V.


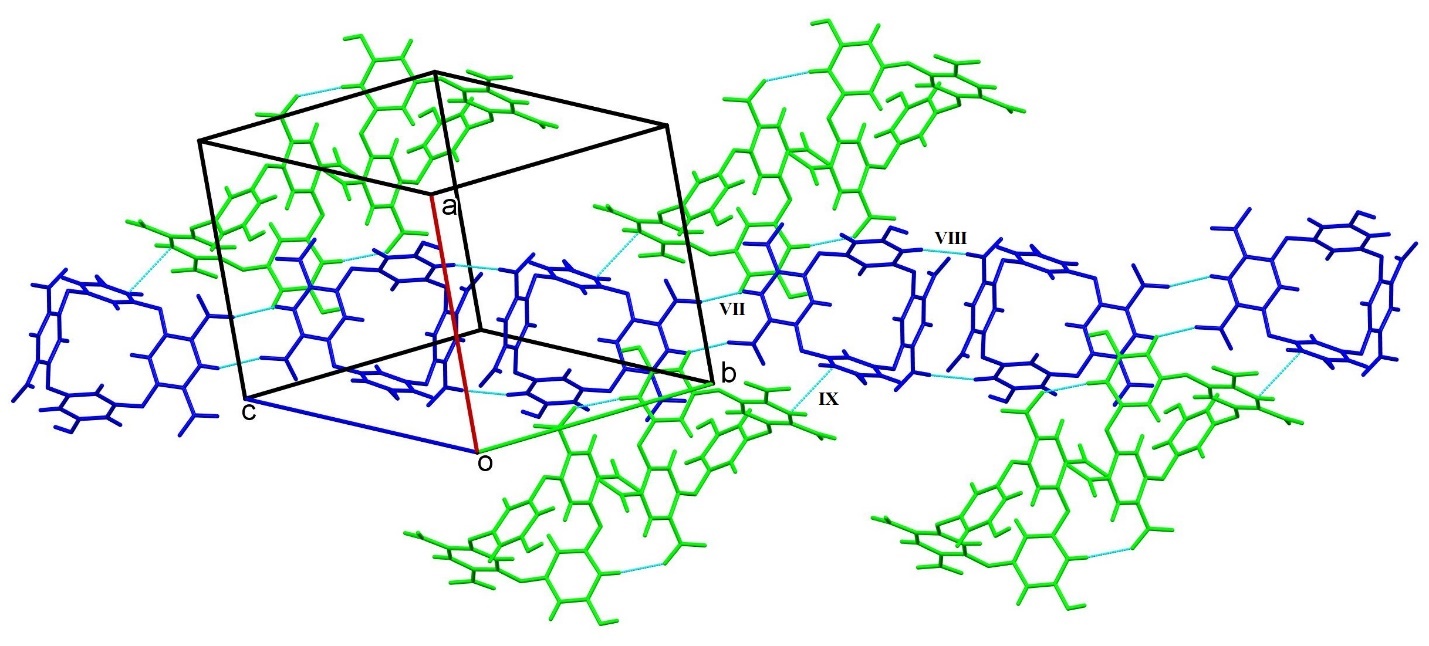


**Figure S21** Crystal packing depicting the interlinking of the molecules in compound **1** (blue color) via motif VII, VIII and IX.





**Figure S22** Spectral overlap between bis-(N-(rhodamine-B)lactam)oxacalix[4]arene (L) emission and ring opened rhodamine B absorption.


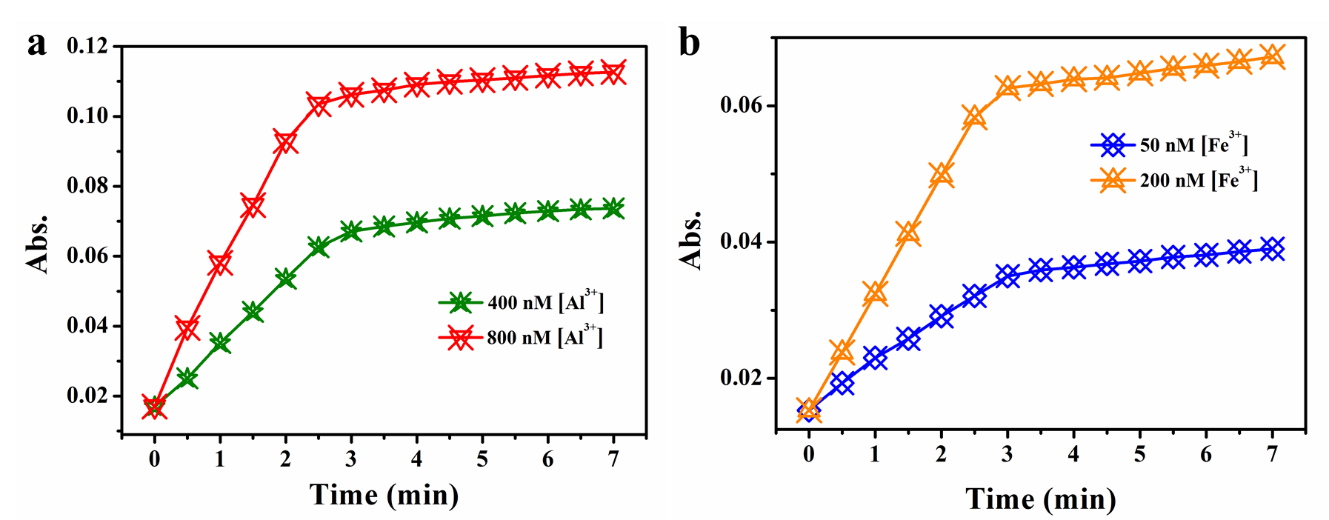


**Figure S23** Reaction kinetics between (a) L-Al^3+^ and (b) L-Fe^3+^ studied spectrophotometrically.


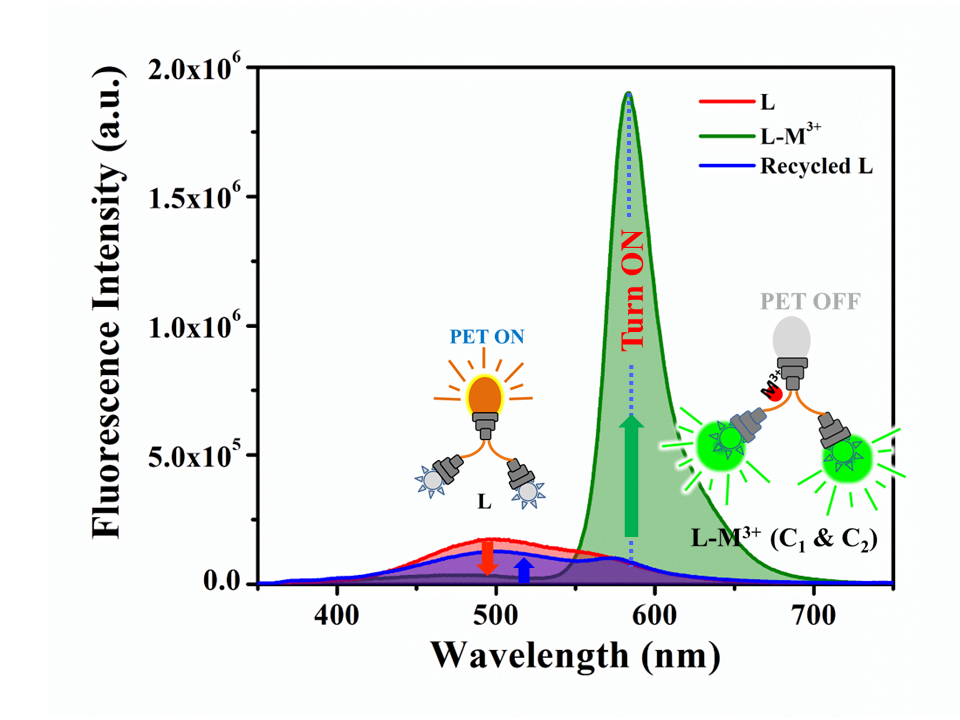


**Figure S24** Emission spectrum of the receptor L (red line, PET “ON”), metal complex (green line, Turn “ON” fluorescence) and recycled ligand (blue line, PET “ON”).


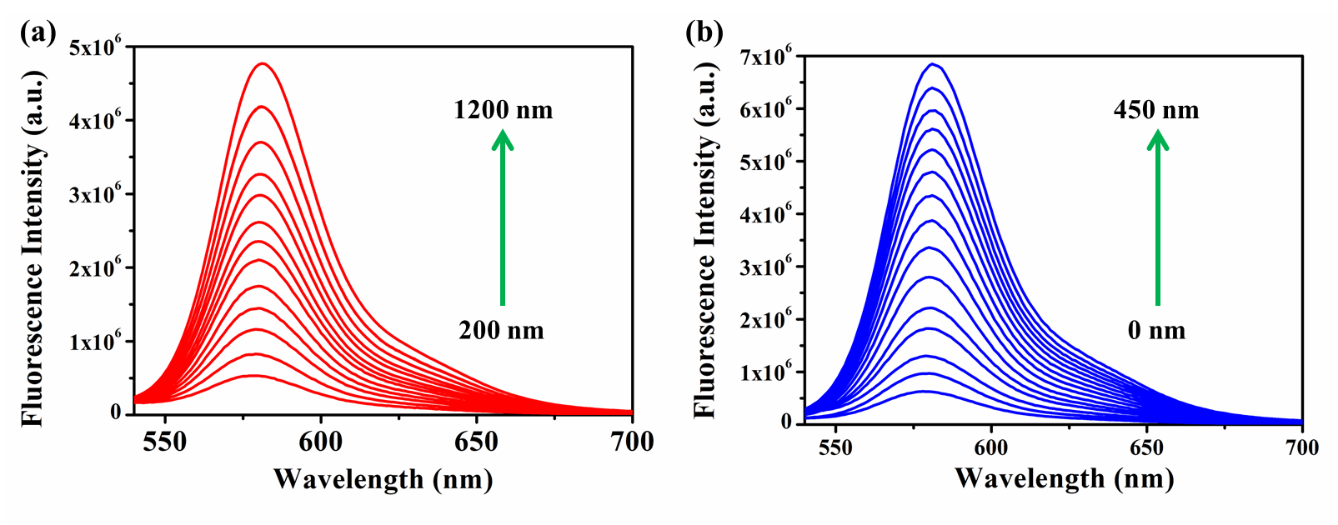


**Figure S25** Variation in emission responses upon addition of increasing concentration of (a) Al^3+^ (200-1200 nM) and (b) Fe^3+^ (0-450 nM).


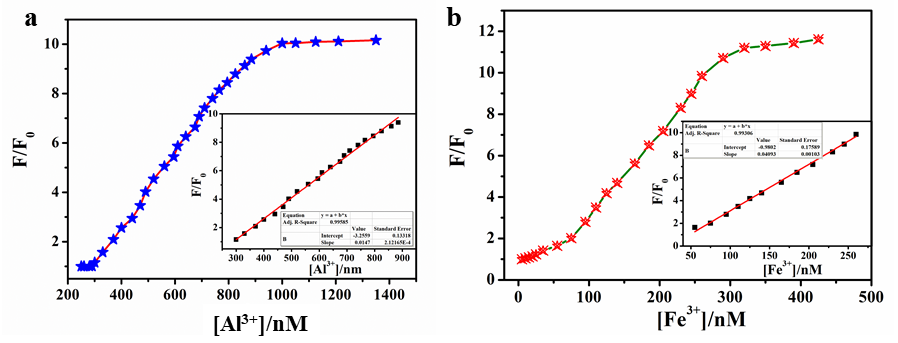


**Figure S26** Pot of variation in emission intensities vs concentration of Al^3+^ (a) and Fe^3+^ (b) ions. Inset: linear plot.





**Figure S27** Variation in absorption maxima of the receptor **L** upon addition of Al^3+^ and methylene blue.


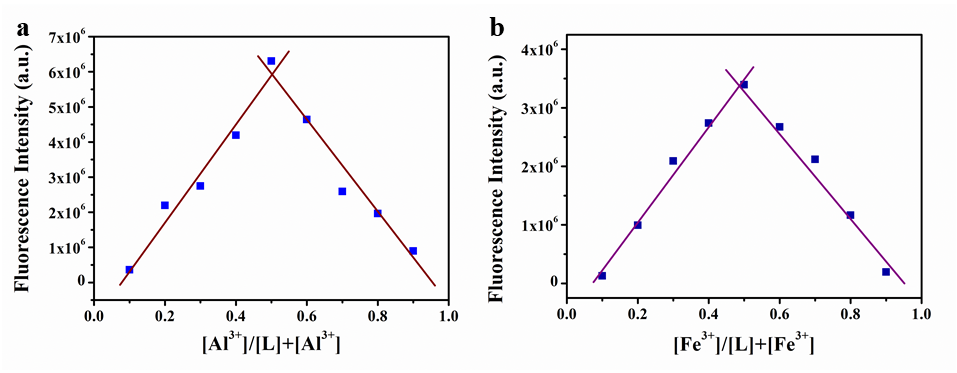


**Figure S28** Jobs plot analysis for the receptor L and Al^3+^ (a) and Fe^3+^ (b) ions.





**Figure S29** Variation in emission maxima of C_1_ and C_2_ with the change of pH.


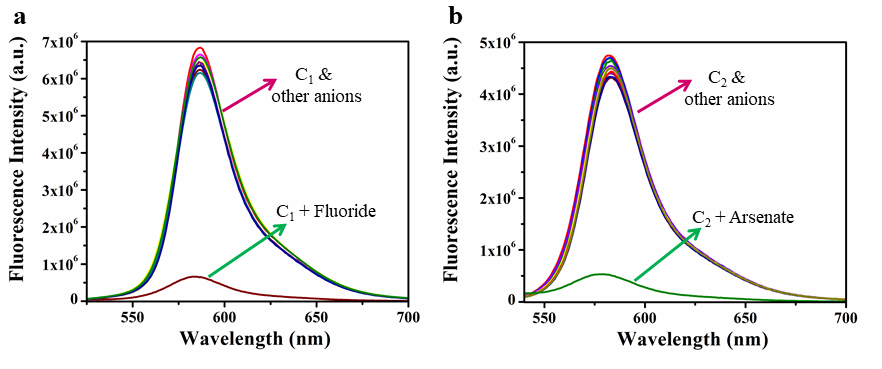


**Figure S30** Change in emission intensity of the probe C_1_ and C_2_ upon interaction with different anions.


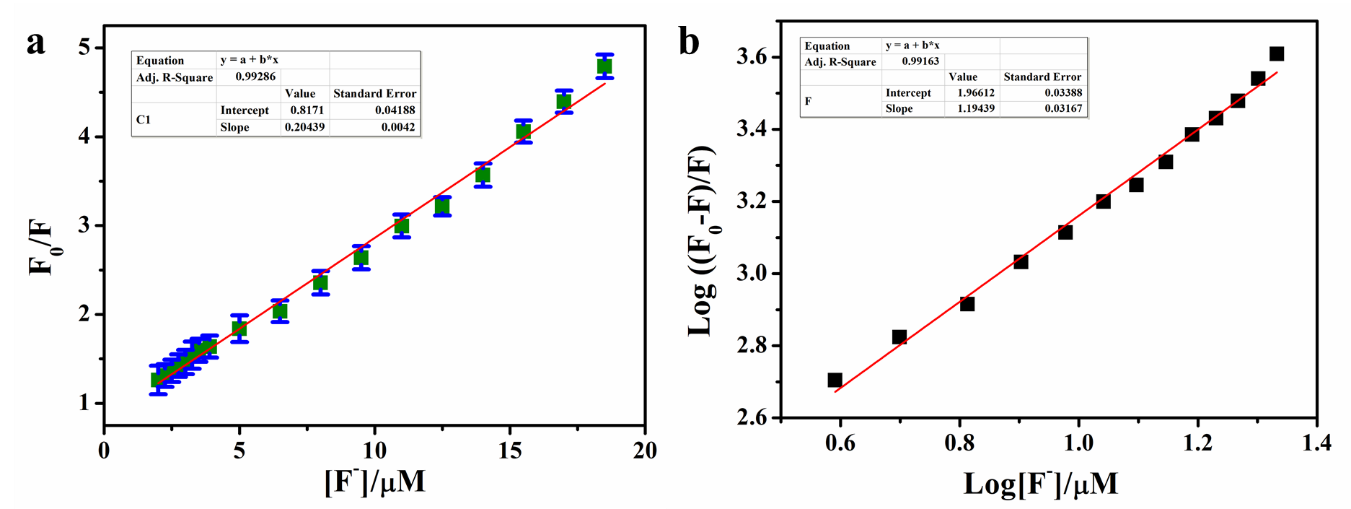


**Figure S31** (a) Stern-Volmer plot for C_1_-F^-^ system and (b) Plot of log $\frac{(F_{0}-F)}{F}$ vs log [F^-^].


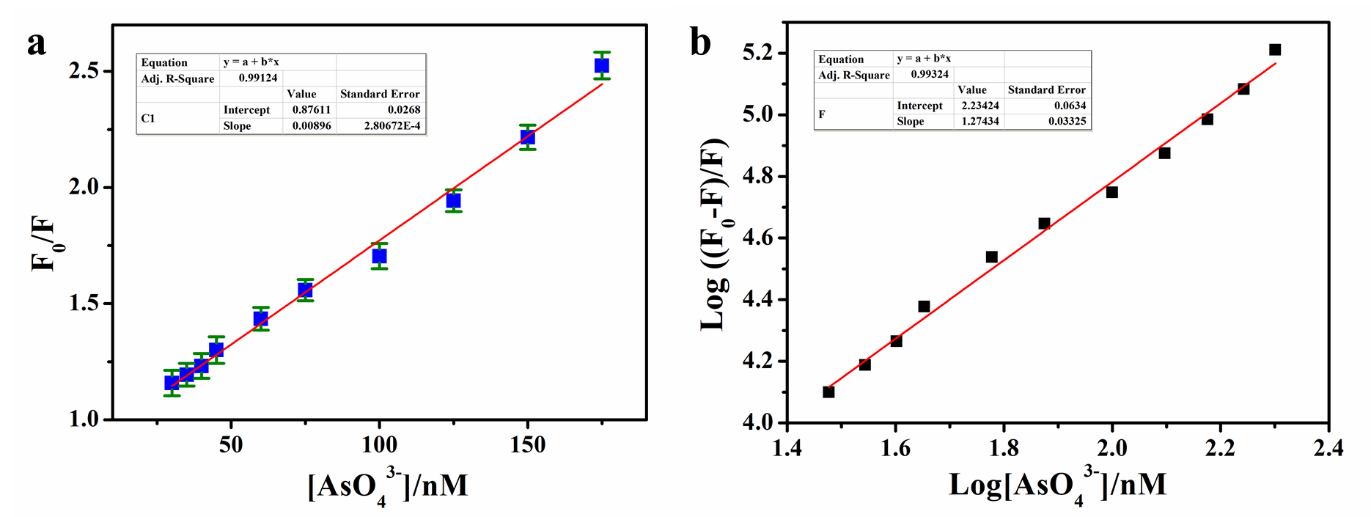


**Figure S32** (a) Stern-Volmer plot for C_2_-AsO_4_^3-^ system, (b*)* Plot of *log* $\frac{(F_{0}-F)}{F}$ vs *log* [AsO_4_^3-^].


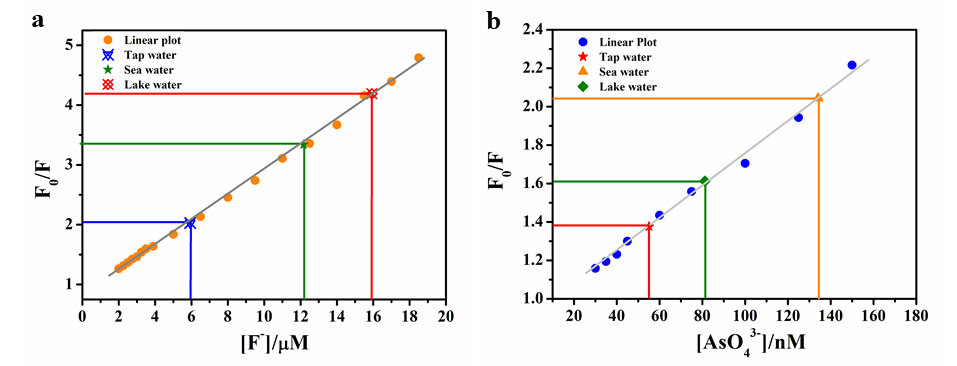


**Figure S33** Determination of unknown concentrations of (a) fluoride and (b) arsenate in tap water, lake water and sea water.


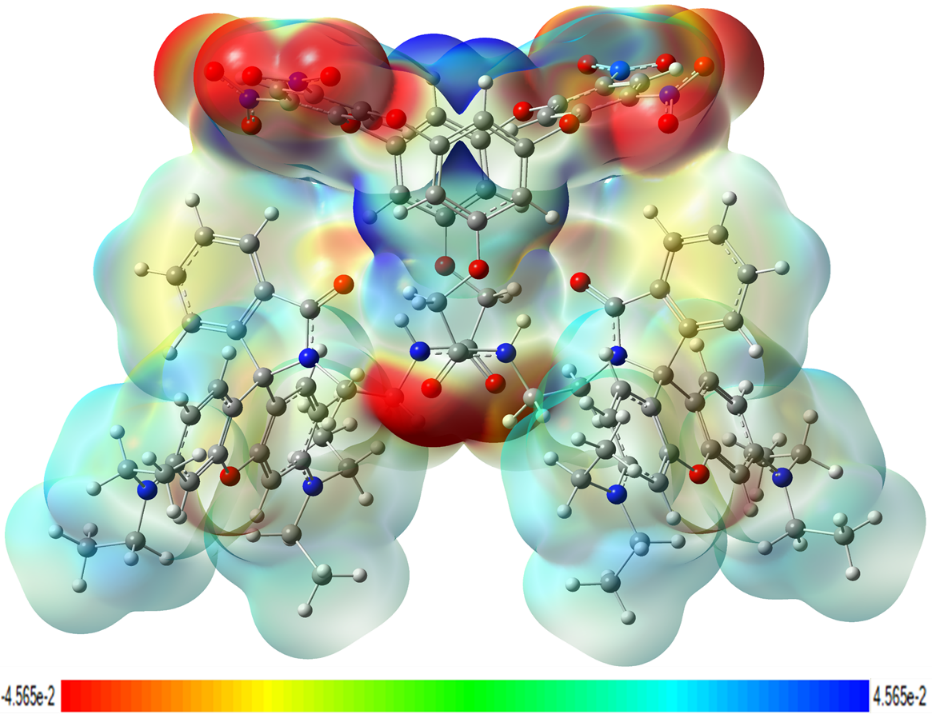


**Figure S34** Molecular Electrostatic potential (MESP) map of L. The red surface corresponds to a region of the lower potential, whereas the blue surface corresponds to the higher potential.


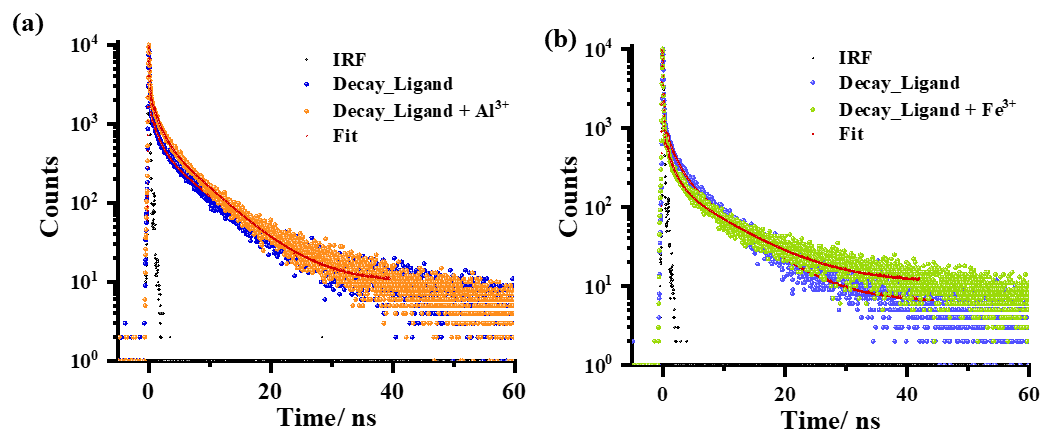


**Figure S35** Variation in fluorescent lifetime of ligand (L) before and after treatment of trivalent metal (a) Al^3+^ (b) Fe^3+^ at 498 nm.


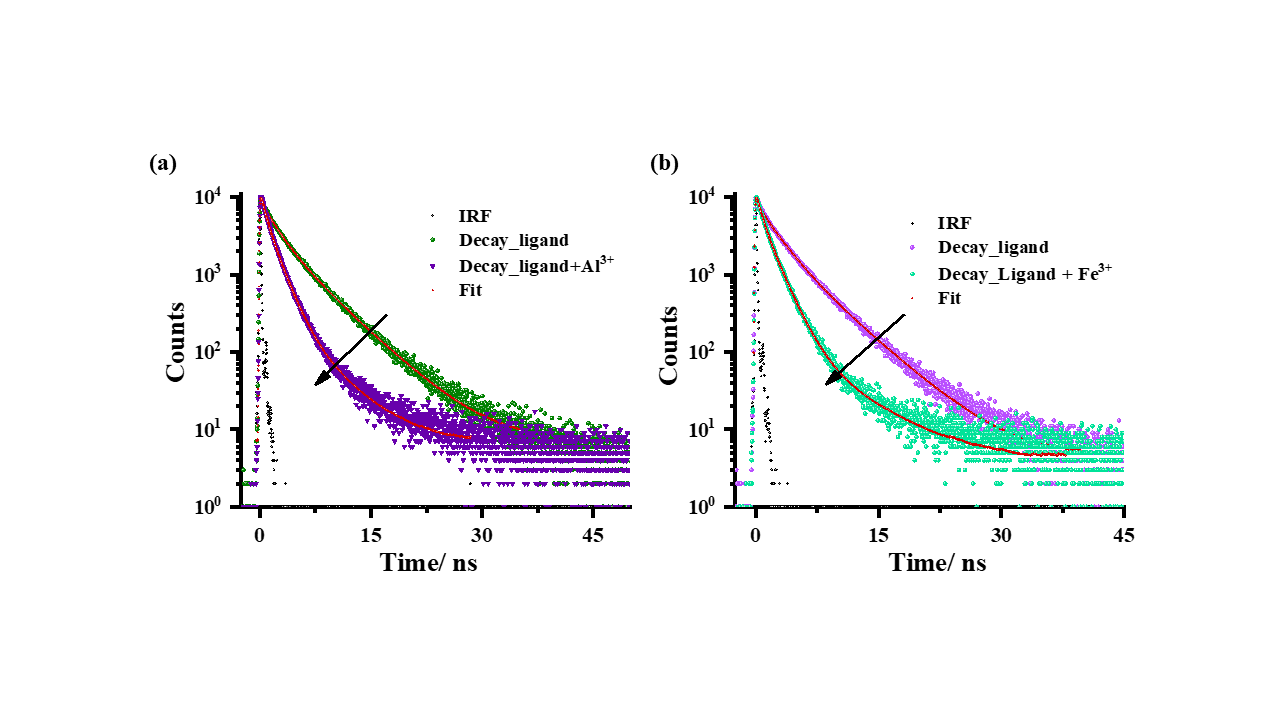


**Figure S36** Change in fluorescence lifetime of ligand (L) in the absence and presence of (a) Al^3+^ and (b) Fe^3+^ at 582 nm





**Figure S37** Comparison in FTIR spectra between L and L+M^3+^ (Al^3+^/Fe^3+^).


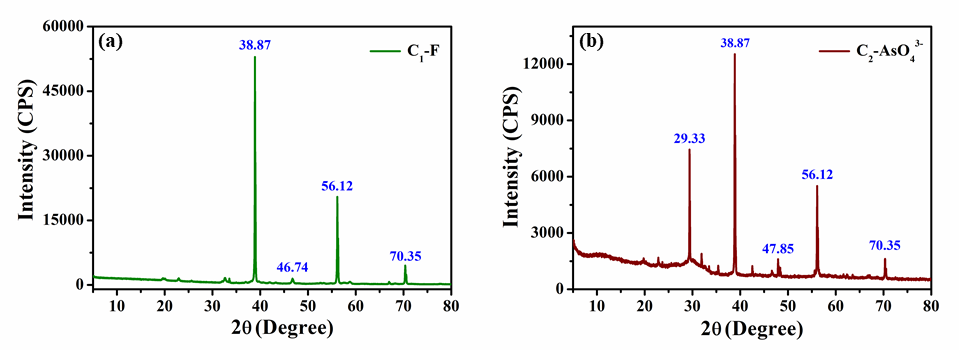


**Figure S38** PXRD analysis of (a)C_1_-F^-^ and (b) C_2_-AsO_4_^3-^ systems


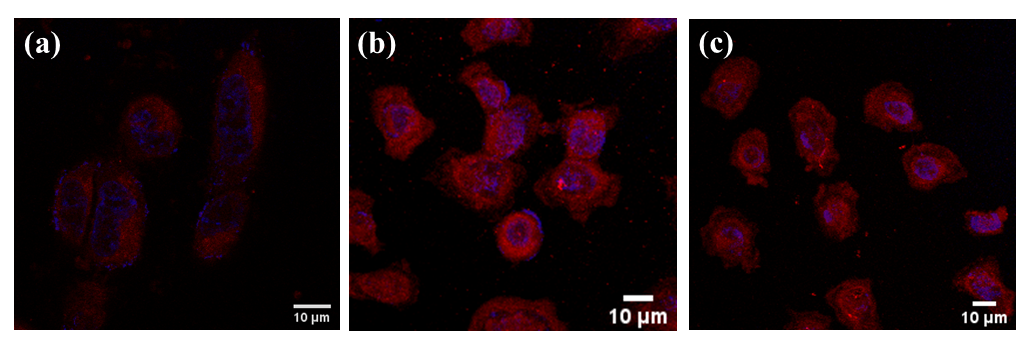


**Figure S39** Substantial enhancement in fluorescence intensity of (a) L incubated SUM159 cell line with the successive addition of (b) Al^3+^ and (c) Fe^3+^


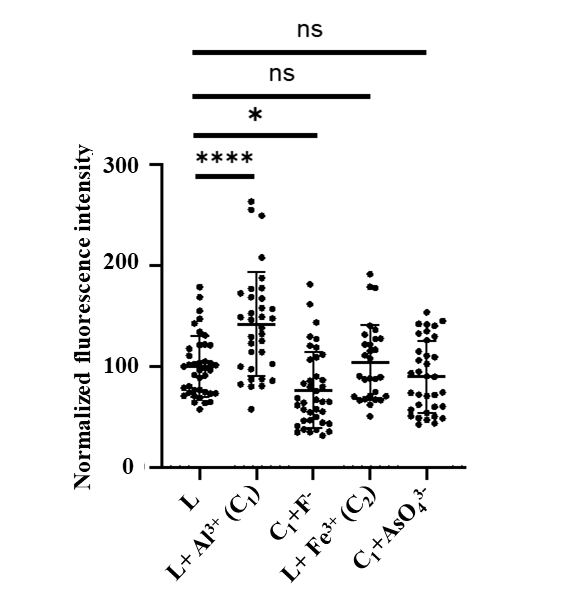


**Figure S40** Variation in fluorescence intensities of SUM159 cell line upon addition of L, C_1_, C_1_+fluoride, C_2_ and C_2_+arsenate

**Table S1** Crystallography and refinement details

| **DATA** | **Compound 1** |
| --- | --- |
| Moiety formula | 2(C_24_ H_12_ N_4_ O_14_), 2(C H_4_ O) |
| M (gmol^-1^) | 1224.83 |
| CCDC Number | 1985477 |
| Crystal dimensions (mm^3^) | 0.3 x 0.2 x 0.07 |
| Crystal system | triclinic |
| Space group | *P*$\bar{1}$ |
| *a* (Å) | 14.3488(9) |
| *b* (Å) | 15.0188(9) |
| *c* (Å) | 15.2848(10) |
| *α* (deg) | 97.162(5) |
| *β* (deg) | 92.241(4) |
| *γ* (deg) | 101.857(4) |
| V/Å^3^ | 3191.4(4) |
| Z | 2 |
| ρcalc (gcm^-3^) | 1.275 |
| θmax (°) | 25.00 |
| Radiation | MoKα |
| λ (Å) | 0.71073 |
| F(000) | 1256 |
| T (K) | 140(2) |
| Measured reflections | 11054 |
| Observed reflections (Io > 2σ(Io)) | 6294 |
| Parameters refined | 799 |
| R1 | 0.1041 |
| wR2 | 0.2917 |
| R1 (all data) | 0.1522 |
| wR2 (all data) | 0.3288 |
| GOOF | 1.036 |

**Table S2** Intermolecular interactions (Å, °) in the crystal structure of the compound 1.

| Motif number | Symmetry Code | Possible involved interactions | Geometry (Å, °) |
| --- | --- | --- | --- |
| I | x,y,z | O9-H9···O33 | 1.85, 160 |
| II | x,y,z | O14-H14···O32 | 1.83, 165 |
| III | -1-x,1-y,1-z | O32-H32···O13 | 2.28, 145 |
| IV | -1-x,1-y,1-z | O32-H32···O4 | 2.41, 139 |
| V | -1-x,-y,1-z | C14-H14A···O6 | 2.38, 173 |
| VI | -x,1-y,2-z | O19-H19···C26 | 2.58, 158 |
| VII | -x,1-y,1-z | C45-H45···O27 | 2.46, 160 |
| VIII | -x,-y,2-z | C38-H38···O21 | 2.44, 166 |
| IX | -x,1-y,1-z | C21···C26 | 3.169 |

**Table S3** Some important bond distances of the receptor **L** and **L-Al^3+^** system in Å around the active site.

|  | **L (GS)** | **L-Al^3+^ (GS)** |
| --- | --- | --- |
| C(63)-N(141) | 1.49 | 2.80 |
| C(70)-N(141) | 1.35 | 1.25 |
| C(121)-N(126) | 1.32 | 1.44 |
| C(70)-O(54) | 1.23 | 1.35 |
| C(121)-O(123) | 1.22 | 1.19 |
| C(15)-O(51) | 1.35 | 1.43 |
| C(152)-N(204) | 1.49 | 1.56 |
| C(159)-N(204) | 1.35 | 1.31 |
| C(122)-N(125) | 1.33 | 1.29 |
| C(159)-O(143) | 1.23 | 1.29 |
| C(122)-O(124) | 1.22 | 1.27 |
| C(29)-O(52) | 1.35 | 1.40 |
| C(156)-N(145) | 1.37 | 1.35 |
| C(148)-N(144) | 1.37 | 1.35 |
| C(59)-N(55) | 1.37 | 1.33 |
| C(67)-N(56) | 1.37 | 1.33 |

**Table S4** Excitation/Absorption energies (eV) and corresponding oscillator strengths (f), wave function and major contributions of L and its Al^3+^ complex calculated at HF/3-21G* based on the optimized ground state geometries.

| **Compound** | **Energy**  **[eV]** | **Oscillatory**  **Strength** | **Major Contribution of**  **transition** |
| --- | --- | --- | --- |
| L (ab) | 5.5 | 0.5286 | HOMO-20 to LUMO+1 (31%)  HOMO-17 to LUMO (14%)  HOMO-21 to LUMO (24%) |
| L (em) | 5.2 | 0.3452 | HOMO-21 to LUMO (52%)  HOMO-20 to LUMO (13%) |
| L-Al (ab) | 3.6 | 1.2540 | HOMO to LUMO+2 (93%) |
| L-Al (em) | 3.4 | 1.2208 | HOMO to LUMO+1 (91%) |

**Table S5** Result of recovery values for the detection of fluoride and arsenate in real water samples

| **Determination of fluoride** | | | | | | | |
| --- | --- | --- | --- | --- | --- | --- | --- |
| Sample | | Spiked Amount (μM) | | Detected amount (nM) | | Recovery (%) | |
| Tap Water | | 6 | | 5.94 ± 0.13 | | 99 | |
| Sea Water | | 12 | | 12.19 ± 0.27 | | 101 | |
| Lake Water | | 16 | | 15.92 ± 0.19 | | 99 | |
|  | |  | |  | |  | |
| **Determination of arsenate** | | | | | | | |
| Sample | Spiked Amount (nM) | | Detected amount (nM) | | Result using ICP-OES (nM) | | Recovery (%) |
| Tap Water | 55 | | 55.07 ±0.17 | | 55.17 | | 99 |
| Sea Water | 80 | | 81.20 ± 0.35 | | 80.13 | | 101 |
| Lake Water | 135 | | 134.13 ± 0.21 | | 135.24 | | 99 |

**Table S6** Variation in fluorescent average lifetime of ligand in the absence and presence of metal ions at 498 nm along with the lifetime of each component and its contribution.

| **Fluorescence lifetime measurement at λ_498 nm_** | | | | | |
| --- | --- | --- | --- | --- | --- |
| Entry | τ_1_ (Cont%) | τ_2_ (Cont%) | τ_3_ (Cont%) | τ_avg_/ ns | Chi. Sq. |
| Ligand only | 1.17 (14.17%) | 6.38 (43.56%) | 0.03 (42.27%) | 6.0 | 1.3 |
| Ligand + Al^3+^ | 1.21 (22.56%) | 6.0 (49.48%) | 0.04 (27.96%) | 5.6 | 1.5 |
| Ligand + Fe^3+^ | 1.30 (12.75%) | 8.29 (29.4%) | 0.03 (57.85%) | 7.8 | 1.3 |

**Table S7** Variation in fluorescence average lifetime of ligand in the absence and presence of metal ions at 582 nm along with the lifetime of each component and its contribution.

| Fluorescence lifetime measurement at λ_582 nm_ | | | | | |
| --- | --- | --- | --- | --- | --- |
| Entry | τ_1_ (Cont%) | τ_2_ (Cont%) | τ_3_ (Cont%) | τ_avg_/ ns | Chi. Sq. |
| Ligand only | 1.94 (35.48%) | 0.26 (3.55%) | 4.89 (60.97%) | 4.3 | 1.14 |
| Ligand + Al^3+^ | 0.50 (16.97%) | 1.56 (71.25%) | 4.71 (11.78%) | 2.5 | 1.18 |
| Ligand + Fe^3+^ | 0.56 (20.1%) | 1.6 (72.73%) | 6.09 (7.17%) | 2.7 | 1.19 |

Coordinates of the geometries optimized at HF/3-21G* of the structure L and its metal (L-Al^3+^) complex is given below.

**Coordinates of L**

C 3.28112100 5.47862700 -0.16150400

C 4.49325500 5.90268000 -0.70210800

C 4.55387200 6.25377700 -2.02701300

H 5.47592200 6.60090500 -2.43249200

C 3.46506700 6.14690700 -2.85986200

C 2.26408800 5.65876000 -2.34643800

C 2.19908300 5.36083500 -1.00177000

H 1.28037200 5.00921500 -0.60070300

C 0.09971400 4.70145300 -2.67435000

C -1.02464300 5.35888900 -2.24087300

H -1.11375700 6.41972500 -2.32314400

C -2.03124500 4.58522900 -1.68488900

C -1.92335300 3.23072000 -1.55520000

H -2.66414600 2.63647900 -1.06673000

C -0.78146900 2.59229900 -2.02942000

C 0.24302800 3.32831800 -2.58638100

H 1.14203900 2.86737500 -2.93384500

C -3.28097500 5.47862800 0.16252900

C -4.49309000 5.90251300 0.70331500

C -4.55369000 6.25309200 2.02835100

H -5.47572300 6.60009200 2.43397900

C -3.46488400 6.14582900 2.86115500

C -2.26393100 5.65784900 2.34752600

C -2.19892900 5.36049300 1.00272300

H -1.28023800 5.00901800 0.60147800

C -0.09966500 4.70030700 2.67489400

C -0.24340600 3.32730000 2.58546800

H -1.14263800 2.86628300 2.93225700

C 0.78082900 2.59158300 2.02766200

C 1.92300700 3.23013300 1.55430400

H 2.66367400 2.63610900 1.06536200

C 2.03126400 4.58446600 1.68535900

C 1.02486000 5.35786600 2.24208500

H 1.11423300 6.41860300 2.32532800

O 3.17228200 5.22174700 1.16462800

O 1.18603700 5.45848700 -3.14733700

O -3.17219100 5.22226800 -1.16369600

O -1.18588500 5.45718900 3.14835100

O 5.83725000 5.20161300 1.03300800

O 6.59579600 6.73231400 -0.36427800

O 4.83073800 6.70875300 -4.60974800

O 2.64958800 6.68758400 -4.94364900

O -5.83717600 5.20208500 -1.03199100

O -6.59551000 6.73253400 0.36568900

O -4.83051000 6.70696000 4.61130700

O -2.64935000 6.68563900 4.94514000

N 5.71470000 5.95592700 0.05233100

N 3.64687900 6.53450400 -4.23310600

N -5.71454300 5.95609300 -0.05109000

N -3.64666100 6.53287100 4.23455800

O -0.77785700 1.25031200 -1.86818200

O 0.77658500 1.24990300 1.86428600

O -6.53282600 -4.47917800 -0.93877900

O -3.19582000 0.81655600 0.09809400

N -8.01186100 -4.71485500 3.57159900

N -5.05980500 -4.89393000 -5.43845400

C -6.64928600 -3.82971200 0.26979800

C -7.25001900 -4.55288700 1.28240600

C -7.42125800 -4.00639900 2.55165900

C -6.95414300 -2.68768800 2.74051200

C -6.36958100 -1.99317700 1.71500100

C -6.20105600 -2.54060300 0.44835900

C -5.54908100 -1.75109700 -0.67148200

C -5.42110300 -2.58821900 -1.92978500

C -5.90768500 -3.87290800 -2.00619200

C -5.79567500 -4.64197900 -3.14891200

C -5.17767600 -4.14318000 -4.29256100

C -4.67703000 -2.82508800 -4.21713100

C -4.80108200 -2.08815600 -3.06940600

C -4.17675000 0.13484500 -0.22192600

C -5.48826100 0.62890400 -0.68329000

C -5.90761100 1.92985800 -0.86519200

C -7.19786800 2.13443900 -1.31774300

C -8.02886700 1.04814200 -1.57515100

C -7.59070500 -0.25545500 -1.39462300

C -6.30290000 -0.44903800 -0.94431700

C -8.31261200 -4.10606000 4.87067800

C -7.13441800 -4.20895600 5.85677900

C -8.37916100 -6.12526900 3.41913000

C -9.79227800 -6.30670800 2.83440800

C -5.68531500 -6.21301700 -5.56399600

C -4.78095000 -7.34695700 -5.04609500

C -4.29016300 -4.43118200 -6.59718400

C -5.13921700 -3.59483000 -7.57215400

H -7.57873600 -5.53607500 1.03623100

H -7.03838300 -2.21679400 3.69416400

H -6.02768700 -0.99358900 1.89547500

H -6.18746400 -5.63156300 -3.09849300

H -4.20355200 -2.37845800 -5.06243500

H -4.40881500 -1.09103400 -3.04839800

H -5.26289900 2.76356100 -0.68336500

H -7.54422100 3.13558500 -1.47269600

H -9.02664900 1.22327100 -1.92575000

H -8.23304500 -1.08680200 -1.60238800

H -9.17308700 -4.61888200 5.28177000

H -8.60434700 -3.07411500 4.73447000

H -7.39520900 -3.75805500 6.80819500

H -6.25942900 -3.70845100 5.46223400

H -6.87898200 -5.24807100 6.02769600

H -7.64695500 -6.62912500 2.80354700

H -8.33167100 -6.58541300 4.39810700

H -10.03229100 -7.36052700 2.74288800

H -10.52753600 -5.84200000 3.48079100

H -9.86427600 -5.84738800 1.85698000

H -5.90357400 -6.37524700 -6.61211100

H -6.63376400 -6.21812800 -5.04488200

H -3.85136600 -7.36860000 -5.60258100

H -4.54386300 -7.20076300 -4.00021600

H -5.27295600 -8.30676300 -5.16136700

H -3.42835300 -3.86916800 -6.26571700

H -3.91012200 -5.30630800 -7.10907600

H -5.53472600 -2.71599500 -7.07914700

H -4.54222800 -3.27945100 -8.42105600

H -5.97449600 -4.18021900 -7.93812600

C -0.32953400 0.35720400 2.08403700

C 0.32917100 0.35807300 -2.08539600

H 0.64303600 0.36614100 -3.11955200

H 1.16600200 0.60138400 -1.44390100

H -0.64179400 0.36595100 3.11869300

H -1.16745300 0.59959100 1.44364200

C -0.17689800 -1.04520900 -1.75269300

C 0.17681000 -1.04603800 1.75160700

O 0.52513000 -2.01600300 -2.02015700

O -0.52508300 -2.01690300 2.01919400

N 1.36656700 -1.08412800 1.16266300

N -1.36659200 -1.08340800 -1.16360200

H -1.82305100 -0.21654400 -0.96480300

H 1.82276700 -0.21720100 0.96352400

C -2.03001700 -2.33974800 -0.79984400

C 2.03007600 -2.34037600 0.79875100

H -1.30621900 -2.99725500 -0.34401300

H -2.43815300 -2.81474600 -1.68026300

H 2.43801600 -2.81561600 1.67912700

H 1.30641000 -2.99776800 0.34254000

C -3.13704800 -2.08148200 0.23232100

C 3.13727000 -2.08168600 -0.23312800

H -3.58417600 -3.02424500 0.51013000

H -2.68786100 -1.65289300 1.11290100

H 3.58451500 -3.02430100 -0.51125500

H 2.68814100 -1.65274000 -1.11357100

N -4.22580800 -1.21306200 -0.24152600

O 6.53289900 -4.47940200 0.93839900

O 3.19577500 0.81625700 -0.09836200

N 8.01336700 -4.71393600 -3.57156800

N 5.05856900 -4.89526900 5.43754200

C 6.64968500 -3.82965200 -0.26999300

C 7.25079200 -4.55254900 -1.28257800

C 7.42238900 -4.00575800 -2.55165100

C 6.95523000 -2.68704100 -2.74035000

C 6.37029300 -1.99280900 -1.71486300

C 6.20140900 -2.54053800 -0.44840000

C 5.54902600 -1.75133100 0.67141800

C 5.42075600 -2.58874000 1.92950100

C 5.90740800 -3.87341000 2.00576600

C 5.79511700 -4.64274400 3.14828200

C 5.17672800 -4.14425200 4.29185300

C 4.67600000 -2.82618200 4.21656500

C 4.80034200 -2.08898500 3.06904200

C 4.17669200 0.13460000 0.22178400

C 5.48802700 0.62867100 0.68366600

C 5.90724500 1.92961400 0.86595300

C 7.19734700 2.13418900 1.31895600

C 8.02833200 1.04789700 1.57641900

C 7.59030700 -0.25569200 1.39550400

C 6.30265700 -0.44926900 0.94475600

C 8.31448500 -4.10482000 -4.87041200

C 7.13661200 -4.20758000 -5.85691000

C 8.38073100 -6.12435500 -3.41930400

C 9.79368000 -6.30581500 -2.83418000

C 5.68414500 -6.21433400 5.56297400

C 4.78002600 -7.34822900 5.04454300

C 4.28852700 -4.43285000 6.59613700

C 5.13720500 -3.59664400 7.57155900

H 7.57951000 -5.53576800 -1.03652400

H 7.03973200 -2.21592300 -3.69386800

H 6.02837800 -0.99320600 -1.89521600

H 6.18699500 -5.63228800 3.09775900

H 4.20222400 -2.37978300 5.06182400

H 4.40799200 -1.09189500 3.04812800

H 5.26254400 2.76331800 0.68408100

H 7.54359000 3.13532600 1.47420500

H 9.02599500 1.22302000 1.92736200

H 8.23263500 -1.08703900 1.60330600

H 9.17512900 -4.61748200 -5.28134900

H 8.60609600 -3.07288500 -4.73387400

H 7.39767100 -3.75644200 -6.80814100

H 6.26146000 -3.70723100 -5.46252900

H 6.88130800 -5.24667600 -6.02814600

H 7.64837200 -6.62840800 -2.80406400

H 8.33358400 -6.58428200 -4.39840000

H 10.03374600 -7.35963700 -2.74282200

H 10.52910300 -5.84090300 -3.48022800

H 9.86533500 -5.84671000 -1.85662700

H 5.90209900 -6.37678500 6.61111900

H 6.63275100 -6.21925300 5.04414700

H 3.85027600 -7.37007200 5.60074400

H 4.54324200 -7.20181600 3.99862700

H 5.27207400 -8.30802200 5.15974500

H 3.42677000 -3.87083700 6.26452800

H 3.90840300 -5.30812500 7.10771400

H 5.53279100 -2.71766400 7.07887200

H 4.53992600 -3.28150800 8.42034700

H 5.97242100 -4.18204300 7.93765800

N 4.22584500 -1.21331200 0.24119100

**Coordinates of L-Al^3+^**

C -6.73486200 1.65192500 1.62113800

C -7.01322400 0.48194600 2.30405800

C -6.23690800 0.11512900 3.38211200

H -6.44201100 -0.80782600 3.87643600

C -5.16650700 0.87972500 3.77580700

C -4.91702900 2.08959600 3.13874600

C -5.70726000 2.45571600 2.08147500

H -5.52869600 3.38475300 1.59023800

C -2.83888900 3.24139000 2.70692100

C -2.87934400 4.40721600 1.97077200

H -3.68182500 5.10183000 2.09850400

C -1.83093300 4.69428800 1.11859200

C -0.73041800 3.86308200 1.02263100

H 0.07318100 4.14342800 0.37683900

C -0.71519900 2.74001900 1.81745800

C -1.74991500 2.39198000 2.65140400

H -1.73098100 1.52732300 3.27628700

C -2.38124800 5.95949700 -0.89257300

C -1.62421900 6.48386500 -1.93067300

C -2.21197100 6.73123900 -3.14454300

H -1.64813000 7.17052200 -3.93767900

C -3.51564600 6.35186500 -3.39302600

C -4.23915000 5.71472700 -2.40187300

C -3.66914700 5.57738000 -1.14547600

H -4.26157800 5.16244400 -0.36292900

C -5.58702200 3.80820500 -2.16385200

C -4.61372300 2.89590400 -2.51938900

H -3.86298300 3.14720900 -3.23717700

C -4.59560300 1.67498600 -1.89291000

C -5.54710300 1.33546000 -0.96231600

H -5.50770700 0.37867700 -0.48742700

C -6.51455200 2.26312000 -0.63966400

C -6.55463500 3.51261100 -1.22459300

H -7.28991700 4.23516200 -0.94209000

O -7.36582400 1.98077800 0.44643600

O -3.90108300 2.92445300 3.55629400

O -1.84247900 5.86695700 0.37258300

O -5.46239800 5.12592600 -2.63172000

O -8.97211200 -0.02077000 1.21067300

O -7.84998800 -1.64239200 2.22158300

O -4.80026500 -0.46135500 5.59047300

O -3.08763300 0.64648600 4.76204300

O 0.43617300 5.88543800 -1.08842200

O 0.29240800 7.60560800 -2.46471100

O -3.18118200 6.83197900 -5.60054500

O -5.25551600 6.55162700 -4.87390300

N -8.03397500 -0.44293300 1.87828800

N -4.30247500 0.33694700 4.79478600

N -0.20664900 6.70128000 -1.80412900

N -4.03570500 6.59367500 -4.71959100

O 0.47402000 1.93899300 1.86793000

O -3.46849300 0.84932100 -2.02520400

O 8.69987200 -0.21484000 -0.34902800

O 2.11596600 0.51939600 -0.09876500

N 8.33442200 -1.93042800 -4.74031600

N 9.65594300 1.55454400 3.93132000

C 8.00333200 -0.28355500 -1.51628000

C 8.50059900 -1.08574700 -2.49903600

C 7.85819900 -1.17194500 -3.75097800

C 6.66040400 -0.38999600 -3.91302200

C 6.18681300 0.39677800 -2.92397700

C 6.83595100 0.49337600 -1.66584100

C 6.41821000 1.31534700 -0.61285200

C 7.17303500 1.38411900 0.56029500

C 8.34422100 0.60740000 0.67681600

C 9.15731800 0.63778000 1.76929900

C 8.85757600 1.47344600 2.86536300

C 7.64182600 2.23898600 2.77489300

C 6.85201200 2.19107500 1.68149000

C 3.46452200 0.71006200 -0.06684100

C 3.93128200 1.97646000 -0.68217000

C 2.98794500 2.94440200 -1.00833700

C 3.36203100 4.19634300 -1.45596600

C 4.70534400 4.48683100 -1.60927700

C 5.65531400 3.52470300 -1.31676900

C 5.28430100 2.27224300 -0.84594000

C 7.74469600 -1.94618200 -6.09822500

C 6.62705400 -2.99319900 -6.22812500

C 9.52681500 -2.79347600 -4.57081300

C 10.83332000 -2.03667100 -4.86091800

C 10.95650700 0.84799200 3.99966100

C 10.80981100 -0.57536400 4.56105000

C 9.33430700 2.37885000 5.11885800

C 9.81587700 3.83011500 4.96041200

H 9.40703600 -1.60464400 -2.29358400

H 6.13142400 -0.42860500 -4.83759800

H 5.30037500 0.97283900 -3.08958500

H 10.01808800 0.01169400 1.75602600

H 7.36264800 2.87657400 3.58189400

H 5.95519200 2.77240300 1.65697900

H 1.95195600 2.70266100 -0.90990800

H 2.61301600 4.93422600 -1.65952900

H 5.01458100 5.45140100 -1.95696000

H 6.69527600 3.74534400 -1.45055400

H 8.54497700 -2.17651600 -6.78626200

H 7.38866700 -0.95995300 -6.35477200

H 6.23965700 -2.99679600 -7.23990100

H 5.81343800 -2.78034100 -5.54541200

H 7.00766600 -3.98229200 -6.00592700

H 9.53088600 -3.21120700 -3.57513700

H 9.41863200 -3.61981000 -5.25862400

H 11.67866200 -2.70400500 -4.74453700

H 10.83703600 -1.65893400 -5.87567600

H 10.95891300 -1.19954300 -4.18629400

H 11.60062400 1.43225700 4.64058600

H 11.41372600 0.83700000 3.02176200

H 10.38864100 -0.54819800 5.55843500

H 10.16496300 -1.17789300 3.93372100

H 11.78120100 -1.05167200 4.61560700

H 8.27338800 2.33919900 5.31531700

H 9.82583400 1.91767300 5.96324200

H 9.33654400 4.31082400 4.11689000

H 9.58910800 4.39509400 5.85656700

H 10.88665300 3.85923600 4.80324400

C -2.42629600 1.23757300 -1.11247600

C 1.57537300 2.60192800 2.61682500

H 1.93909100 3.43319200 2.03666000

H 1.17221100 2.93997200 3.56082900

H -1.89045100 2.10401600 -1.47359700

H -2.82726900 1.45707500 -0.13323900

C 2.67530900 1.58382900 2.82032100

C -1.45312800 0.10427300 -0.97286600

O 3.79652600 1.88811800 3.09040100

O -0.50078800 0.29283300 -0.14824900

N -1.65570000 -0.95089700 -1.67867200

N 2.20576300 0.23657600 2.64592000

H 1.52865600 0.01048600 3.36889600

H -2.49813800 -0.92217300 -2.23414300

C 3.29997500 -0.83117300 2.65868400

C -0.82643100 -2.16918000 -1.81654700

H 2.89301800 -1.68509000 3.17793800

H 4.13835700 -0.43011600 3.20444100

H -1.48143200 -2.94366900 -2.17133800

H -0.07396900 -1.98344800 -2.57195500

C 3.74498100 -1.23004200 1.24356900

C -0.10853900 -2.65002800 -0.54711200

H 4.55302600 -1.93890600 1.34599700

H 2.93187200 -1.72211300 0.72239600

H 0.25558600 -3.64624100 -0.75418700

H 0.74204100 -2.02785100 -0.33507800

N 4.25494800 -0.06342000 0.52170800

O -2.50468600 -4.95822100 -1.70240200

O 0.12755600 -1.11323700 1.89342200

N 0.28055200 -8.75873200 -0.97868100

N -6.85198600 -3.03602600 -2.06411400

C -1.65866700 -5.65986600 -0.84812700

C -1.11387900 -6.82145500 -1.32946100

C -0.25419100 -7.59731400 -0.53265100

C 0.01483200 -7.09889800 0.76768700

C -0.54532800 -5.93445300 1.21749500

C -1.40255000 -5.17618600 0.42317100

C -2.00174100 -3.86767900 0.85386400

C -3.27444700 -3.56616300 0.08633500

C -3.48613100 -4.14870300 -1.14954800

C -4.62796800 -3.95911400 -1.88620000

C -5.69586200 -3.19655300 -1.37960500

C -5.49728000 -2.62631700 -0.09582600

C -4.32450300 -2.80283300 0.59092700

C -0.73194600 -2.07213700 1.75451500

C -1.55444300 -2.59874300 2.82899000

C -1.70162400 -2.15159900 4.13008000

C -2.55004200 -2.86088800 4.95757900

C -3.20578800 -3.99683500 4.48490900

C -3.06143200 -4.42709000 3.17297000

C -2.23237800 -3.70370000 2.34666800

C 1.06543600 -9.65189400 -0.10646200

C 2.56287800 -9.30141600 -0.10913700

C 0.06267700 -9.24508100 -2.35359800

C -1.22692900 -10.07335700 -2.48608400

C -7.11606400 -3.74594300 -3.33050600

C -6.60446300 -2.96610600 -4.55326700

C -7.96440500 -2.20495400 -1.56319800

C -8.93280900 -2.97939800 -0.65350500

H -1.40486900 -7.12690300 -2.30756200

H 0.66314500 -7.63777400 1.42075100

H -0.32290400 -5.60777100 2.21429000

H -4.68672300 -4.45599300 -2.82703200

H -6.30386600 -2.14374400 0.40604900

H -4.24746500 -2.38691000 1.57461000

H -1.20230200 -1.27497100 4.48494500

H -2.71618200 -2.53184100 5.96192200

H -3.84737900 -4.54156900 5.14774500

H -3.59014100 -5.28427500 2.81070900

H 0.92813800 -10.65974000 -0.47220900

H 0.66513900 -9.63090800 0.89705000

H 3.10939100 -9.99053200 0.52383900

H 2.72711000 -8.29381400 0.25337900

H 2.96301900 -9.37042300 -1.11328800

H 0.05527000 -8.40863700 -3.03853100

H 0.91605900 -9.85469400 -2.61683600

H -1.34243500 -10.42332700 -3.50508800

H -1.18989100 -10.93644100 -1.83310700

H -2.09741400 -9.48679900 -2.22071900

H -8.18562100 -3.87977600 -3.40693300

H -6.68251500 -4.73514400 -3.29361700

H -7.07865900 -1.99355100 -4.60444000

H -5.53193200 -2.82143700 -4.50265600

H -6.83580900 -3.50562800 -5.46387500

H -7.57273300 -1.34115400 -1.04464500

H -8.49536400 -1.83012500 -2.42748800

H -8.42388700 -3.37557400 0.21399600

H -9.71460900 -2.31506700 -0.30727100

H -9.38733300 -3.79944400 -1.19469500

N -0.94629100 -2.73473700 0.65010600

Al 0.85012000 0.27679100 1.03308800

**References:**

1 S. Dey, A. Kumar, A. Mahto, I. H. Raval, K. M. Modi, S. Haldar and V. K. Jain, *Sensors Actuators B. Chem.*, 2020, **317**, 128180.
